# Early Neolithic Water Wells Reveal the World's Oldest Wood Architecture

**DOI:** 10.1371/journal.pone.0051374

**Published:** 2012-12-19

**Authors:** Willy Tegel, Rengert Elburg, Dietrich Hakelberg, Harald Stäuble, Ulf Büntgen

**Affiliations:** 1 Institute for Forest Growth IWW, University of Freiburg, Freiburg, Germany; 2 Archaeological Heritage Office Saxony, Dresden, Germany; 3 Swiss Federal Research Institute WSL, Birmensdorf, Switzerland; 4 Oeschger Centre for Climate Change Research, Bern, Switzerland; University of Oxford, United Kingdom

## Abstract

The European Neolithization ∼6000−4000 BC represents a pivotal change in human history when farming spread and the mobile style of life of the hunter-foragers was superseded by the agrarian culture. Permanent settlement structures and agricultural production systems required fundamental innovations in technology, subsistence, and resource utilization. Motivation, course, and timing of this transformation, however, remain debatable. Here we present annually resolved and absolutely dated dendroarchaeological information from four wooden water wells of the early Neolithic period that were excavated in Eastern Germany. A total of 151 oak timbers preserved in a waterlogged environment were dated between 5469 and 5098 BC and reveal unexpectedly refined carpentry skills. The recently discovered water wells enable for the first time a detailed insight into the earliest wood architecture and display the technological capabilities of humans ∼7000 years ago. The timbered well constructions made of old oak trees feature an unopened tree-ring archive from which annually resolved and absolutely dated environmental data can be culled. Our results question the principle of continuous evolutionary development in prehistoric technology, and contradict the common belief that metal was necessary for complex timber constructions. Early Neolithic craftsmanship now suggests that the first farmers were also the first carpenters.

## Introduction

**Figure 1:**
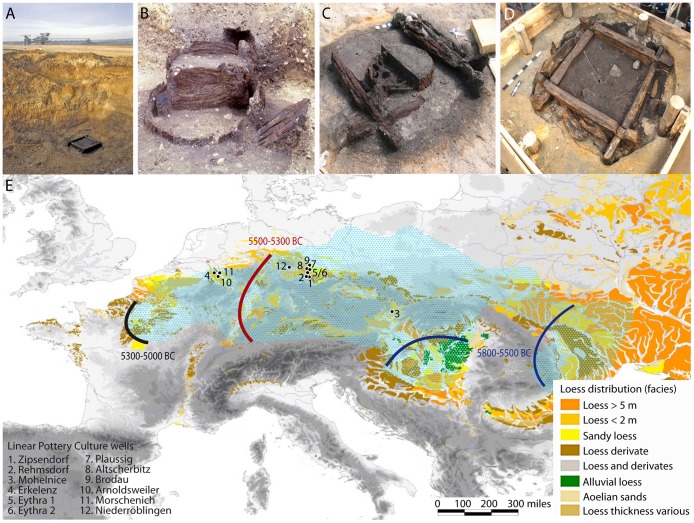
Wooden well constructions and Neolithization. LBK wells from (*A*) Eythra 1, (*B*) Eythra 2, (*C*) Brodau 1, and (*D*) Altscherbitz. (*E*) Central European loess distribution [20] with the superimposed phases of expansion of the LBK (lines), based on ^14^C dates [22], and the maximum extension of the LBK (light blue) along with the 12 known early Neolithic wells featuring waterlogged wood preservation.

After the last Ice Age ∼12,000 BP, the Central European landscape changed from steppes to dense woodlands [1], and the climate became warmer and likely also wetter [2], [3], [4]. During the 6th millennium BC, sedentariness became the dominant lifestyle of the Central European population, which began to cultivate plants, raise livestock, produce ceramics, and exploit the woodlands as a timber resource [5]–[8]. This transformation marked the onset of the Neolithic period, and for the first time, human societies began to transform their natural environment into a cultural landscape [8]–[10]. Sedentism required permanent building structures for living and storage. Consequently, innovations in tool manufacture and woodworking techniques were crucial for setting up the required settlement infrastructure. The Neolithization is associated with a profound shift in prehistoric society [11]–[13] and well represented by a homogeneous material culture across most of the European continent. The first Central European farmers, who likely immigrated from the Balkan Peninsula and the Carpathian Basin ∼7,500 years ago [6], [14]–[18] (Figure 1), left a uniform archaeological record of settlement structures with longhouses, pottery and stone tools [19], called the Linear Pottery Culture (LBK; Linearbandkeramik) after the typically decorated ceramics [14]. LBK settlements rapidly spread across the continent's fertile loess regions [20], [21], but a detailed understanding of the subsistence strategies and technological skills of the farmers is still hindered by a lack of sufficiently preserved and precisely dated organic artifacts, although there is some botanical and zoological evidence [8], [10].

A precise chronological framework beyond radiocarbon dates and LBK pottery typology is required for a deeper understanding of the Neolithization process [22]–[24]. Dendrochronological dating ultimately depends on well-preserved construction timber from waterlogged environments [25], [26]. Whereas the LBK longhouses throughout Europe have left only ground-plans in the soil, wooden well constructions survived for thousands of years below ground water level (Figure 1). The LBK timbers can be calendar-dated against continuous tree-ring chronologies from subfossil oak trees buried in river deposits that span most of the Holocene [27], [28].

## Results and Discussion

**Figure 2:**
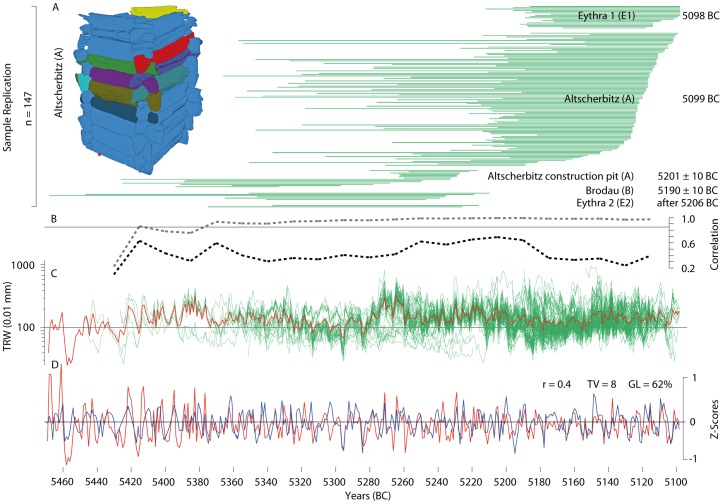
Tree-ring samples and chronologies. (*A*) Temporal distribution of 147 oak ring width series, indicating the lengths of the individual tree-ring sequences, and the youngest felling date per well construction, based on the presence of waney edges (annually precise) or sapwood (±10 years). The inset shows a 3D reconstruction of the wooden lining of the well from Altscherbitz displaying each tree using a different color. (*B*) The Expressed Population Signal (dotted line grey) and the inter-series correlation (dotted line black) calculated over 50 years lagged by 25 years along all of the individual samples. (*C*) Single ring width measurements (green) and their mean (red). (*D*) Absolute dating of the new Saxon oak chronology (red) against the reference chronology from the Main River Valley [30] after 10-year low-pass filtering (*r* = correlation coefficient, *TV* = T-value, *GL* = Gleichläufigkeit).

Here, we present annually resolved and absolutely dated tree-ring samples from 151 oak (*Quercus spp.*) timbers from four water well constructions excavated in Altscherbitz, Brodau and Eythra (denoted by A, B, E1 and E2, (Figure 1). The individual ring width measurement series cover 371 years from 5469 to 5098 BC (Figure 2), and all of the timbers originate from at least 46 mature trees (Text S1). The individual felling dates of wells A, B, E1 and E2 correspond to construction activities in 5099, at 5190±10, in 5098 and after 5206 BC, respectively (Figure 2).

**Figure 3:**
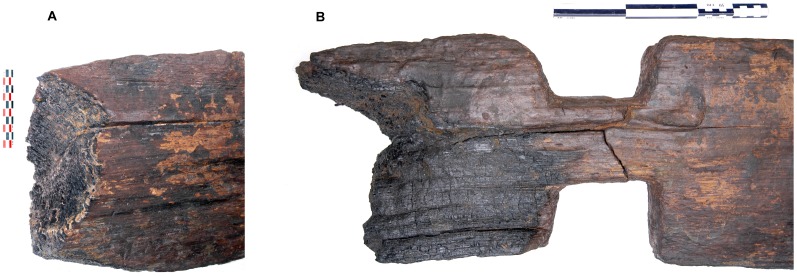
Charred end grain surfaces at terminal ends of oak timbers from well A (*A, B*). The timbers were cut to length using fire.

The early Neolithic settlers felled mature oak trees up to 300 years old and measuring 1 m maximum in diameter. Stone adzes with transversely hafted blades were used, and the felling cuts were placed just above breast height. The Neolithic logging technique can be convincingly reconstructed according to ethnological evidence [29]. The logs were split first in half with wooden wedges that were hammered in using wooden mauls. Such timber conversion has been verified experimentally for prehistoric times [30], [31]. There is evidence on the timber surfaces that the log halves were cut to their final length by adze work and the use of burning charcoals (Figure 3). Molding by fire is also a common technique in Neolithic logboat construction [32]. The trimmed halves were then again radially or tangentially split into the final timbers. After smoothing the split timber surfaces using adzes, the boards were ready for constructional use.

**Figure 4:**
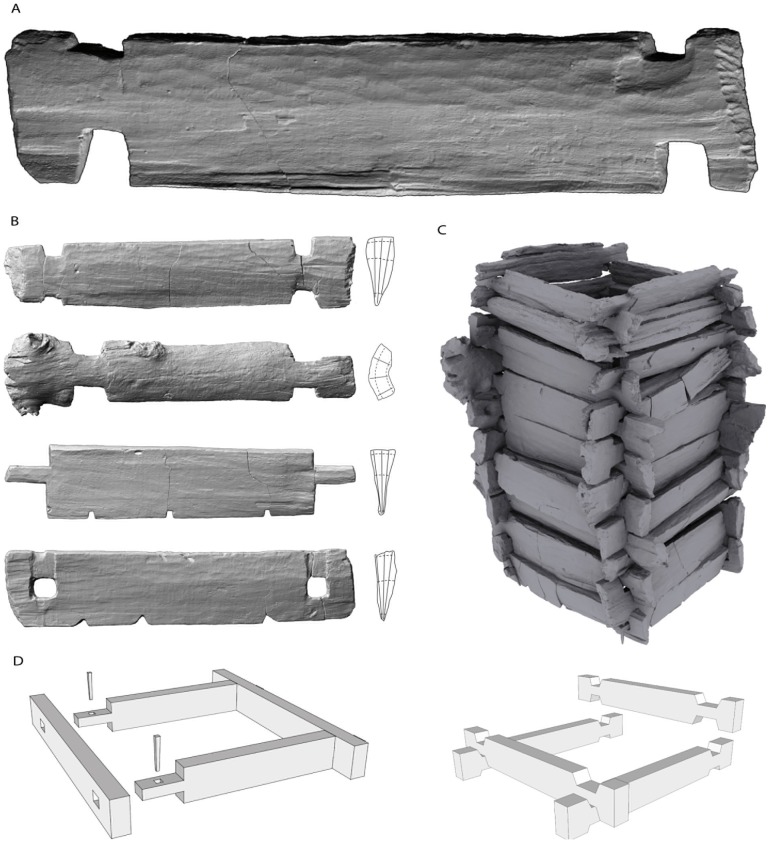
Early Neolithic craftsmanship from well A. 3D laser rendering of (*A*) a timber bearing tool marks on the surface, (*B*) various timbers with cogging joints. (*C*) 3D model of the well lining set-up using laser images. (*D*) Sketch of the base frame with wedged tusk tenon joints and the frame with interlocked corner joints.

**Figure 5:**
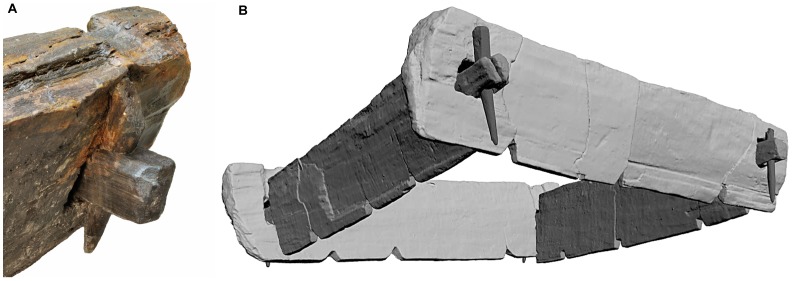
Basal frame construction of well A. (*A*) Wedged tusk tenon joint. (*B*) 3D laser rendering of the basal frame.

Two types of well linings were assembled into construction pits reaching the ground water level up to 7 m below the surface: a chest-like well lining (using timber logs) and a tube-like well lining (using hollowed trunk sections). The chest-like construction in well B served to stabilize the construction pit before a hollowed trunk was inserted (Figure 1c). Well E2 experienced two stratigraphically distinct construction phases (Figure 1b and Text S1). The older lining consisted of a hollowed maple tree resting on four oak boards that were not fixed to one other (Figure S19). The more recent lining was built on top of the previous lining using only logs. All of the chest-like well linings were constructed using notched timbers that were either cogged or interlocked at their corner joints (Figure S17). The linings of wells A and E1 rested on basal frames that were constructed with mortise and tenon joints. The tenons of well A extended beyond the outer face of the joined timber and were perforated and keyed by wooden wedges (Figure 4, Figure 5).

**Figure 6:**
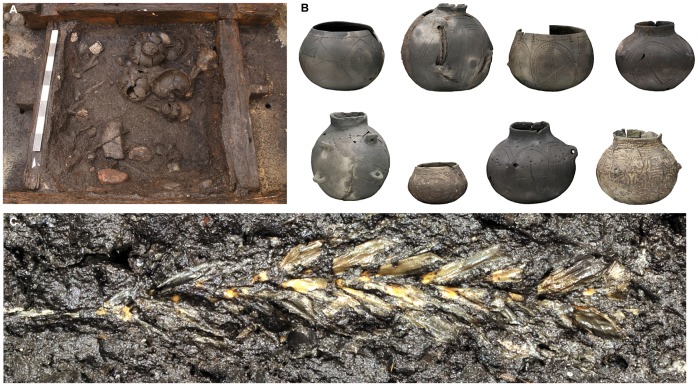
Finds from the fill of well A. (*A*) Well A during excavation. Within the square wooden lining, a dense deposition of pottery consisting of intact and broken vessels has been uncovered. (*B*) Selection of intact and restored pots representative of the ceramic spectrum of the LBK, consisting of jars, necked vessels and bowls (to scale, photorealistic renderings of laser scans). (*C*) Complete ear of Einkorn (*Triticum monococcum*, 70 mm in length) [27].

Well A was discovered at the margin of an LBK settlement of nearly 100 typical longhouses and a cemetery of approximately two dozen graves (Figure S1). From the exceptionally well-preserved wooden well lining, a subset of 134 timbers (≥20 tree-rings) was selected for dendrochronological analysis. We dated 47 timbers from the log construction, 72 from the construction pit, and five wooden remains from the internal deposits. All of the wood material from the log construction originated from only 13 individual oaks with trunk diameters of ∼0.8 to 1.0 m, which were harvested in 5102 BC (Figure S8). The individual trees were both radially and tangentially split into well-shaped beams (Figure 4b, and Text S1). A small plank from the construction pit was dated to 5099 BC and thus defines the initial construction onset. A small board from the internal backfill (5087±10 BC) would suggest a very short well lifespan (Text S1). This finding is independently confirmed by the typologically homogeneous LBK pottery from the fill (Figure 6). A reused board, however, can also not be ruled out.

However, re-used timbers excavated from the surrounding pit were more than 100 years older than the well lining itself. This widespread dating evidence from well A implies a long settlement activity of at least four generations preceding the construction of the well. Our dendrochronological dating allows the determination of an ascertained time span for a particular early Neolithic settlement.

Rich botanical remains from the well fill provide insight into past environmental conditions and the early Neolithic diet. The staple food consisted of two types of hulled wheat, einkorn (*Triticum monococcum*) and emmer (*Triticum dicoccum*) (Figure 6c). Carbohydrates from cereals were complemented with proteins from legumes, such as peas (*Pisum sativum*) and lentils (*Lens culinaris*). Oils were obtained from linseed (*Linum usitatissimum*) and poppy (*Papaver somniferum*). Wild fruits supplemented the diet, and included strawberries, sloe, apples, raspberries and hazelnuts. Two plants that have been considered archaeophytes in Central Europe were found in abundance: the bladder cherry (*Physalis alkekengi*) and the black henbane (*Hyoscyamus niger*). Henbane is a strong hallucinogenic drug and potential medicine. Its utilization as a medicinal plant or ritual drug has been suggested elsewhere [33].

The lower part of well A, which was filled with sediment after its abandonment, contained over 25 complete LBK vessels (Figure 6) as well as bone, stone and flint tools. The specific incised decoration style of the pottery finds corresponds to the younger phase of the LBK. In contrast to common belief, broken pottery was not typically discarded but it was instead repaired with birch tar and used in this state before final abandonment. Two vessels were completely redecorated after repair. They were covered outside with a thin layer of birch tar with intricate patterns made of cut-out strips of birch bark that were pasted on. This style of decoration was hitherto unknown for the LBK and bears no relation to the originally incised ornament underneath.

Many of the tool marks on the timber surfaces can be attributed to typical early Neolithic ground stone adzes. Unlike later archaeological cultures, parallel-hafted axes were unknown in the LBK. The predominant tool for woodworking was the transversely hafted adze with the ground stone blades that are extensively known from the archaeological record [34]. The observed tool marks prove the use of wider stone adzes (cutting edge width ∼50 mm) for finishing timber surfaces, whereas narrow stone adzes (‘shoe-last’ adzes, cutting edge width ∼20 mm) were employed for timber trimming (Figure 4a). This differentiated use of specialized tools for specific tasks is another indication of the high level of specialization in woodworking techniques. Nevertheless, it is unclear how and by which tools corner joints were notched, although the tool marks suggest the utilization of bone chisels.

The Central European Neolithization coincided with the Holocene Climate Optimum that occurred ∼7,500 years ago [2]–[4] (Figure S21). Relatively mild and humid conditions along with little variation in the Earth's climate system likely positively affected ecosystem productivity. Thus this may have also enabled the agricultural success of the first farmers, which was closely related to forest clearing and timber exploitation [8], [14]. The occurrence of larvae galleries of the thermophilic great capricorn beetle (*Cerambyx cerdo* L.) in 27% of the analyzed well timbers (Figure S7) provides additional independent evidence of a favorable climate during this time.

## Conclusions

This study demonstrates that the first farmers were also the first carpenters, contradicting the common belief that the invention of metal woodworking tools more than a thousand years later was imperative for complex timber constructions. Settlers of the early Neolithic time were able to build sophisticated corner joints and log constructions, which fulfilled all of the static requirements of massive water well linings. Their technical skills further imply the existence of complex constructions for LBK longhouse architecture [35]. Our results emphasize that water wells constitute a unique palaeoenvironmental archive for the overall data-sparse period of the early to mid Holocene. The archaeologically excavated and dendrochronologically dated wooden well constructions offer a holistic perspective on woodland use, resource utilization and woodworking techniques in addition to the vegetation and the climate conditions during the Central European Early Neolithic.

## Methods

Three wells A, E1, and E2 were block-lifted and excavated under optimal indoor conditions. No specific permits were required for the described field studies, as the archaeological excavation was carried out by the responsible governmental agency, the Archaeological Heritage Office Saxony in Dresden. The bottom four meters of well A was completely encased along with the backfilled construction pit. Finally, a bloc of a 70-ton encasement was recovered (Text S1, Figure S2, Figure S3). The excavation of well A was digitally recorded with millimeter accuracy using a reflectorless total station in combination with photogrammetry. Every timber, wooden find, and artifact was three-dimensionally recorded in situ and after removal and cleaning laser-scanned using a Minolta VI-910 (Figure 4, Figure 5). Each individual timber was documented at a precision of <0.8 mm, sufficient to record the smallest tool marks on the surfaces. A multi-object digital model of the wooden lining and its contents was constructed using the GeoMagic and AutoDesk 3dsMax software packages (Figure 4, Figure 5, Figure S4). All of the sediment from the well fill was wet-sieved to retrieve environmental and archaeological remains. Additional samples were taken for pedological, palynological, and micromorphological analyses. Next, 2–3 cm-thick samples were sawn from each timber (Text S1, Figure S5). To determine the number of timbers gained from one tree, all cross-sections were drawn to scale, indicating the pith, the sapwood, the waney edge and the course of the tree-rings and the medullary rays (Text S1, Figure S9). The ring widths were measured at a precision of 0.01 mm using a stereomicroscope, a measuring system and the PAST4 software by SCIEM (Scientific Engineering and Manufacture, Vienna). The tree-ring width data used for this study are included in (Data S1).

All of the dendrochronological parameters, including the pith, the waney edge, the number of tree-rings, the sapwood proportion and the wood anatomical features, were recorded (Table S2). A total of 151 tree-ring width series were cross-dated, and their arithmetic mean was calculated. This new master chronology was absolutely dated against the subfossil oak reference chronology from the Main River Valley [36] (Figure S13, Table S1, Text S1).

## Supporting Information

Text S1 **Supporting Information.**
(PDF)

Figure S1 **Archaeological plan of the LBK settlement from Altscherbitz with the located water well, nearly 100 typical longhouses and a cemetery of about two dozen graves.**
(PDF)

Figure S2 **70-ton block with the Altscherbitz well encased.**
(PDF)

Figure S3 **Indoor excavation of the Altscherbitz well.**
(PDF)

Figure S4 **3D laser rendering of the Altscherbitz basal frame.**
(PDF)

Figure S5 **Timber from the Altscherbitz well lining and sawn cross section sample.**
(PDF)

Figure S6 **Close-up view of a cross section (at 16× magnification, Altscherbitz timber 31–155).** The last two or three heartwood rings are discolored.
(PDF)

Figure S7 **Great capricorn beetle galleries (** ***Cerambyx cerdo*** **L.).**
(PDF)

Figure S8 **42 tree-ring series from split timbers from the Altscherbitz well lining can be attributed to one individual tree because of their similarity.**
(PDF)

Figure S9 **Split timbers from the construction pit can be attributed to one individual tree trunk.**
(PDF)

Figure S10 **37 trees reconstructed from 147 Altscherbitz timber tree-ring series.** Sapwood: blackened; waney edge: red; pith: black dot.
(PDF)

Figure S11 **Relationship between average growth rate (AGR) and mean segment length (MSL) of the Altscherbitz dataset.**
(PDF)

Figure S12 **Smoothed regional curves representing the average age trend of recent oaks from Central Eastern Germany (green) and Early Neolithic oaks from the Altscherbitz well construction (red).**
(PDF)

Figure S13 **Synchronization of the 124 Altscherbitz tree-ring series.** (*A*) EPS over 50 years, lagged by 25 years, (*B*) replication, (*C*) individual tree-ring series (black) in overlap with mean (red), (*D*) mean chronology in overlap with the chronology from the Main river valley after 10-year smoothing.
(PDF)

Figure S14 **Well from Brodau in the course of excavation with a piglet in the construction pit.**
(PDF)

Figure S15 **Highly decomposed oak timber from the Brodau well.**
(PDF)

Figure S16 (*A*) Brodau tree-ring series in overlap. (*B*) Brodau mean chronology (red) in overlap with the Altscherbitz reference chronology (blue).
(PDF)

Figure S17 **Joining techniques of early Neolithic well constructions.**
(PDF)

Figure S18 (*A*) 18 tree-ring series from Eythra well E1 in overlap. (*B*) Mean chronology from E1 (red) in overlap with the Altscherbitz reference chronology (blue).
(PDF)

Figure S19 **Eythra well E2: sketch of timber remains from structures 21 and 22.**
(PDF)

Figure S20 (*A*) Tree-ring series from the Eythra well E2 in overlap (grey). (*B*) The mean chronology (red) of E2 dated against the Altscherbitz mean (blue) chronology.
(PDF)

Figure S21 **Environmental change in the Early Neolithic.** (*A*) Pollen-based European temperature reconstruction, (*B*) subfossil-based Alpine treeline reconstruction, (*C*) temporal distribution of glacial 95 wood remains, and (*D*) peat bog-based hydroclimatic reconstruction from the UK.
(PDF)

Table S1 **Grid report of the correlation results between chronologies from well A, B, E1, E2 and the Main river valley.** TBP = t-value after Baillie and Pilcher, THO = t-value after Hollstein, Gl = % of Gleichläufigkeit, r = correlation coefficient.
(PDF)

Table S2 **Tree-ring inventory.**
(PDF)

Data S1 **Tree-ring width data (Tucson Format).**
(PDF)
