# Supplementary material for: Early Neolithic Water Wells Reveal the World's Oldest Wood Architecture
Source: PLoS One. 2012 Dec 19;7(12):e51374. doi: 10.1371/journal.pone.0051374 (PMC3526582; doi:10.1371/journal.pone.0051374)
Supplement: Text S1 — Supporting Information. (PDF) [file pone.0051374.s001.pdf]

Supporting Information

Early Neolithic water wells reveal the world's oldest wood architecture

Willy Tegel, Rengert Elburg, Dietrich Hakelberg, Harald Stäuble & Ulf Büntgen

Text S1

Archaeological Excavation. Wells from the early Neolithic Linear Pottery Culture (LBK) are very rare archaeological finds, numbering only about two dozen. Most of the wooden linings have decayed due to fluctuations in the groundwater level, precluding extensive archaeological and dendrochronological studies. In this respect, the wells recently found in eastern Germany offer a unique opportunity to explore prehistory. All four of the wells presented here were found during large-scale development-led archaeological excavations carried out by the Archaeological Heritage Office Saxony. An archaeological study of a fifth Neolithic well from Leipzig-Plaussig has not yet been completed; therefore, it will not be considered here.

Two wells from Eythra (E1 and E2) were found in the second half of the 1990s in the lignite mining area of Zwenkau south of Leipzig within an extensive early Neolithic settlement. Both wells were predominantly excavated on the spot, and only the lowermost parts were block lifted for further excavation in the workshops of the Archaeological Heritage Office Saxony. The well from Brodau (B) was discovered in 2005 on the edge of an LBK settlement excavated in connection with the construction of a large water channel related to lignite mining near Delitzsch to the north of Leipzig. This complex was completely excavated in the field due to the inferior preservation of the wooden lining. Finally, the well from Altscherbitz (A) was found during extensive excavations of another large LBK settlement prior to the expansion of the international airport of Halle/Leipzig on the northwestern outskirts of Leipzig (Figure S1). This well, which is over seven meters deep, was substantially deeper than those previously found and, therefore, much better preserved below groundwater level. Because there was no time to adequately excavate the well immediately, the lower half of the construction was block lifted with all

waterlogged organic material. This operation resulted in a block weighing over 70 tons, which was transported to the Archaeological Heritage Office Saxony in Dresden (Figure S2). After a storage period, the excavation was implemented indoors for 28 months, from the beginning of 2008 until the summer of 2010 (Figure S3).

The fill of all of the structures, especially well A, yielded a wide spectrum of organic finds and an unsurpassed number of artefacts and environmental remains. Due to the near-perfect conservation of the timber surfaces, these findings could be recorded using the high-resolution 3D laser scanner. The resulting digital models show in fine detail the traces left by the stone woodworking tools used to cut and shape the timber (Figures S3 and S4). The tool marks indicate the use of two different types of stone adzes: a flat, broad adze for smoothing the surface and a narrow, high-domed adze for trimming the timbers.

Dendrochronological Studies. A total of 167 oak (*Quercus* spp.) waterlogged timbers from four LBK water wells were dendrochronologically analyzed. A total of 151 could be dated (Table S2). The potential oak species, *Q. robur* L. and *Q. petraea* (Matt.) Liebl., in central Germany cannot be distinguished anatomically [1]. For the analysis, only timbers with more than 10 tree-rings were selected. These timbers originate from the log constructions, the construction pits and the internal fills of the well shafts. To measure the tree-ring series, cross-sections were cut from the individual timbers (Figure S5). Samples were taken in the section of the timbers with the most tree-rings and preferably featuring waney edge and pith. Zones displaying disturbances of the tree-ring growth, e.g., through knots or insect galleries, were avoided. Surfaces were created using razorblades to prepare the tree-ring series for microscopic analysis. The tree-ring widths were measured to an accuracy of 1/100 mm using a stereo microscope and a measuring system.

The Well from Altscherbitz (A). The timbers from the Altscherbitz well were perfectly preserved. The oak heartwood was blackened through the reaction of iron compounds in the sediment with tannin in the wood. Oak sapwood does not contain tannin and had a bright brown color. However, the more decomposed heartwood zones possess a similar bright color comparable to sapwood (Figure S6).

Although the cellulose had largely decomposed, the well-preserved lignin structure had ensured the solidity of the timber in most places. Galleries as large as 25 mm wide were evident in the heartwood of approximately 40 samples (Figure S7). The galleries originate from the larvae of the great Capricorn beetle (*Cerambyx cerdo L.*). This thermophile species is rare today

and is considered an ecological relic from primeval forests, preferring mature homogeneous oak stands.

The tree-ring series of individual timbers were grouped according to similarities in high- and low-frequency growth variances to identify the individual trees (Figure S8). Groupings were performed by visual comparison of the growth patterns and correlation calculations. The cross-section shapes, the pith, the sapwood and the waney edge were also considered (Figure S9). It was not always possible to determine unequivocally which timbers have been taken from a single tree. Trees growing under similar conditions and in close proximity may develop very similar tree-ring patterns. Moreover, tree-ring patterns may vary considerably throughout individual trees depending on the individual geotropic and phototrophic reactions.

The groupings indicated that at least 37 mature individual trees (70–100 cm in diameter) were used in the construction of the well lining. The oldest oak tree used was approximately 270 years old (Figure S10). Pith, sapwood and waney edges were still present in 9, 37 and 15 timbers, respectively.

The mean segment length (MSL) was 119 years at an average growth rate (AGR) of 1.4 mm p.a. (Figure S11). The assessment of the MSL and the AGR of the raw measurement series indicates that shorter oak series (< 50 years) with a greater fraction of juvenile trees tended to grow more rapidly, whereas the longer series of more mature trees revealed generally slower growth. From a tree age of ~ 50 years onward, the annual growth rate remains nearly constant until old age. This behavior is indicative of a stand with a closed canopy at a very early stage.

To determine the density of the stand present at that time, the growth rates and age trends were compared, consulting a tree-ring series from 250 recent oak samples from central eastern Germany. Recent oaks from thinned stands in that region display a clearly higher AGR of 2 mm (Figure S12). Lower growth rates, as represented by the Altscherbitz dataset, can be explained by a lack of nutrients, light and water.

The average age trend observed is considerably lower than that of the recent trees (Figure S13). The early Neolithic trees germinated in a fairly closed stand and were undoubtedly not coppices growing in a clearance. Clearing effects, which result in abrupt growth release, are not visible in the tree-ring patterns. This finding suggests that the trees used grew for a long time in dense primeval forest stands.

A total of 49 oak timbers from the log construction, 76 from the construction pit and nine from the internal fill were dendrochronologically analyzed (Table S2). A total of 124 annually resolved

tree-ring width series could be compiled to cover 328 years continuously from 5426–5099 BC (Figure S13b and c). The tree-ring patterns were very similar, as reflected by the good interseries correlation (*Rbar* = 0.4). The expressed population signal values (*EPS* over 50 years, lagged by 25 years) consistently range above the commonly applied threshold of 0.85 (Figure S13a). Thus, all of the timbers employed likely originate from the same woodland.

This tree-ring chronology is of particular importance because it could be cross-dated with absolute certainty against the oak chronology from the Main River Valley (based exclusively on subfossil oaks) approximately 200 km southwest of the archaeological sites (Figure S13d) [2]. These chronologies (after 10-year smoothing, > 5 series each) correlate at $r = 0.51$ over their common period between 5381 and 5012 BC. This new regional oak chronology enabled the dating of the mean chronologies from the Eythra and Brodau LBK wells, whose replication was until then insufficient to present a typical regional tree-ring pattern. The systematic dendrochronological study of all of the timbers from the Altscherbitz LBK well currently provides the basis for an early Neolithic oak chronology for Central Eastern Germany.

Dendrochronological dating may produce results with varying precision. In an ideal situation, a precise annual determination of the felling date would be possible. A prerequisite for this determination is the presence of the waney edge in the sample, i.e., the last tree-ring formed with the curved cambial surface exposed.

Oak samples, particularly those featuring the remains of sapwood rings, enable sapwood dating by the statistical approximation of the number of missing rings before the waney edge. As a general rule, oak trees develop between 10 and 30 sapwood rings. According to this theory, at 20 ± 10 years for the sapwood, based on the last heartwood ring, the felling date can be estimated with a precision of ± 10 years. If the sapwood is completely absent, 10 sapwood rings can be added to the last existing heartwood ring. In this way, a *terminus post quem* can be established for the felling date.

For six of the Altscherbitz timbers, the waney edge was still present. These trees were cut in 5117, 5114, 5111, 5102 and 5099 BC. The trees for the beams from the well lining itself were cut in 5102 BC. Smaller timbers also featuring waney edges were later used for wedging the seams of the log construction and delivered felling dates before and after 5102 BC. For these patches, 18-year-old recycled and freshly felled timbers were used. The youngest felling date for this wood is 5099 BC. This finding suggests that the trees employed for the well lining were felled at least three years before the construction was completed. A smaller board from the internal

backfill of the well was dated at 5087 BC ± 10 years using sapwood dating. This date indicates that the well was only used for a brief time.

Recycled oak boards from the construction pit provide information about the duration of the Altscherbitz LBK settlement. The oak boards are approximately 100 years older (5201 BC ± 10 years) and clearly originate from a construction above ground level: Longhorn beetles, which do not live in humid environments and whose larvae feed on dead wood, left traces on the timber surfaces.

The Well from Brodau (B). The wooden lining from Brodau likely served as an encasing of the construction pit. The split oak timbers measuring between 1.2 and 1.6 m did not form a closed wooden frame and overlapped in an irregular way. Only some of the timbers had notches at their ends. It is possible that they helped prevent the fine sand layers below glacial loams from shifting while excavating the construction pit. The well lining itself consisted of a hollowed tree trunk (0.75-0.8 m in diameter).

The remains of two piglets were discovered at the margins of the construction pit (Figure S14). These remains could possibly be interpreted as ritual offerings in the course of construction, regardless of the probable pollution of the water.

Generally, the wood preservation was very poor (Figure S15). Wood was completely decomposed in some places, and the outermost zones of the timbers were badly crushed. The more resistant medullary rays were better preserved. Woodworking and tool marks were not visible on the wood surface. It was impossible to determine the species of the hollowed tree trunk because of its poor preservation, which is probably due to the groundwater lowering during the opencast lignite mining, enabling the access of aerial oxygen and thus an acceleration of bacterial wood decay.

Six timbers from the Brodau well lining were suitable for dendrochronological analysis. The two other recovered timbers were so decomposed that it was not possible to measure the tree-ring series. From the tree-ring series of the six measured samples, a Brodau mean chronology was established. The 259-year-long chronology could be synchronized with reference chronologies for the period between 5468 and 5210 BC (Figure S16). Strong correlation values with the Altscherbitz mean chronology were able to confirm this chronological position (Table S1).

All six oak timbers from the Brodau well lining could be dated. In the case of one badly decomposed and crushed timber, the sapwood was still present. For this sample, the tree-rings could not be counted or measured. However, based on the boundary between the heartwood and

the sapwood, the tree used was cut down in 5190 BC ± 10 years. Another timber lacked sapwood but displayed an almost identical tree-ring pattern, suggesting that both timbers were cleft from the same tree trunk. In this case, the timber must also have had the same felling date. As the remaining four Brodau timbers lack sapwood, a *terminus post quem* for the felling can only be obtained from the tree-ring series (after 5219, 5235, 5237 and 5229 BC). By comparing the similarities based on growth trends and annual variances, the timbers can be attributed to five individual trees. The trees used were 200–300 years old.

Additionally, samples were also taken from the piglet remains for radiocarbon analysis. The results confirm the dendrochronological findings (radiocarbon years: MAMS-12434: 6263 ± 29 BP and MAMS-12435: 6252 ± 33 BP).

The Well from Eythra (E1). Eighteen radially split oak timbers from this square well lining (internal dimensions: c. 0.85 x 0.85 m) assembled as a log construction were analyzed. The well lining was uncovered in the lower part of the 4.5-m-deep construction pit, which had a maximum diameter of 4 m. The well lining survived from the bottom up to the fifth layer of the split timbers. Above this level, all of the wood had decayed. The length of the timbers varies between 1.3 and 1.9 m. All of the timbers have wedged cross-sections and fit in with the tapering edge at the pith downward. Notches were carved in these edges, and all of the timbers were joined at the corners by interlocking or notching to set up the square well lining of the log construction (Figure S17).

On the timber surfaces, extensive tool marks have survived that stem from the stone adzes, whose cutting edges were c. 5 cm wide. On the end grain surfaces at the extremities of the split timbers and in the notches, tool marks were no longer visible due to surface erosion, which was probably caused by trickling water and sediment flow.

The well lining rested on a square frame of four massive split timbers, each c. 1.30 m long, which were mortised at the corners. The square frame clearly served as a foundation for the well lining (Figure S17).

The tree-ring series from 18 timbers could be synchronized. Due the similarity of the tree-ring patterns, all of the timbers were radially split from one tree trunk. The oak tree used was approximately 120 years old. Using correlation calculations and a visual comparison to the absolutely dated Altscherbitz mean chronology, the Eythra mean chronology could be dated to 5212–5098 BC (Figure S18 and Table S1). The oak tree employed for the Eythra E1 well was cut in 5098 BC. There is no dendroarchaeological evidence indicating repairs or reused timbers.

The Well from Eythra (E2). From the well lining of Eythra E2 (structure 21), only a square chest-like structure made of four oak boards survived. The poorly preserved boards appeared to be joined at the corners by common halving with possibly fragmentary remains from the notches. The internal dimensions were 0.67 x 0.74 m. No further wooden remains could be detected in the soil above this chest-like structure. This lack may be due to poor preservation conditions, but it is also possible that the rising part of the well lining was dismantled in ancient times.

The timber surfaces bear clear traces of erosion. Tool marks are no longer recognizable. The notches appear to have been preferentially cut into knot zones. Tenuous galleries spread in places slightly below the split timber surface. It is likely that these boards are split timbers that were already in use above ground level (possibly in house constructions) and then reused in the construction of the well, as there seems to be no other reasonable explanation for the existence of the galleries below the split surfaces.

Half of a hollowed-out tree trunk (structure 22) was located slightly off-center below the box-like structure of structure 21. The remains of this tube well lining rested on four upright thin oak boards, which were not joined together and only supporting each other and pressed into the gravel (Figure S19). The end grain surfaces of the radially split, 3–4 cm-thick boards featured tool marks of a very narrow stone adze blade approximately 2 cm in width, known in archaeological terminology as ‘Schuhleistenkeil’. The adzes were used here to cut the boards to the required length. The tube itself was made from a hollowed maple tree that measured ~ 0.8 m in diameter with a surviving height of ~ 0.61 m and a wall thickness of c. 4 cm. The internal surface bore no remaining tool marks, whereas the outer surface was formed by a waney edge without any bark.

Only two timbers from structure 21 could be dendrochronologically analyzed. Both timbers lacked preserved sapwood. The measured tree-ring series could be synchronized and dated against the Altscherbitz mean chronology between 5375–5216 BC (Figure S20 and Table S1). By assuming at least 10 absent sapwood rings, the trees used must have been cut after 5206 BC (*terminus post quem*).

References

1. Feuillat F, Dupouey JL, Sciama D, Keller RA (1997) A new attempt at discrimination between *Quercus petraea* and *Quercus robur* based on wood anatomy. Can J For Res 27: 343–351.
2. Spurk M et al. (1998) Revisions and Extensions of the Hohenheim Oak and Pine Chronologies: New Evidence about the Timing of the Younger Dryas/Preboreal Transition. Radiocarbon 40: 1107-1116.
