# Supplementary material for: Early Neolithic Water Wells Reveal the World's Oldest Wood Architecture
Source: PLoS One. 2012 Dec 19;7(12):e51374. doi: 10.1371/journal.pone.0051374 (PMC3526582; doi:10.1371/journal.pone.0051374)
Supplement: Figure S1 — Archaeological plan of the LBK settlement from Altscherbitz with the located water well, nearly 100 typical longhouses and a cemetery of about two dozen graves. (PDF) [file pone.0051374.s002.pdf]

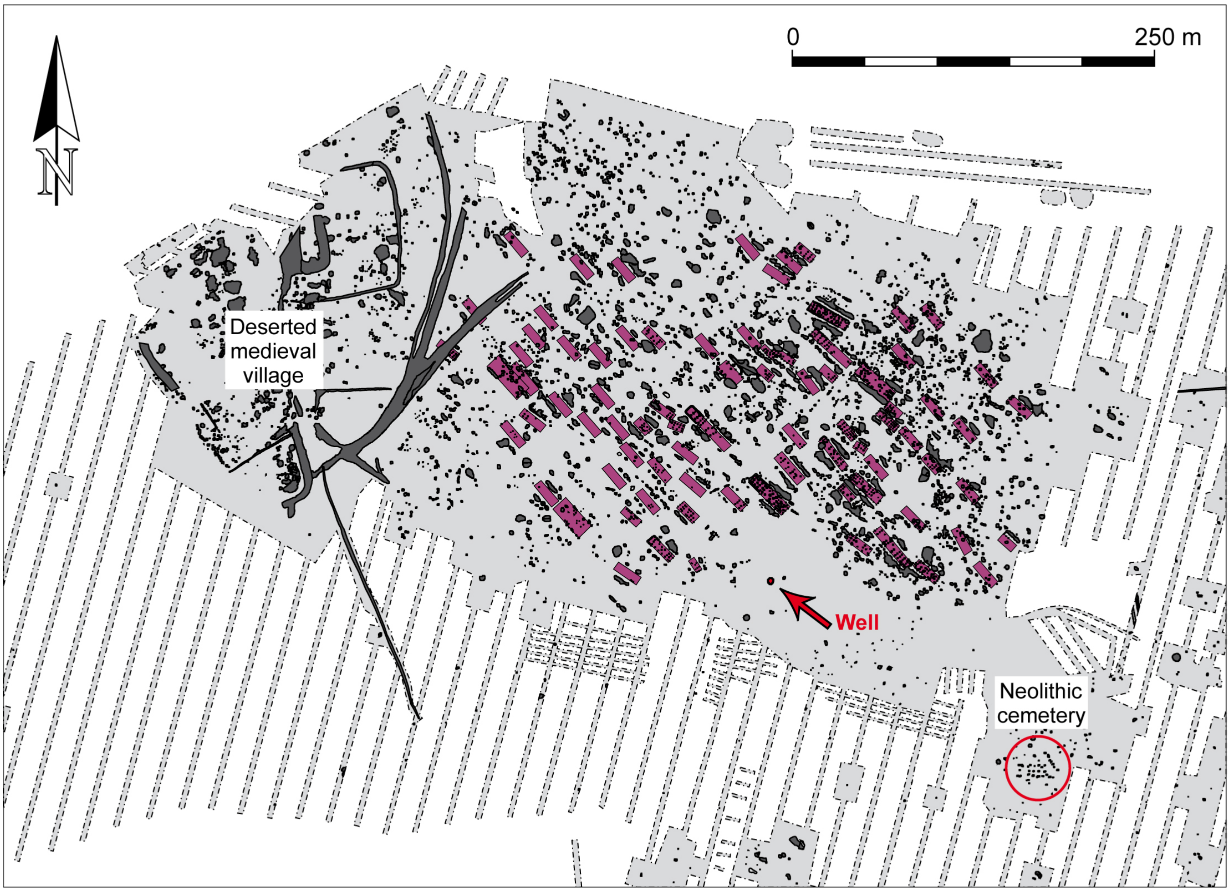

Figure S1. Archaeological plan of the LBK settlement from Altscherbitz with the located water well and nearly 100 typical longhouses and a cemetery of about two dozen graves.
