# Supplementary figures and images for: Early Neolithic Water Wells Reveal the World's Oldest Wood Architecture
Source: PLoS One. 2012 Dec 19;7(12):e51374. doi: 10.1371/journal.pone.0051374 (PMC3526582; doi:10.1371/journal.pone.0051374)

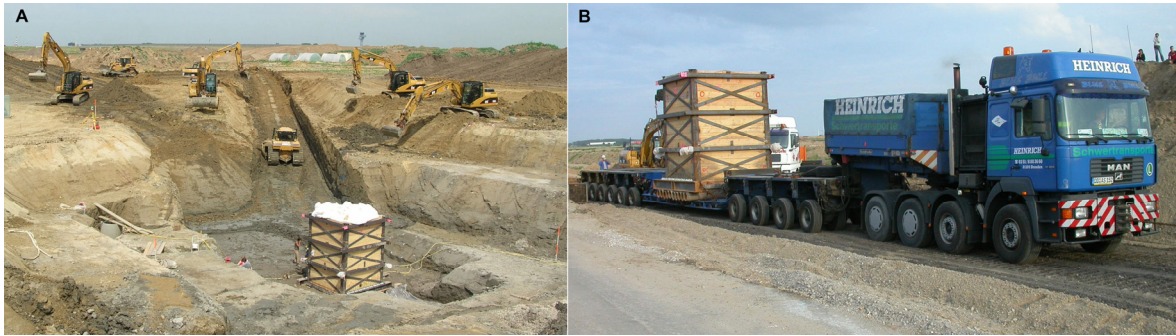

**Figure S2.** 70-ton block with the Altscherbitz well encased.

Supplement: Figure S2 — 70-ton block with the Altscherbitz well encased. (PDF) [file pone.0051374.s003.pdf]

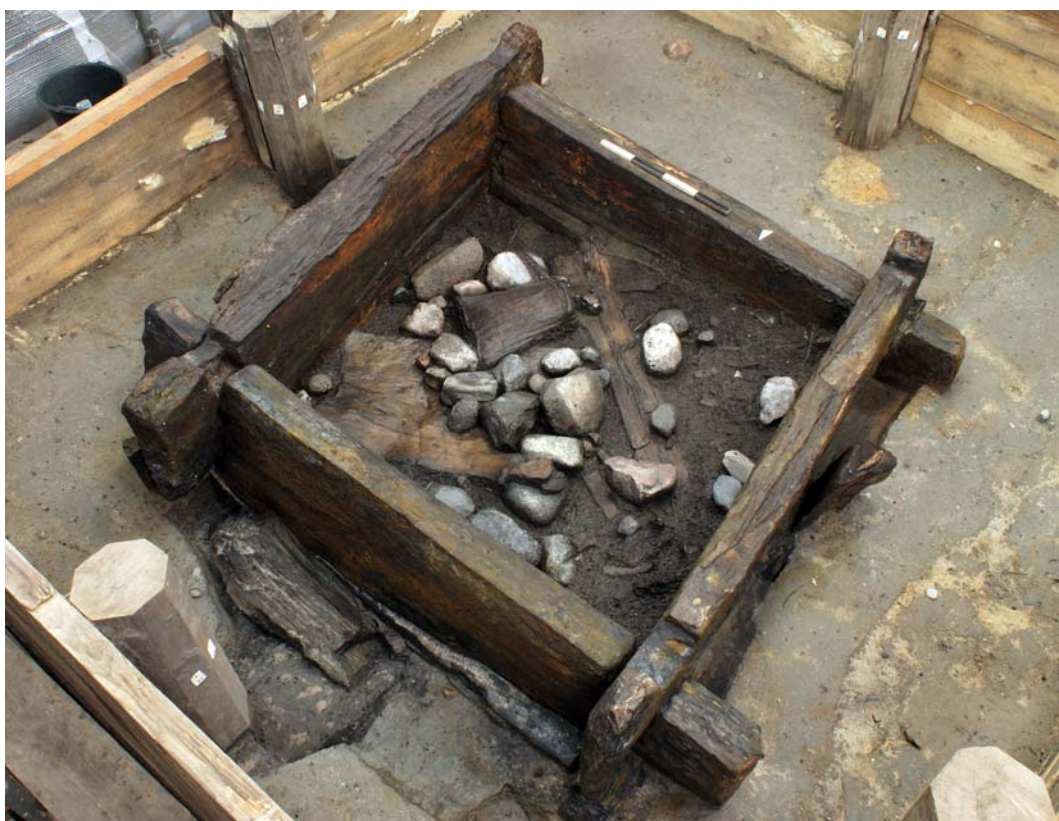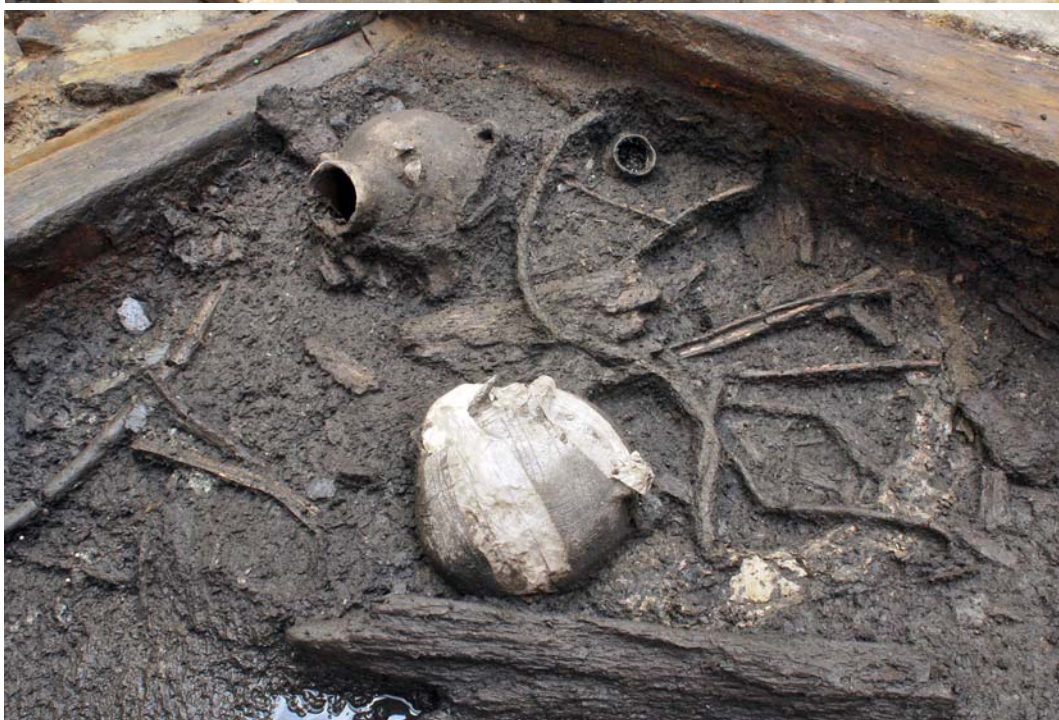

**Figure S3.** Indoor excavation of the Altscherbitz well.

Supplement: Figure S3 — Indoor excavation of the Altscherbitz well. (PDF) [file pone.0051374.s004.pdf]

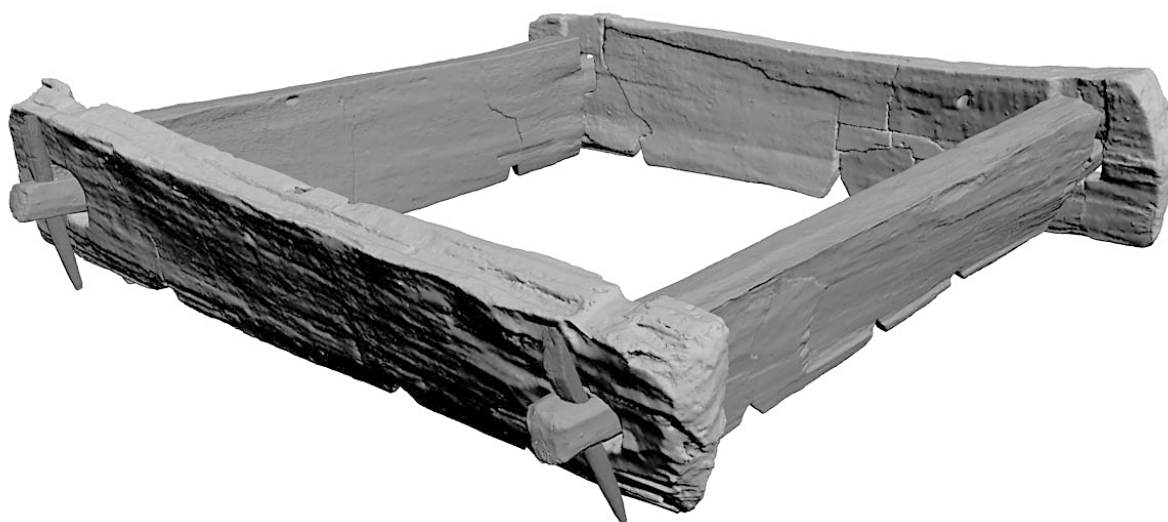

**Figure S4.** 3D laser rendering of the Altscherbitz basal frame.

Supplement: Figure S4 — 3D laser rendering of the Altscherbitz basal frame. (PDF) [file pone.0051374.s005.pdf]

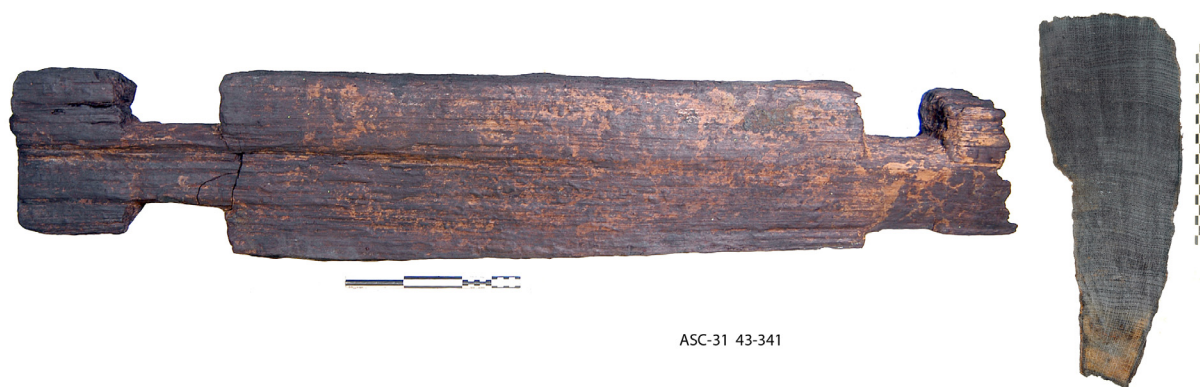

**Figure S5.** Timber from the Altscherbitz well lining and sawn cross section sample.

Supplement: Figure S5 — Timber from the Altscherbitz well lining and sawn cross section sample. (PDF) [file pone.0051374.s006.pdf]

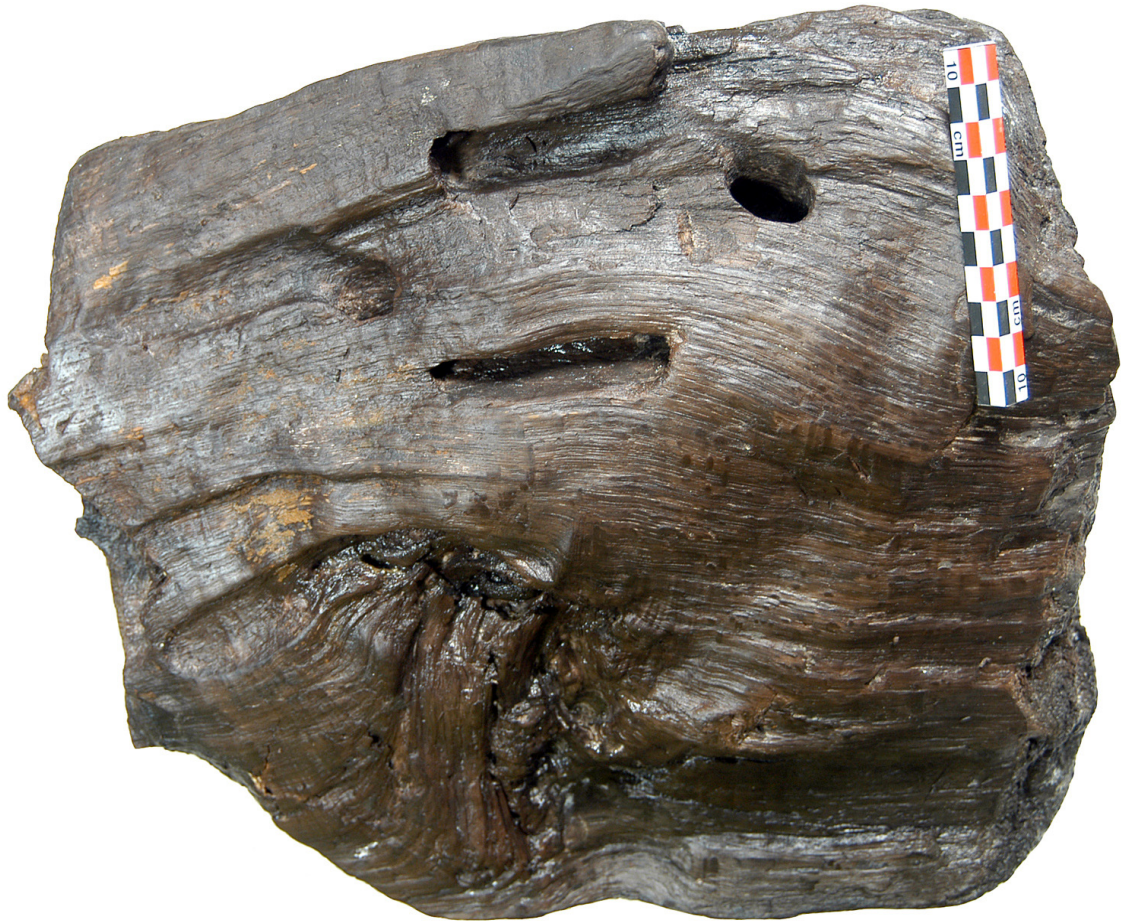

**Figure S7.** Great capricorn beetle galleries (*Cerambyx cerdo* L.).

Supplement: Figure S7 — Great capricorn beetle galleries ( Cerambyx cerdo L.). (PDF) [file pone.0051374.s008.pdf]

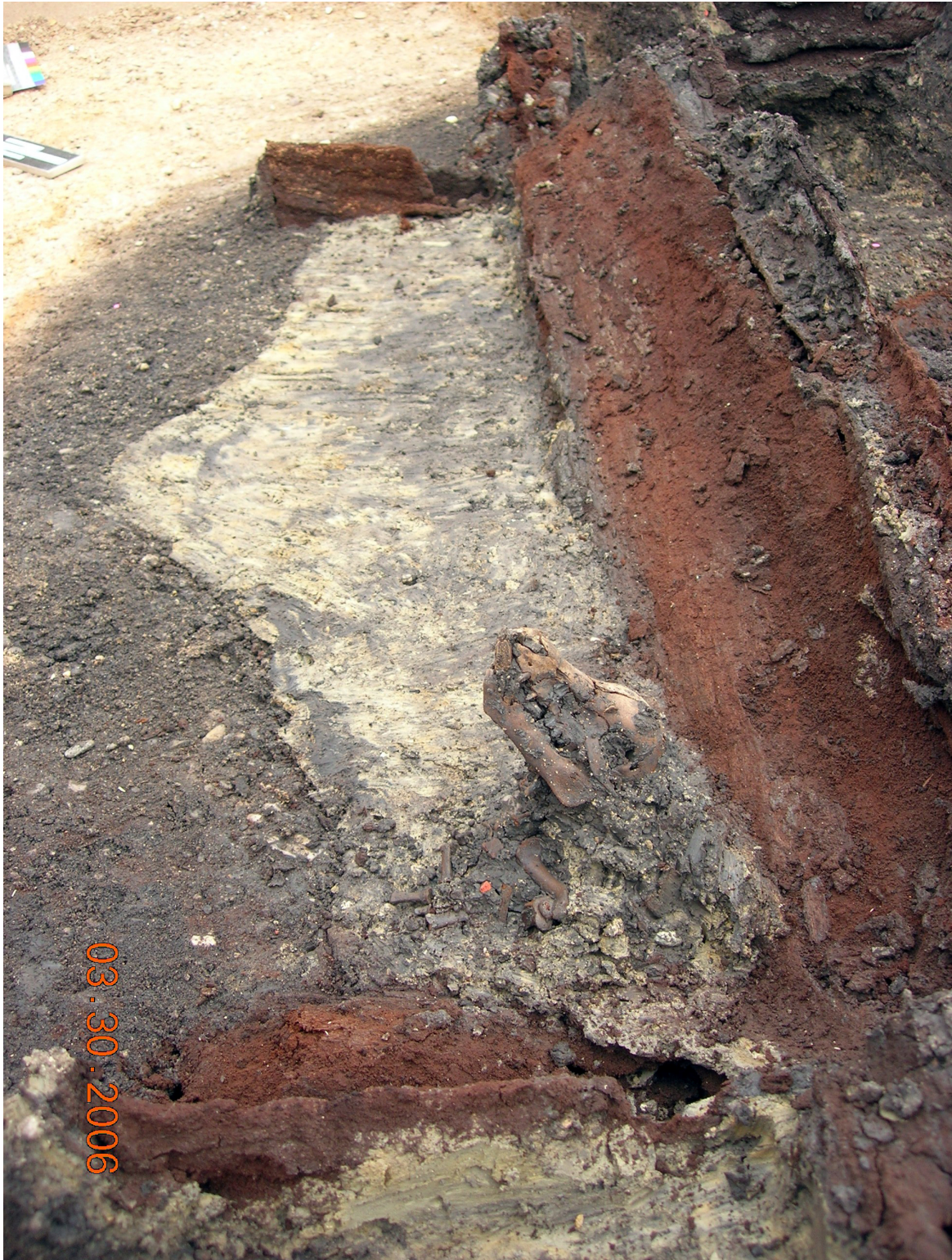

**Figure S14.** Well from Brodau in the course of excavation with a piglet in the construction pit.

Supplement: Figure S14 — Well from Brodau in the course of excavation with a piglet in the construction pit. (PDF) [file pone.0051374.s015.pdf]

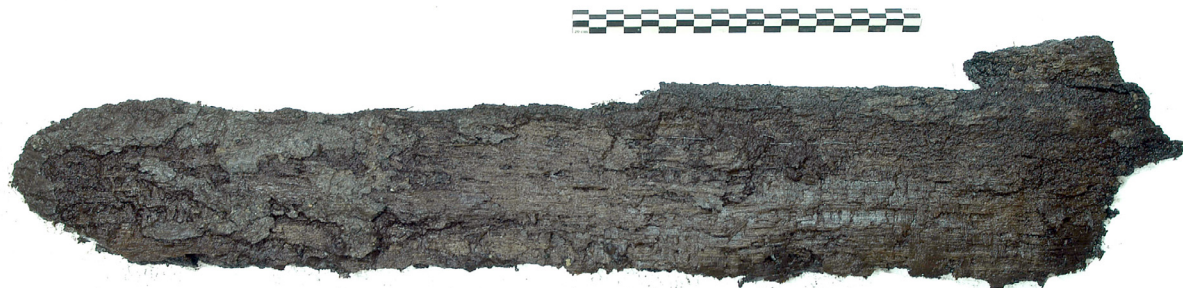

**Figure S15.** Highly decomposed oak timber from the Brodau well.

Supplement: Figure S15 — Highly decomposed oak timber from the Brodau well. (PDF) [file pone.0051374.s016.pdf]

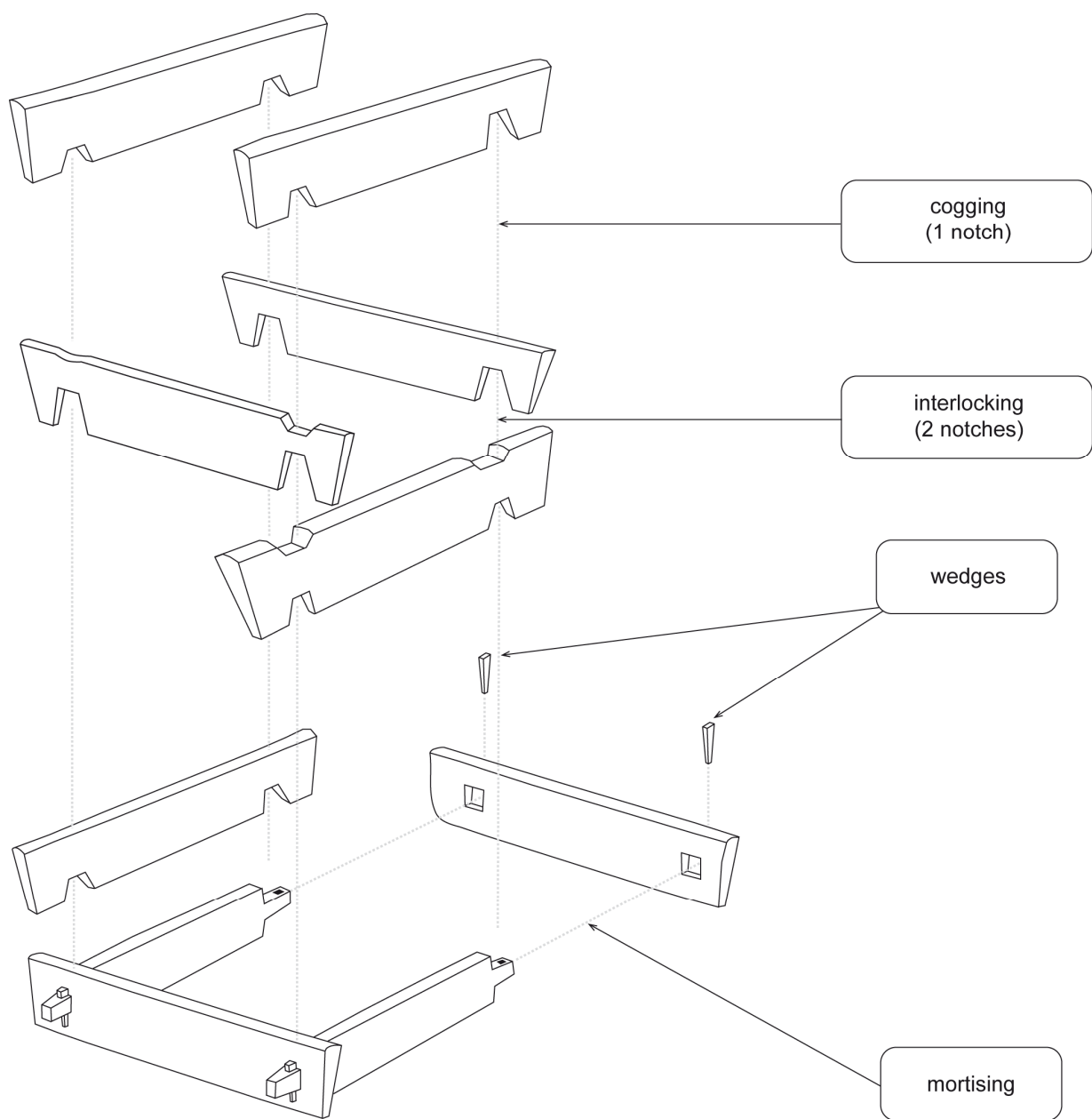

**Figure S17.** Joining techniques of early Neolithic well constructions (scheme by D. Hakelberg).

Supplement: Figure S17 — Joining techniques of early Neolithic well constructions. (PDF) [file pone.0051374.s018.pdf]

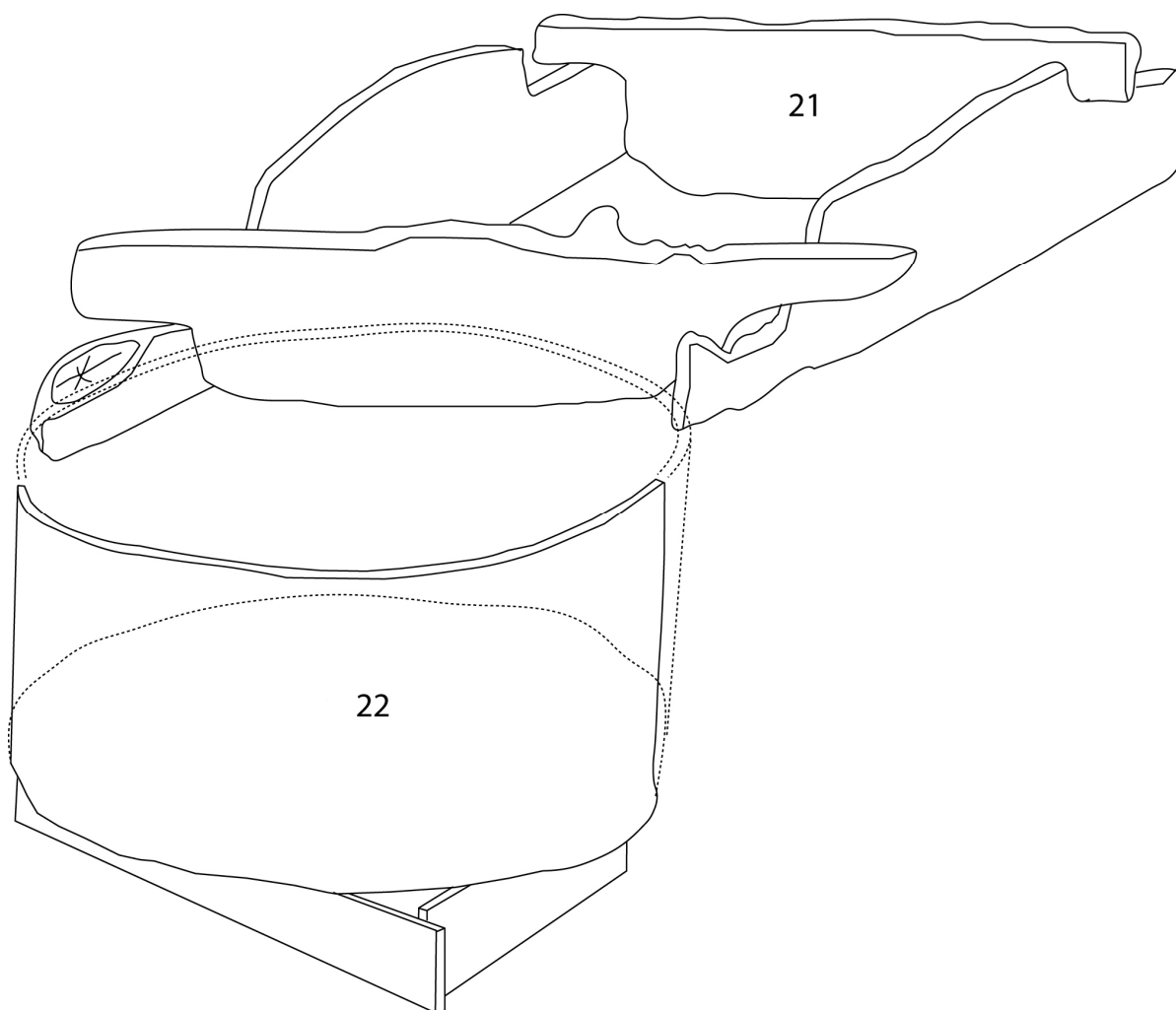

**Figure S19.** Eythra well E2: sketch of timber remains from structures 21 and 22.

Supplement: Figure S19 — Eythra well E2: sketch of timber remains from structures 21 and 22. (PDF) [file pone.0051374.s020.pdf]
