# Supplementary material for: Early Neolithic Water Wells Reveal the World's Oldest Wood Architecture
Source: PLoS One. 2012 Dec 19;7(12):e51374. doi: 10.1371/journal.pone.0051374 (PMC3526582; doi:10.1371/journal.pone.0051374)
Supplement: Figure S6 — Close-up view of a cross section (at 16× magnification, Altscherbitz timber 31–155). The last two or three heartwood rings are discolored. (PDF) [file pone.0051374.s007.pdf]

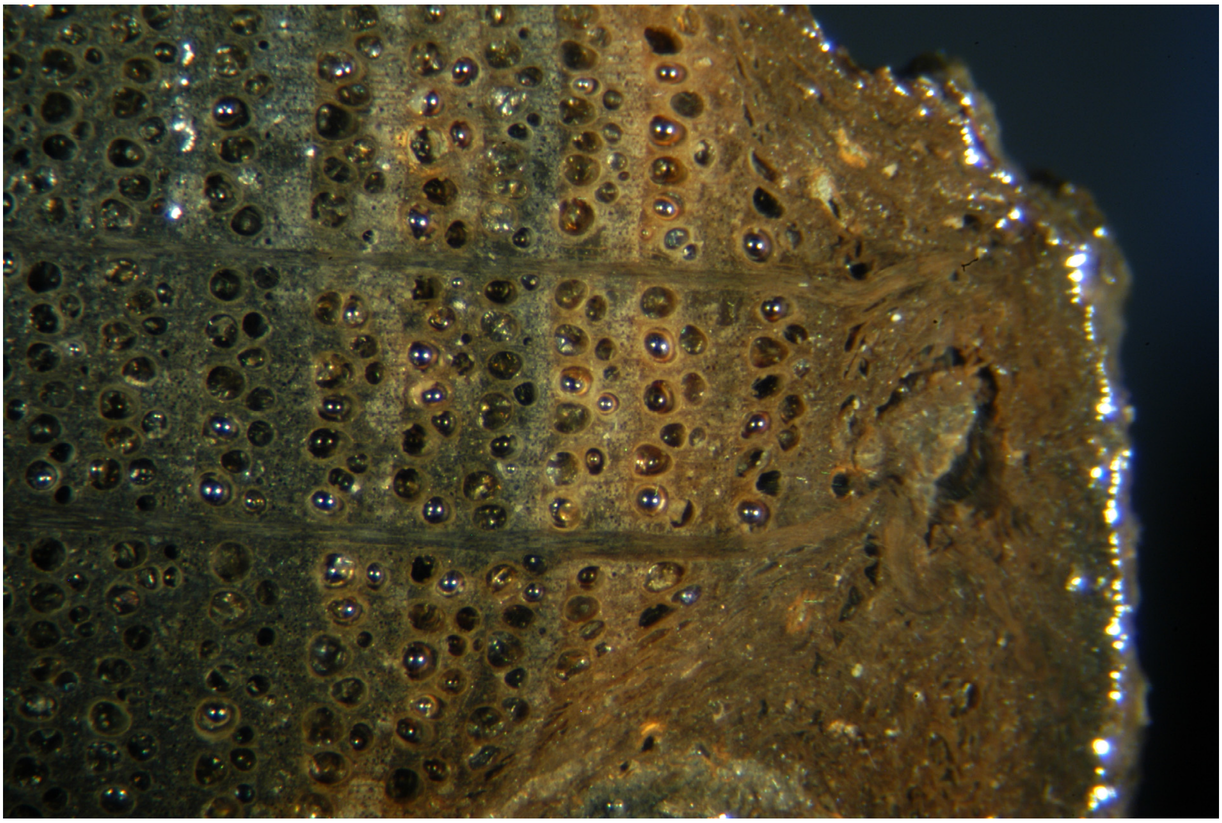

Figure S6. Close-up view of a cross section (at 16x magnification, Altscherbitz timber 31-155). The last two or three heartwood rings are discolored.
