# Supplementary material for: Early Neolithic Water Wells Reveal the World's Oldest Wood Architecture
Source: PLoS One. 2012 Dec 19;7(12):e51374. doi: 10.1371/journal.pone.0051374 (PMC3526582; doi:10.1371/journal.pone.0051374)
Supplement: Figure S8 — 42 tree-ring series from split timbers from the Altscherbitz well lining can be attributed to one individual tree because of their similarity. (PDF) [file pone.0051374.s009.pdf]

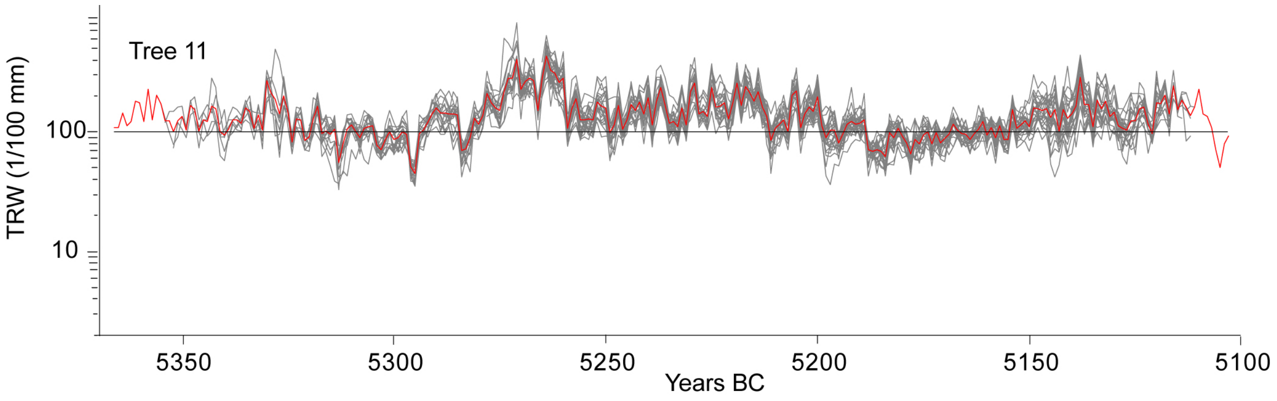

Figure S8. 42 tree-ring series from split timbers from the Altscherbitz well lining can be attributed to one individual tree (of a total of 13 trees used) because of their similarity.
