# Supplementary material for: Early Neolithic Water Wells Reveal the World's Oldest Wood Architecture
Source: PLoS One. 2012 Dec 19;7(12):e51374. doi: 10.1371/journal.pone.0051374 (PMC3526582; doi:10.1371/journal.pone.0051374)
Supplement: Figure S9 — Split timbers from the construction pit can be attributed to one individual tree trunk. (PDF) [file pone.0051374.s010.pdf]

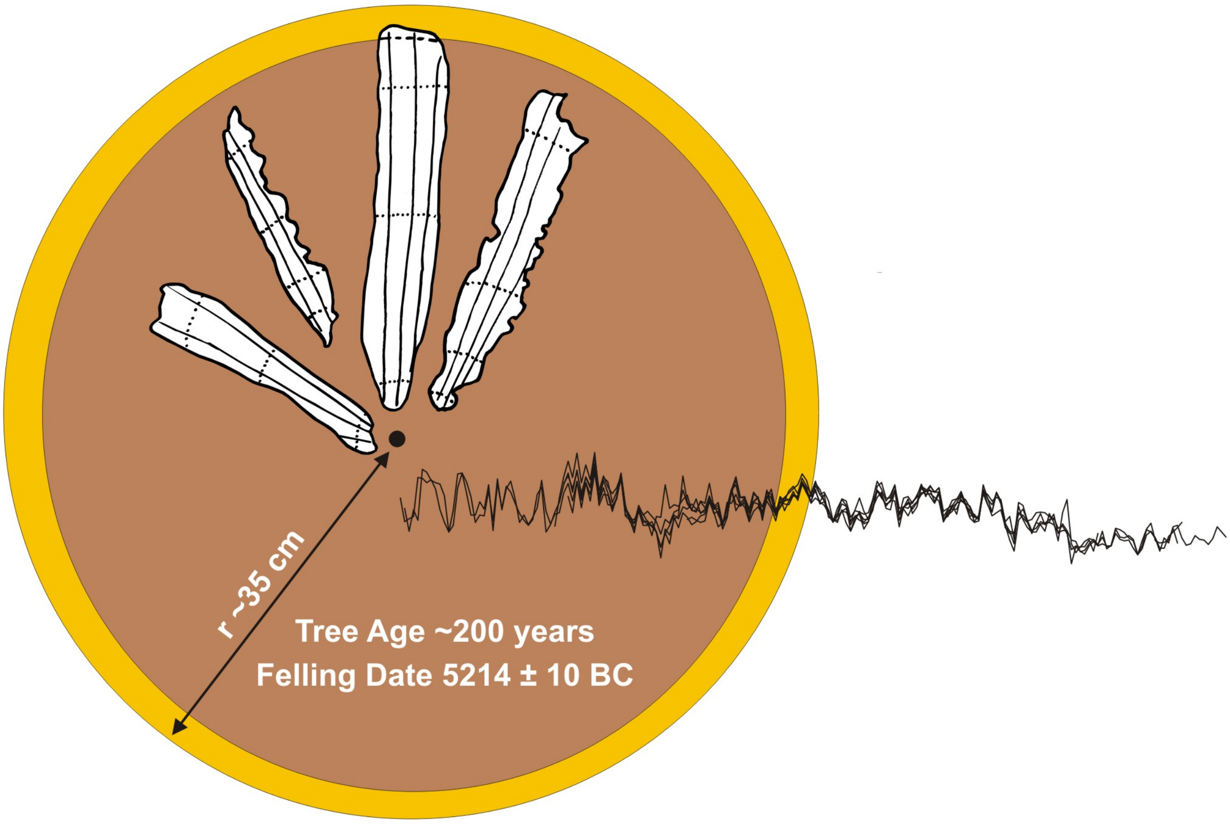

Figure S9. Split timbers from the construction pit can be attributed to one individual tree trunk.
