# Supplementary material for: Early Neolithic Water Wells Reveal the World's Oldest Wood Architecture
Source: PLoS One. 2012 Dec 19;7(12):e51374. doi: 10.1371/journal.pone.0051374 (PMC3526582; doi:10.1371/journal.pone.0051374)
Supplement: Figure S10 — 37 trees reconstructed from 147 Altscherbitz timber tree-ring series. Sapwood: blackened; waney edge: red; pith: black dot. (PDF) [file pone.0051374.s011.pdf]

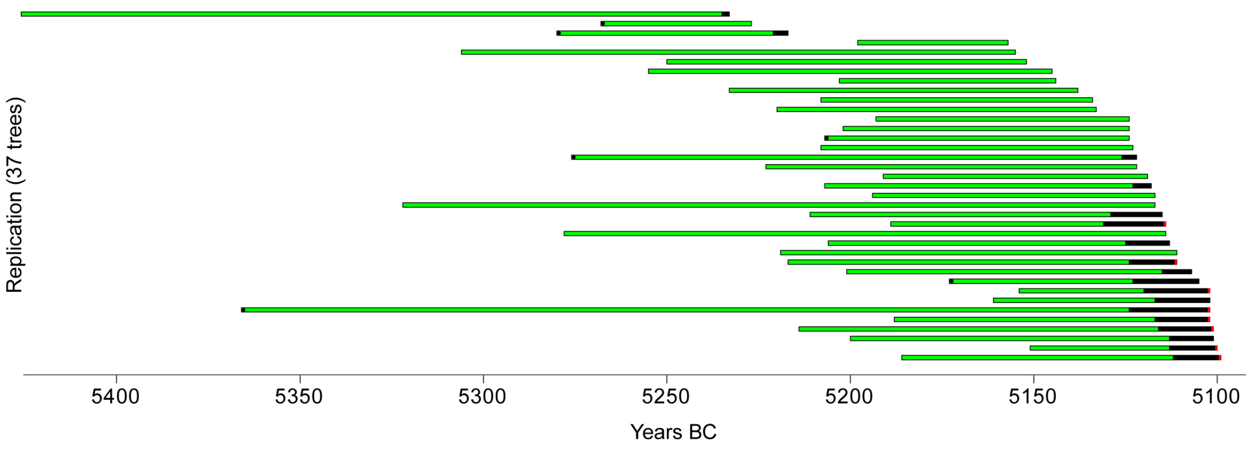

Figure S10. 37 trees reconstructed from 147 Altscherbitz timber tree-ring series. Sapwood: blackened; waney edge: red; pith: black dot.
