# Supplementary material for: Early Neolithic Water Wells Reveal the World's Oldest Wood Architecture
Source: PLoS One. 2012 Dec 19;7(12):e51374. doi: 10.1371/journal.pone.0051374 (PMC3526582; doi:10.1371/journal.pone.0051374)
Supplement: Figure S11 — Relationship between average growth rate (AGR) and mean segment length (MSL) of the Altscherbitz dataset. (PDF) [file pone.0051374.s012.pdf]

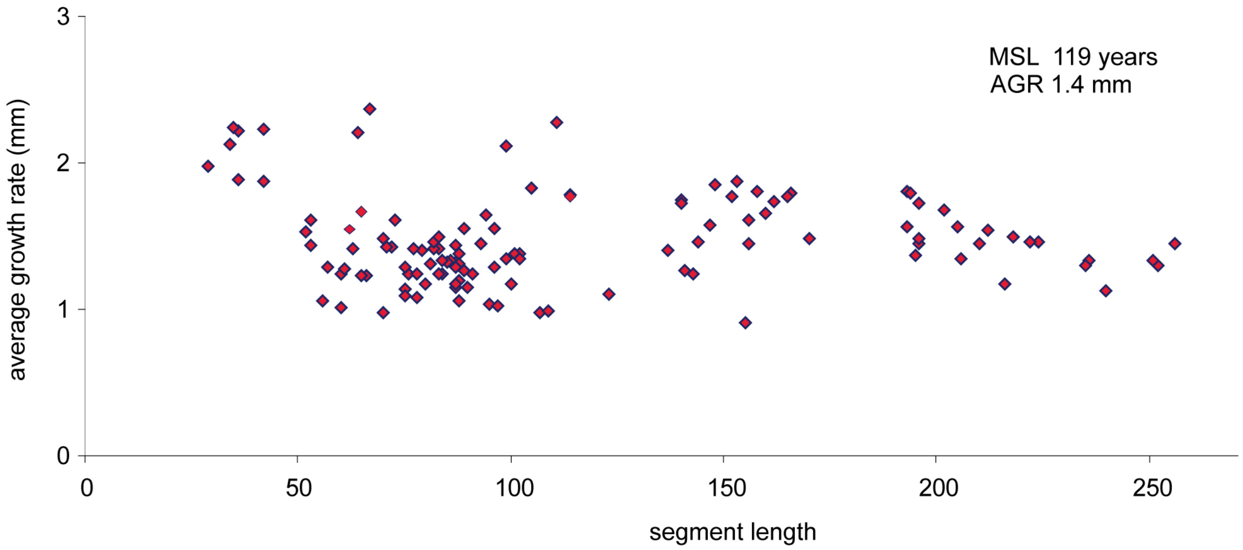

Figure S11. Relationship between average growth rate (AGR) and mean segment length (MSL) of the Altscherbitz dataset.
