# Supplementary material for: Early Neolithic Water Wells Reveal the World's Oldest Wood Architecture
Source: PLoS One. 2012 Dec 19;7(12):e51374. doi: 10.1371/journal.pone.0051374 (PMC3526582; doi:10.1371/journal.pone.0051374)
Supplement: Figure S12 — Smoothed regional curves representing the average age trend of recent oaks from Central Eastern Germany (green) and Early Neolithic oaks from the Altscherbitz well construction (red). (PDF) [file pone.0051374.s013.pdf]

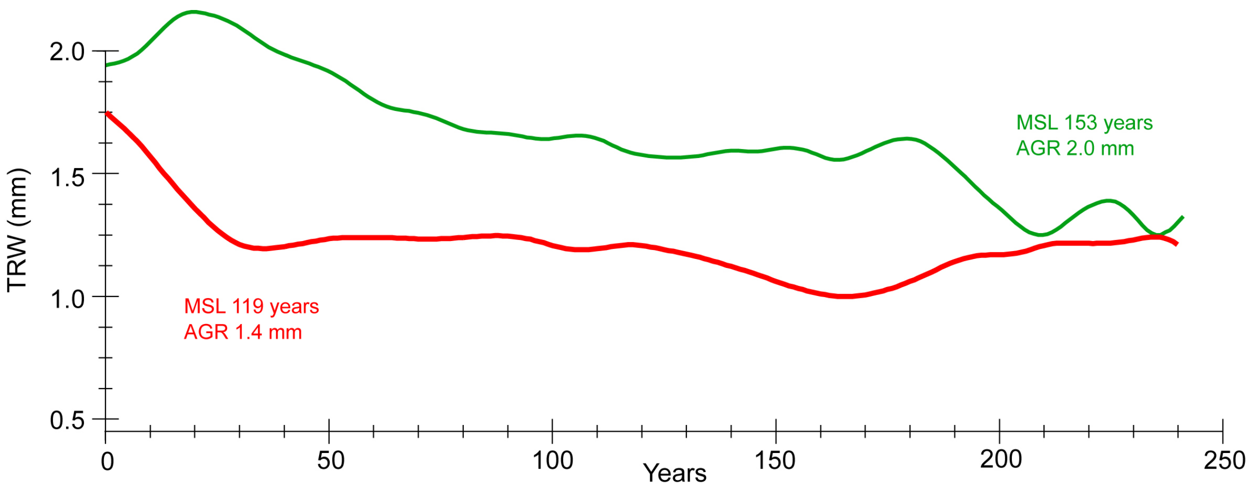

Figure S12. Smoothed regional curves representing the average age trend of recent oaks from Central Eastern Germany (green) and Early Neolithic oaks from the Altscherbitz well construction (red).
