# Supplementary material for: Early Neolithic Water Wells Reveal the World's Oldest Wood Architecture
Source: PLoS One. 2012 Dec 19;7(12):e51374. doi: 10.1371/journal.pone.0051374 (PMC3526582; doi:10.1371/journal.pone.0051374)
Supplement: Figure S13 — Synchronization of the 124 Altscherbitz tree-ring series. (A) EPS over 50 years, lagged by 25 years, (B) replication, (C) individual tree-ring series (black) in overlap with mean (red), (D) mean chronology in overlap with the chronology from the Main river valley after 10-year smoothing. (PDF) [file pone.0051374.s014.pdf]

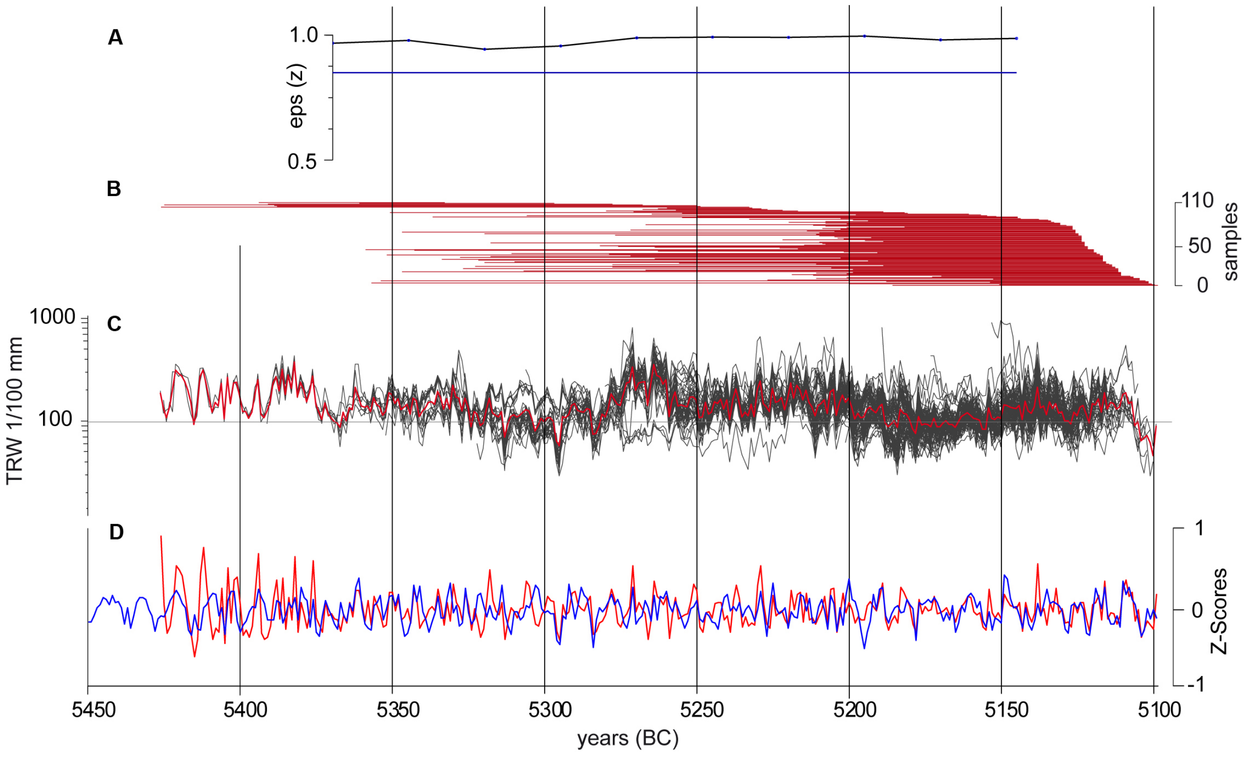

Figure S13. Synchronization of the 124 Altscherbitz tree-ring series. (**A**) EPS over 50 years, lagged by 25 years, (**B**) replication, (**C**) individual tree-ring series (black) in overlap with mean (red), (**D**) mean chronology in overlap with the chronology from the Main river valley after 10-year smoothing [1].

1. Spurk M, Friedrich M, Hofmann J, Remmele S, Frenzel B, et al. (1998) Revisions and Extensions of the Hohenheim Oak and Pine Chronologies: New Evidence about the Timing of the Younger Dryas/Preboreal Transition. Radiocarbon 40: 1107–1116.
