# Supplementary material for: Early Neolithic Water Wells Reveal the World's Oldest Wood Architecture
Source: PLoS One. 2012 Dec 19;7(12):e51374. doi: 10.1371/journal.pone.0051374 (PMC3526582; doi:10.1371/journal.pone.0051374)
Supplement: Figure S18 — (A) 18 tree-ring series from Eythra well E1 in overlap. (B) Mean chronology from E1 (red) in overlap with the Altscherbitz reference chronology (blue). (PDF) [file pone.0051374.s019.pdf]

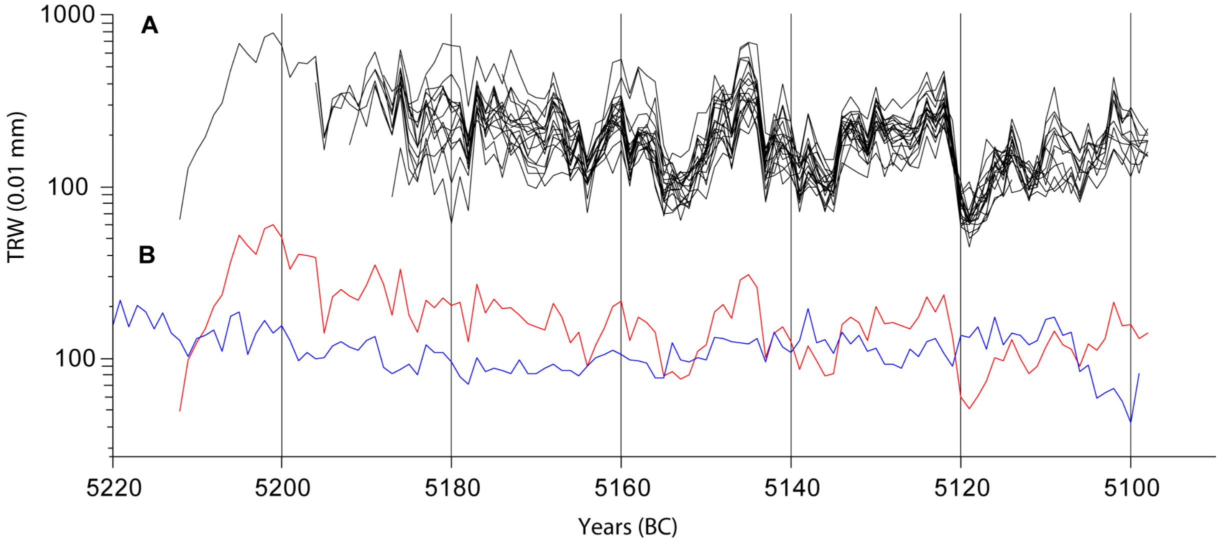

Figure S18. (**A**) 18 tree-ring series from Eythra well E1 in overlap. (**B**) Mean chronology from E1 (red) in overlap with the Altscherbitz reference chronology (blue).
