# Supplementary material for: Early Neolithic Water Wells Reveal the World's Oldest Wood Architecture
Source: PLoS One. 2012 Dec 19;7(12):e51374. doi: 10.1371/journal.pone.0051374 (PMC3526582; doi:10.1371/journal.pone.0051374)
Supplement: Figure S20 — (A) Tree-ring series from the Eythra well E2 in overlap (grey). (B) The mean chronology (red) of E2 dated against the Altscherbitz mean (blue) chronology. (PDF) [file pone.0051374.s021.pdf]

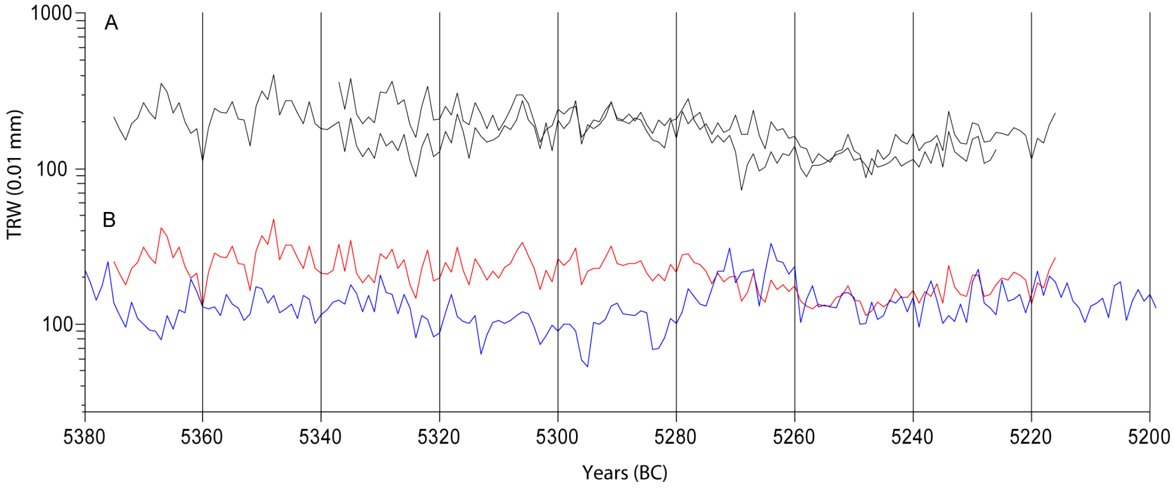

Figure S20. (**A**) Tree-ring series from the Eythra well E2 in overlap (grey). (**B**) The mean chronology (red) of E2 dated against the Altscherbitz mean (blue) chronology.
