# Supplementary material for: Early Neolithic Water Wells Reveal the World's Oldest Wood Architecture
Source: PLoS One. 2012 Dec 19;7(12):e51374. doi: 10.1371/journal.pone.0051374 (PMC3526582; doi:10.1371/journal.pone.0051374)
Supplement: Figure S21 — Environmental change in the Early Neolithic. (A) Pollen-based European temperature reconstruction, (B) subfossil-based Alpine treeline reconstruction, (C) temporal distribution of glacial 95 wood remains, and (D) peat bog-based hydroclimatic reconstruction from the UK. (PDF) [file pone.0051374.s022.pdf]

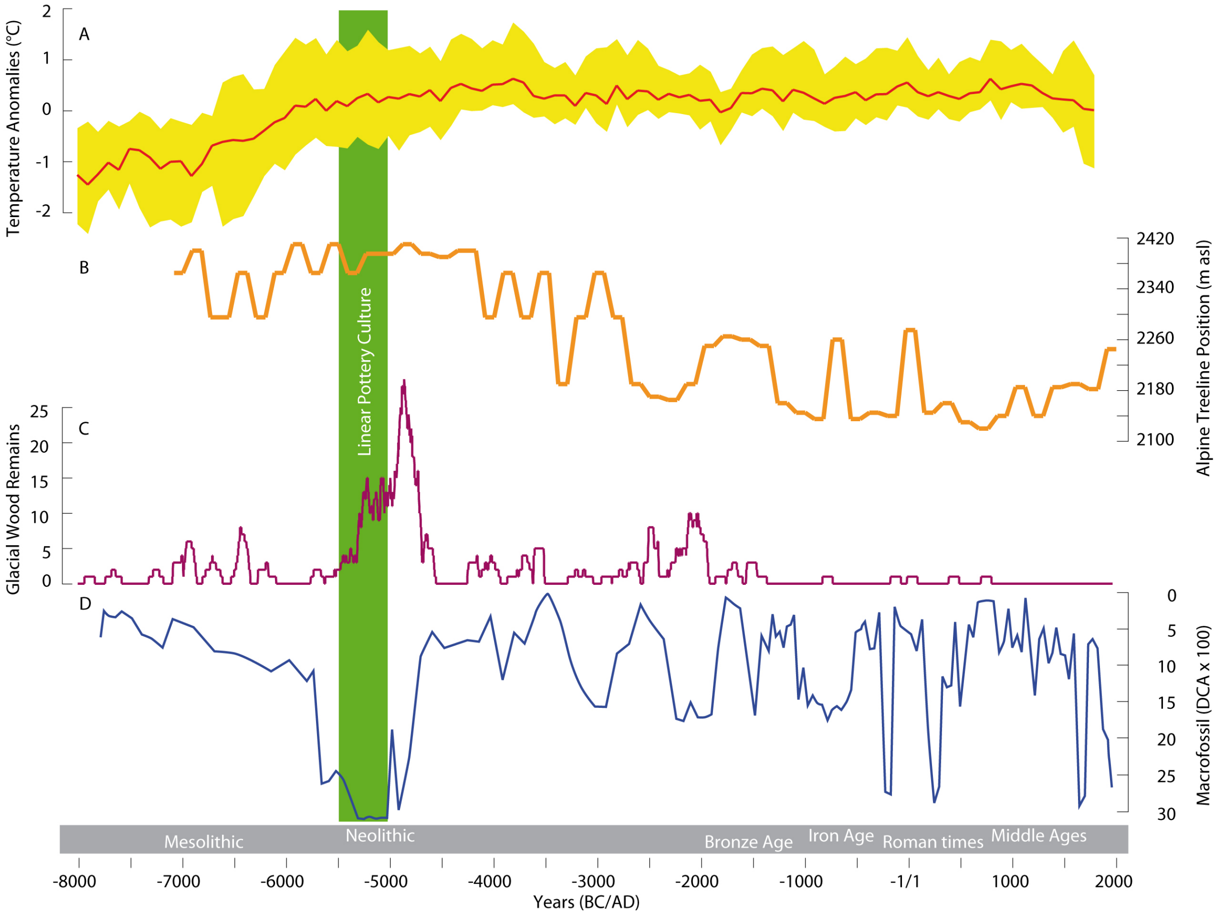

Figure S21. Environmental change in the Early Neolithic. (**A**) Pollen-based European temperature reconstruction [1], (**B**) subfossil-based Alpine treeline reconstruction [2], (**C**) temporal distribution of glacial 95 wood remains [2], and (**D**) peat bog-based hydroclimatic reconstruction from the UK [3].

1. Davis BAS et al. (2003) The temperature of Europe during the Holocene reconstructed from pollen data. Quat Sci. Rev 22: 1701–1716.
2. Nicolussi K (2009) Alpine Dendrochronologie – Untersuchungen zur Kenntnis der holozänen Umwelt- und Klimaentwicklung. In: Schmidt R, Matulla C, Psenner R, editors. Klimawandel in Österreich: die letzten 20.000 Jahre, vol. 6 of Alpine Space – Man & Environment. Innsbruck: Innsbruck University Press. pp. 41–54.
3. Barber K, Zolitschka B, Tarasov P, Lotter AF (2004) Atlantic to Urals – The Holocene Climatic Record of Mid-Latitude Europe. In: Battarbee R, Gasse F, Stickley CE, editors. Past Climate Variability through Europe and Africa. Dordrecht: Springer. pp. 417–442.
