# Supplementary material for: Early Neolithic Water Wells Reveal the World's Oldest Wood Architecture
Source: PLoS One. 2012 Dec 19;7(12):e51374. doi: 10.1371/journal.pone.0051374 (PMC3526582; doi:10.1371/journal.pone.0051374)
Supplement: Table S1 — Grid report of the correlation results between chronologies from well A, B, E1, E2 and the Main river valley. TBP = t-value after Baillie and Pilcher, THO = t-value after Hollstein, Gl = % of Gleichläufigkeit, r = correlation coefficient. (PDF) [file pone.0051374.s023.pdf]

	E2	E1	A	B
**A**	TBP: 5.61<br>THO: 5.16<br>Gl: 63.80<br>r: 0.38	TBP: 4.25<br>THO: 3.58<br>Gl: 63.60<br>r: 0.34		
**B**	TBP: 4.07<br>THO: 2.84<br>Gl: 56.60<br>r: 0.30	no overlap	TBP: 6.16<br>THO: 7.05<br>Gl: 60.80<br>r: 0.37	
**Main**	TBP: 4.19<br>THO: 3.45<br>Gl: 58.80<br>r: 0.32	TBP: 3.01<br>THO: 2.93<br>Gl: 59.60<br>r: 0.24	TBP: 7.62<br>THO: 7.80<br>Gl: 61.90<br>r: 0.38	TBP: 3.36<br>THO: 4.14<br>Gl: 57.50<br>r: 0.20

Table S1. Grid report of the correlation results between chronologies from well A, B, E1, E2 and the Main river valley. *TBP* = t-value after Baillie and Pilcher [1], *THO* = t-value after Hollstein [2], *Gl* = % of Gleichläufigkeit, *r* = correlation coefficient.

1. Baillie MGL, Pilcher JR (1993) A simple crossdating program for tree-ring research. Tree-Ring Bull 33: 7–4.
2. Hollstein E (1980) Mitteleuropäische Eichenchronologie. Mainz: Zabern.
