# Supplementary material for: Early Neolithic Water Wells Reveal the World's Oldest Wood Architecture
Source: PLoS One. 2012 Dec 19;7(12):e51374. doi: 10.1371/journal.pone.0051374 (PMC3526582; doi:10.1371/journal.pone.0051374)
Supplement: Table S2 — Tree-ring inventory. (PDF) [file pone.0051374.s024.pdf]

Table S2. Tree-ring inventory.

Well A

Sample no.	Taxa	Pith	Sap-wood	Waney edge	Tree-rings	Start year	End year	Dating	Origin
96-1285	*Quercus spp.*	-	-	-	62	-5394	-5333	after -5323	construction pit
156-3567	*Quercus spp.*	-	-	-	44	-5376	-5333	after -5323	filling
2-808	*Quercus spp.*	-	-	-	65	-5361	-5297	after -5287	construction pit
69-634	*Quercus spp.*	-	-	-	114	-5391	-5278	after -5268	construction pit
40-167	*Quercus spp.*	-	-	-	158	-5425	-5268	after -5258	construction pit
57-493	*Quercus spp.*	-	-	-	140	-5389	-5250	after -5240	construction pit
57-580	*Quercus spp.*	-	-	-	140	-5388	-5249	after -5239	construction pit
119-2141	*Quercus spp.*	-	1	-	194	-5426	-5233	-5214 ± 10	construction pit
212-3925	*Quercus spp.*	-	-	-	29	-5260	-5232	after -5222	construction pit
201-3823	*Quercus spp.*	-	-	-	35	-5263	-5229	after -5219	construction pit
94-2035	*Quercus spp.*	X	-	-	34	-5262	-5229	after -5219	construction pit
77-1518	*Quercus spp.*	X	-	-	36	-5263	-5228	after -5218	construction pit
77-1963	*Quercus spp.*	-	-	-	42	-5268	-5227	after -5217	construction pit
695	*Quercus spp.*	-	-	-	36	-5257	-5222	after -5212	construction pit
186-3717	*Quercus spp.*	X	4	-	64	-5280	-5217	5201 ± 10	construction pit
184-3426	*Quercus spp.*	-	-	-	83	-5271	-5189	after -5179	construction pit
118-1800	*Quercus spp.*	-	-	-	53	-5240	-5188	after -5178	construction pit
92-1622	*Quercus spp.*	-	-	-	170	-5351	-5182	after -5172	construction pit
171-3656	*Quercus spp.*	-	-	-	75	-5255	-5181	after -5171	construction pit
895	*Quercus spp.*	-	-	-	57	-5221	-5165	after -5155	construction pit
23-4865	*Quercus spp.*	-	-	-	105	-5265	-5161	after -5151	log construction
5-1478	*Quercus spp.*	-	-	-	42	-5198	-5157	after -5147	filling
68-804	*Quercus spp.*	-	-	-	152	-5306	-5155	after -5145	log construction
234-4635	*Quercus spp.*	-	-	-	99	-5250	-5152	after -5142	filling
50-347	*Quercus spp.*	-	-	-	111	-5255	-5145	after -5135	log construction
217-4753	*Quercus spp.*	-	-	-	193	-5337	-5145	after -5135	log construction
156-3180	*Quercus spp.*	-	-	-	60	-5203	-5144	after -5134	filling
154-3638	*Quercus spp.*	-	-	-	96	-5233	-5138	after -5128	construction pit
21-55	*Quercus spp.*	-	-	-	65	-5199	-5135	after -5125	construction pit
34-124	*Quercus spp.*	-	-	-	61	-5194	-5134	after -5124	construction pit
49-636	*Quercus spp.*	-	-	-	75	-5208	-5134	after -5124	log construction
14-20	*Quercus spp.*	-	-	-	88	-5220	-5133	after -5123	log construction
48-213	*Quercus spp.*	-	-	-	71	-5201	-5131	after -5121	construction pit
150-2669	*Quercus spp.*	-	-	-	78	-5208	-5131	after -5121	construction pit
16-4854	*Quercus spp.*	-	-	-	137	-5267	-5131	after -5121	log construction
125-2189	*Quercus spp.*	-	-	-	84	-5212	-5129	after -5119	construction pit
37-126	*Quercus spp.*	-	-	-	56	-5182	-5127	after -5117	construction pit
65-490	*Quercus spp.*	-	-	-	82	-5208	-5127	after -5117	construction pit
103-1613	*Quercus spp.*	-	-	-	77	-5203	-5127	after -5117	construction pit
97-1615	*Quercus spp.*	-	-	-	83	-5209	-5127	after -5117	construction pit
64-560	*Quercus spp.*	-	-	-	75	-5201	-5127	after -5117	construction pit
2/3-2354	*Quercus spp.*	-	-	-	86	-5212	-5127	after -5117	construction pit
88-795	*Quercus spp.*	-	-	-	80	-5205	-5126	after -5116	construction pit
170-3655	*Quercus spp.*	-	-	-	89	-5214	-5126	after -5116	construction pit
31-155	*Quercus spp.*	-	-	-	222	-5347	-5126	after -5116	log construction
27-83	*Quercus spp.*	-	-	-	147	-5272	-5126	after -5116	log construction

120-2151	*Quercus spp.*	-	-	-	85	-5210	-5126	after -5116	log construction
165-3332	*Quercus spp.*	-	-	-	87	-5211	-5125	after -5115	construction pit
25-78	*Quercus spp.*	-	-	-	196	-5320	-5125	after -5115	log construction
29-108	*Quercus spp.*	-	-	-	153	-5277	-5125	after -5115	log construction
47-176	*Quercus spp.*	-	-	-	66	-5189	-5124	after -5114	construction pit
203-3313	*Quercus spp.*	-	-	-	81	-5204	-5124	after -5114	construction pit
121-3499	*Quercus spp.*	-	-	-	82	-5205	-5124	after -5114	construction pit
181-3654	*Quercus spp.*	-	-	-	79	-5202	-5124	after -5114	construction pit
213-4225	*Quercus spp.*	-	-	-	87	-5210	-5124	after -5114	construction pit
115-1633	*Quercus spp.*	-	-	-	72	-5195	-5124	after -5114	construction pit
12-19	*Quercus spp.*	-	4	-	99	-5222	-5124	-5108 ± 10	log construction
44-187	*Quercus spp.*	-	-	-	70	-5193	-5124	after -5114	log construction
87-1611	*Quercus spp.*	X	-	-	84	-5207	-5124	after -5114	log construction
137-2059	*Quercus spp.*	-	-	-	93	-5215	-5123	after -5113	construction pit
125-2210	*Quercus spp.*	-	-	-	87	-5209	-5123	after -5113	construction pit
208-4031	*Quercus spp.*	-	-	-	88	-5210	-5123	after -5113	construction pit
24-77	*Quercus spp.*	-	-	-	160	-5282	-5123	after -5113	log construction
82-1356	*Quercus spp.*	-	-	-	86	-5208	-5123	after -5113	log construction
218-4754	*Quercus spp.*	-	-	-	196	-5318	-5123	after -5113	log construction
18-84	*Quercus spp.*	X	4	-	155	-5276	-5122	-5106 ± 10	construction pit
132-1875	*Quercus spp.*	-	2	-	96	-5217	-5122	-5104 ± 10	construction pit
17-4855	*Quercus spp.*	-	-	-	102	-5223	-5122	after -5112	log construction
30-109	*Quercus spp.*	-	-	-	143	-5264	-5122	after -5112	log construction
139-2885	*Quercus spp.*	-	8	-	83	-5202	-5120	-5108 ± 10	construction pit
32-123	*Quercus spp.*	-	-	-	224	-5343	-5120	after -5110	log construction
46-192	*Quercus spp.*	-	-	-	144	-5263	-5120	after -5110	log construction
144-3324	*Quercus spp.*	-	-	-	240	-5359	-5120	after -5110	log construction
166-3306	*Quercus spp.*	-	-	-	73	-5191	-5119	after -5109	construction pit
143-3323	*Quercus spp.*	-	2	-	193	-5311	-5119	-5101 ± 10	log construction
202-3976	*Quercus spp.*	-	-	-	162	-5279	-5118	after -5108	construction pit
148-3661	*Quercus spp.*	X	-	-	235	-5352	-5118	after -5108	log construction
117-2153	*Quercus spp.*	-	5	-	90	-5207	-5118	-5103 ± 10	log construction
95-1632	*Quercus spp.*	-	-	-	78	-5194	-5117	after -5107	construction pit
56-635	*Quercus spp.*	-	-	-	156	-5272	-5117	after -5107	log construction
67-803	*Quercus spp.*	-	-	-	202	-5318	-5117	after -5107	log construction
81-1355	*Quercus spp.*	-	-	-	206	-5322	-5117	after -5107	log construction
116-2152	*Quercus spp.*	-	-	-	148	-5264	-5117	after -5107	log construction
147-3662	*Quercus spp.*	-	-	-	218	-5334	-5117	after -5107	log construction
158-3181	*Quercus spp.*	-	13	X	87	-5203	-5117	-5117	log construction
209-4751	*Quercus spp.*	-	-	-	212	-5328	-5117	after -5107	log construction
26-82	*Quercus spp.*	-	-	-	141	-5256	-5116	after -5106	log construction
43-341	*Quercus spp.*	-	-	-	205	-5320	-5116	after -5106	log construction
123-2666	*Quercus spp.*	-	2	-	195	-5310	-5116	-5098 ± 10	log construction
86-1610	*Quercus spp.*	-	-	-	251	-5366	-5116	after -5106	log construction
151-2680	*Quercus spp.*	-	14	-	97	-5211	-5115	-5109 ± 10	construction pit
168-3495	*Quercus spp.*	-	17	X	76	-5189	-5114	-5114	construction pit
0-4135	*Quercus spp.*	-	-	-	165	-5278	-5114	after -5104	construction pit
124-2664	*Quercus spp.*	-	1	-	210	-5323	-5114	-5095 ± 10	log construction
11-2848	*Quercus spp.*	-	12	-	94	-5206	-5113	-5105 ± 10	construction pit
197-4276	*Quercus spp.*	-	-	-	166	-5278	-5113	after -5103	log construction
135-1961	*Quercus spp.*	-	15	X	88	-5199	-5112	-5111	construction pit
2/3-1764	*Quercus spp.*	-	10	-	83	-5194	-5112	-5102 ± 10	construction pit
100-1858	*Quercus spp.*	-	9	-	216	-5327	-5112	-5101 ± 10	log construction

99-1859	*Quercus spp.*	-	4	-	156	-5267	-5112	-5096 ± 10	log construction
210-4752	*Quercus spp.*	-	-	-	236	-5347	-5112	after -5102	log construction
188-4278	*Quercus spp.*	-	-	-	196	-5307	-5112	after -5102	log construction
138-2028	*Quercus spp.*	-	19	X	89	-5199	-5111	-5111	construction pit
140-2069	*Quercus spp.*	-	12	X	102	-5212	-5111	-5111	construction pit
167-3494	*Quercus spp.*	-	13	X	101	-5211	-5111	-5111	construction pit
160-2674	*Quercus spp.*	-	-	-	109	-5219	-5111	after -5101	construction pit
109-1765	*Quercus spp.*	-	12	X	91	-5201	-5111	-5111	construction pit
2/3-3109	*Quercus spp.*	-	12	X	89	-5199	-5111	-5111	construction pit
149-2665	*Quercus spp.*	-	9	-	63	-5170	-5108	-5097 ± 10	construction pit
161-2676	*Quercus spp.*	-	8	-	95	-5201	-5107	-5095 ± 10	construction pit
235-4800	*Quercus spp.*	-		-	67	-5173	-5107	after -5097	log construction
136-1962	*Quercus spp.*	-	13	-	107	-5211	-5105	-5098 ± 10	construction pit
216-4030	*Quercus spp.*	-	16	-	123	-5227	-5105	-5101 ± 10	construction pit
141-2122	*Quercus spp.*	X	1	-	49	-5153	-5105	-5087 ± 10	filling
178-4277	*Quercus spp.*	-	11	-	252	-5354	-5103	-5094 ± 10	log construction
126-1857	*Quercus spp.*	-	15	X	87	-5188	-5102	-5102	construction pit
239-4656	*Quercus spp.*	-	18	X	53	-5154	-5102	-5102	construction pit
2-2675	*Quercus spp.*	-	21	X	67	-5168	-5102	-5102	construction pit
177-4148	*Quercus spp.*	-	19	X	256	-5357	-5102	-5102	log construction
98-1480	*Quercus spp.*	-	15	-	60	-5161	-5102	-5097 ± 10	log construction
106-1618	*Quercus spp.*	-	12	-	100	-5200	-5101	-5093 ± 10	construction pit
108-1856	*Quercus spp.*	-	15	X	114	-5214	-5101	-5101	construction pit
107-1617	*Quercus spp.*	-	13	X	52	-5151	-5100	-5100	construction pit
907	*Quercus spp.*	-	13	X	88	-5186	-5099	-5099	construction pit
28-185	*Quercus spp.*	X	-	-	17	-	-	-	construction pit
133-1876	*Quercus spp.*	-	-	-	20	-	-	-	construction pit
173-3331	*Quercus spp.*	-	-	-	22	-	-	-	construction pit
54-184	*Quercus spp.*	-	-	-	29	-	-	-	construction pit
207-3737	*Quercus spp.*	-	15	-	49	-	-	-	filling
5-1131	*Quercus spp.*	-	-	-	30	-	-	-	filling
5-813	*Quercus spp.*	X	-	-	30	-	-	-	filling
5-994	*Quercus spp.*	-	-	-	47	-	-	-	filling
236-4804	*Quercus spp.*	-	8	X	29	-	-	-	log construction
83-805	*Quercus spp.*	-	-	-	26	-	-	-	log construction

Well B

Sample no.	Taxa	Pith	Sap-wood	Waney edge	Tree-rings	Start year	End year	Dating	Origin
5SW2	*Quercus spp.*	-	-	-	95	-5341	-5247	after -5237	construction pit
6SO2	*Quercus spp.*	-	-	-	127	-5365	-5239	after -5229	construction pit
4NW1	*Quercus spp.*	-	-	-	111	-5346	-5236	after -5226	construction pit
12NO3	*Quercus spp.*	-	-	-	234	-5468	-5235	after -5225	construction pit
11NW2	*Quercus spp.*	-	-	-	229	-5447	-5219	after -5209	construction pit
3SW1	*Quercus spp.*	-	1	-	142	-5351	-5210	5190 ± 10	construction pit

Well E1

Sample no.	Taxa	Pith	Sap-wood	Waney edge	Tree-rings	Start year	End year	Dating	Origin
17-11	*Quercus spp.*	-	-	-	50	-5167	-5118	after -5108	well lining
17-12	*Quercus spp.*	X	-	-	75	-5188	-5114	after -5104	well lining
17-5	*Quercus spp.*	X	-	-	74	-5187	-5114	after -5104	well lining
17-14	*Quercus spp.*	X	-	-	79	-5192	-5114	after -5104	well lining
17-13	*Quercus spp.*	-	-	-	75	-5188	-5114	after -5104	well lining
17-1	*Quercus spp.*	-	1	-	63	-5172	-5110	-5091 ± 10	well lining
17-6	*Quercus spp.*	-	-	-	71	-5179	-5109	after -5099	well lining
17-9	*Quercus spp.*	-	8	-	72	-5173	-5102	-5090 ± 10	well lining
17-7	*Quercus spp.*	-	-	-	90	-5189	-5100	after -5090	well lining
17-8	*Quercus spp.*	-	12	-	88	-5186	-5099	-5082 ± 10	well lining
17-20_5	*Quercus spp.*	-	12	X	99	-5196	-5098	-5098	well lining
17-18_4	*Quercus spp.*	-	13	X	115	-5212	-5098	-5098	well lining
17-15	*Quercus spp.*	-	12	X	89	-5186	-5098	-5098	well lining
17-19_4	*Quercus spp.*	-	11	X	99	-5196	-5098	-5098	well lining
17-10	*Quercus spp.*	-	11	X	88	-5185	-5098	-5098	well lining
17-21_5	*Quercus spp.*	-	11	X	92	-5189	-5098	-5098	well lining
17-17	*Quercus spp.*	-	13	X	77	-5174	-5098	-5098	well lining
17-4	*Quercus spp.*	-	14	X	86	-5183	-5098	-5098	well lining
17-3	*Quercus spp.*	-	15	-	89	-5186	-5098	-5098	well lining
F475	*Quercus spp.*	-	-	-	105	-	-	-	filling
17-16	*Quercus spp.*	-	-	-	75	-	-	-	well lining

Well E2

Sample no.	Taxa	Pith	Sap-wood	Waney edge	Tree-rings	Start year	End year	Dating	Origin
21-3	*Quercus spp.*	-	-	-	150	-5375	-5226	after -5216	well lining
21-2	*Quercus spp.*	-	-	-	122	-5337	-5216	after -5206	well lining
