# Supplementary material for: Early Neolithic Water Wells Reveal the World's Oldest Wood Architecture
Source: PLoS One. 2012 Dec 19;7(12):e51374. doi: 10.1371/journal.pone.0051374 (PMC3526582; doi:10.1371/journal.pone.0051374)
Supplement: Data S1 — Tree-ring width data (Tucson Format). (PDF) [file pone.0051374.s025.pdf]

Data S1

Tree-Ring Width Data

Tree-ring width values 1/100 mm (Tucson format)
A: Altscherbitz well
B: Brodau well
E1: Eythra well 1
E2: Eythra well 2

```
A00001 -5394   258   120   113   144
A00001 -5390   156   247   275   226   363   175   319   196   417   181
A00001 -5380   232   226   163   218   240   211   171   125   208   156
A00001 -5370   107    93   102   153   148   123   175   171   356   357
A00001 -5360   192   173   155    96   129   105    96    82   121   161
A00001 -5350   158   214   176   133   165   208   102   207   210   142
A00001 -5340   198   169   205   207   180   247   181   118   999
A00002 -5376   326   145   125   115   189   207
A00002 -5370   182   123   144   114   140   113   128   112   177   126
A00002 -5360   124   153   151   160   196   160   146   107   169   229
A00002 -5350   225   201   215   159   212   208   142   231   208   164
A00002 -5340   194   243   202   179   232   283   250   248   999
A00003 -5361   125
A00003 -5360   132   123   118   122   188   141   148   102   129   237
A00003 -5350   173   165   188   130   178   173   135   205   170   149
A00003 -5340   161   199   188   226   188   263   218   203   226   181
A00003 -5330   212   132   125   124   148   159   113   136   140   103
A00003 -5320   120   161   227   203   167   188   184   108   163   178
A00003 -5310   156   178   242   212   216   198   185   120   151   156
A00003 -5300   156   190   170   150   999
A00004 -5391    94
A00004 -5390   169   264   360   258   432   231   336   247   434   172
A00004 -5380   259   234   152   190   284   144   102   100   136   119
A00004 -5370   103    77   115   113   221   135   146   142   164   178
A00004 -5360   130   130   156   138   153   149   117   100   115   199
A00004 -5350   261   170   169   136   156   174   107   167   202   131
A00004 -5340   156   170   188   204   220   278   244   176   230   201
A00004 -5330   159   132   102   136   162   144   103   131   167   128
A00004 -5320   108   129   216   231   167   167   219   136   139   136
A00004 -5310   155   173   226   174   215   190   208   128   136   144
A00004 -5300   163   193   151   142   114   110   192   182   158   171
A00004 -5290   164   102   115    71   153   145   104   107   123   126
A00004 -5280   124   115   107   999
A00005 -5425   158    98   153   123   357
A00005 -5420   334   283   272   264   204    96   111   241   329   219
A00005 -5410   137   137   181   156   258    99   268   158   251   221
A00005 -5400   175   141   123   110   125   213   247    97   112   147
A00005 -5390   178   260   331   222   353   202   308   250   402   169
A00005 -5380   213   207   148   195   310   138   135   103   150   111
A00005 -5370   110   110    96    70    95    76   131   105   144   131
A00005 -5360   112   119   135   139   180   146   108   117   138   219
A00005 -5350   282   199   203   144   181   195   123   225   210   135
A00005 -5340   198   150   185   204   206   272   229   175   195   180
A00005 -5330   167   143   126   117   154   116    98   122   149   114
A00005 -5320   119   161   198   220   157   154   227   138   155   135
A00005 -5310   165   182   211   225   229   221   222   122   138   149
A00005 -5300   176   215   170   134   139   124   227   172   136   145
A00005 -5290   152   108    98    75   166   126   106    97   117   112
A00005 -5280    98   142   118    80   143   190    75    63    71    71
A00005 -5270    81    65    93   999
A00006 -5389   228   268   204   293   186   260   209   404   195
A00006 -5380   268   212   185   209   267   137   116    99   143   117
A00006 -5370   127   115   102    79   107   100   125   131   198   149
A00006 -5360   131   125   159   151   227   139   153   100   134   199
A00006 -5350   220   150   169   131   191   169   139   188   169   120
A00006 -5340   172   158   195   214   185   256   214   183   239   182
A00006 -5330   190   157   105   122   166   159   113   129   126   119
A00006 -5320   124   162   238   201   157   179   223   118   169   172
A00006 -5310   151   178   215   193   218   189   207   115   132   161
A00006 -5300   166   171   163   137   102   118   201   166   146   186
```

A00006	-5290	156	100	84	82	120	134	105	85	90	102
A00006	-5280	100	108	110	90	93	80	57	62	60	68
A00006	-5270	72	70	63	84	58	63	90	86	81	73
A00006	-5260	69	51	69	88	100	69	66	67	70	89
A00006	-5250	66	999								
A00007	-5388	202	144	275	160	271	211	377	183		
A00007	-5380	283	217	157	199	293	136	124	95	158	126
A00007	-5370	112	119	97	71	93	111	137	127	163	156
A00007	-5360	172	156	147	147	197	156	157	111	179	177
A00007	-5350	212	169	175	126	215	196	144	231	169	137
A00007	-5340	160	183	191	212	199	276	240	180	255	211
A00007	-5330	185	136	119	136	172	165	99	129	142	125
A00007	-5320	126	180	231	208	165	186	224	141	163	173
A00007	-5310	182	200	217	225	232	181	224	130	133	163
A00007	-5300	177	185	172	143	131	129	214	170	146	218
A00007	-5290	172	105	93	94	144	129	117	97	97	114
A00007	-5280	114	118	112	95	100	100	50	71	65	61
A00007	-5270	72	79	72	75	72	72	94	83	80	82
A00007	-5260	69	60	68	91	98	85	69	69	88	96
A00007	-5250	105	118	999							
A00008	-5426	195	120	127	134	302	297				
A00008	-5420	270	289	234	154	118	97	154	325	330	276
A00008	-5410	202	118	137	135	229	122	290	152	239	279
A00008	-5400	194	118	137	113	132	160	285	149	115	140
A00008	-5390	143	217	265	183	281	186	270	185	336	181
A00008	-5380	243	170	168	190	318	155	125	125	149	118
A00008	-5370	121	107	97	56	103	91	125	136	310	182
A00008	-5360	152	193	142	156	205	158	168	111	166	235
A00008	-5350	150	175	163	120	148	163	139	184	159	122
A00008	-5340	179	172	171	208	193	266	219	182	237	172
A00008	-5330	182	148	117	133	149	146	122	134	124	117
A00008	-5320	131	153	227	196	172	182	233	124	160	194
A00008	-5310	142	162	250	236	202	177	214	119	141	167
A00008	-5300	139	207	170	130	118	122	260	196	146	223
A00008	-5290	183	110	82	65	124	137	122	93	117	141
A00008	-5280	112	120	140	93	114	99	81	79	64	66
A00008	-5270	76	83	74	86	71	72	102	86	85	90
A00008	-5260	83	61	72	101	97	84	65	85	77	108
A00008	-5250	110	85	73	75	90	83	81	74	84	107
A00008	-5240	96	86	104	96	83	71	102	80	999	
A00009	-5260	339	150	196	283	220	233	264	259	268	255
A00009	-5250	310	199	171	201	178	145	172	237	205	148
A00009	-5240	105	122	205	155	145	95	157	170	137	999
A00010	-5262	276	397								
A00010	-5260	500	140	205	292	230	245	271	306	262	362
A00010	-5250	417	275	252	297	258	233	165	243	182	148
A00010	-5240	141	117	154	116	78	75	102	104	91	83
A00010	-5230	104	107	999							
A00011	-5263	207	297	358							
A00011	-5260	466	210	366	313	320	280	322	279	240	265
A00011	-5250	221	177	154	257	335	213	227	263	184	132
A00011	-5240	114	118	228	249	194	109	97	110	106	78
A00011	-5230	134	231	999							
A00012	-5263	193	259	310							
A00012	-5260	422	149	241	277	292	209	281	276	261	316
A00012	-5250	367	245	258	240	292	216	238	279	229	159
A00012	-5240	144	134	173	132	167	78	115	130	133	139
A00012	-5230	208	203	241	999						
A00013	-5268	233	154	93	109	244	164	253	291		
A00013	-5260	375	154	267	331	298	319	352	315	276	327
A00013	-5250	296	279	197	257	369	209	259	237	211	222
A00013	-5240	138	121	219	219	161	113	134	156	126	115
A00013	-5230	186	213	181	185	999					
A00014	-5257	292	234	227	190	248	289	355			
A00014	-5250	257	116	90	115	136	134	126	164	172	107
A00014	-5240	135	86	202	182	161	114	155	136	119	119
A00014	-5230	201	285	204	144	160	289	209	310	337	999
A00015	-5280	219	227	212	266	113	195	148	236	238	280
A00015	-5270	211	230	252	157	194	205	260	124	162	216
A00015	-5260	228	187	231	295	269	248	235	251	336	383
A00015	-5250	257	118	87	122	240	157	193	245	186	131
A00015	-5240	106	101	129	198	189	129	225	172	114	121
A00015	-5230	247	324	252	214	229	310	250	324	483	424

A00015	-5220	133	305	296	271	999					
A00016	-5271	217									
A00016	-5270	149	183	151	176	104	184	291	182	201	178
A00016	-5260	225	96	134	160	165	209	165	138	207	195
A00016	-5250	157	127	81	124	85	139	165	130	166	133
A00016	-5240	162	88	127	197	149	89	110	103	172	105
A00016	-5230	231	289	151	82	109	157	151	121	128	125
A00016	-5220	166	257	181	182	211	145	210	163	150	75
A00016	-5210	122	110	88	85	158	178	88	158	178	167
A00016	-5200	190	96	99	94	83	94	125	109	123	127
A00016	-5190	141	141	999							
A00017	-5240	193	106	192	199	172	127	132	102	139	109
A00017	-5230	193	202	132	136	120	198	147	141	169	108
A00017	-5220	176	233	141	199	248	150	165	156	169	83
A00017	-5210	89	111	138	112	191	251	123	149	151	113
A00017	-5200	171	116	88	81	105	93	106	122	115	117
A00017	-5190	128	96	83	999						
A00018	-5351	177									
A00018	-5350	161	132	205	168	153	127	165	192	228	107
A00018	-5340	78	125	110	134	137	202	144	125	120	145
A00018	-5330	287	163	115	89	181	145	101	118	87	84
A00018	-5320	99	114	147	96	118	80	87	55	78	105
A00018	-5310	93	105	107	115	78	137	107	67	62	97
A00018	-5300	82	93	102	78	76	41	102	122	110	138
A00018	-5290	142	136	124	134	170	197	92	95	96	124
A00018	-5280	113	153	167	159	157	142	187	405	364	425
A00018	-5270	248	269	308	284	191	269	454	366	315	254
A00018	-5260	198	140	137	140	112	130	84	89	102	115
A00018	-5250	129	102	82	124	96	103	128	134	128	102
A00018	-5240	113	81	115	157	131	95	83	100	140	96
A00018	-5230	125	190	130	110	109	168	125	131	143	113
A00018	-5220	191	156	142	197	191	150	197	207	135	73
A00018	-5210	117	135	157	157	188	156	145	169	144	147
A00018	-5200	172	109	110	96	121	87	108	164	131	144
A00018	-5190	180	193	120	102	114	112	88	82	109	999
A00019	-5255	167	132	173	142	187					
A00019	-5250	149	138	136	165	85	132	177	125	146	141
A00019	-5240	182	91	165	197	168	99	136	123	204	115
A00019	-5230	237	232	129	147	110	167	107	105	155	101
A00019	-5220	192	270	204	293	206	169	172	99	82	64
A00019	-5210	95	101	100	82	125	180	99	181	140	109
A00019	-5200	105	89	61	85	113	91	67	105	94	95
A00019	-5190	95	63	51	57	49	66	53	78	65	73
A00019	-5180	999									
A00020	-5221	146									
A00020	-5220	206	268	185	292	242	186	182	155	141	78
A00020	-5210	111	125	157	105	170	320	131	155	189	116
A00020	-5200	189	115	62	77	111	77	102	122	119	109
A00020	-5190	130	102	78	69	80	101	97	109	122	113
A00020	-5180	94	112	74	125	104	94	89	93	87	90
A00020	-5170	93	101	135	118	86	73	999			
A00021	-5265	383	643	426	477	316					
A00021	-5260	335	141	170	222	152	133	124	128	209	254
A00021	-5250	197	85	93	261	90	135	262	164	219	160
A00021	-5240	195	141	270	273	155	119	170	147	224	122
A00021	-5230	311	415	174	197	165	354	228	139	136	131
A00021	-5220	200	306	238	356	312	196	342	211	158	81
A00021	-5210	104	148	106	85	211	184	85	140	167	130
A00021	-5200	247	119	78	95	99	66	90	136	122	157
A00021	-5190	158	151	73	85	72	96	68	116	116	127
A00021	-5180	102	78	66	89	80	130	104	107	108	75
A00021	-5170	99	117	140	114	111	101	91	121	92	125
A00021	-5160	999									
A00022	-5198	100	145	115	69	138	142	180	83		
A00022	-5190	144	197	102	121	320	239	135	281	288	210
A00022	-5180	239	107	103	270	166	282	200	307	325	227
A00022	-5170	262	160	160	183	185	148	191	222	191	202
A00022	-5160	281	116	143	198	999					
A00023	-5306	317	270	196	191	159	149				
A00023	-5300	161	187	155	163	132	91	135	126	175	226
A00023	-5290	243	230	217	185	206	172	111	166	208	147
A00023	-5280	124	188	206	167	166	153	223	254	205	367
A00023	-5270	185	186	169	131	147	217	277	284	304	271

A00023	-5260	207	140	385	330	188	234	180	182	174	186
A00023	-5250	196	147	148	158	147	107	156	143	143	123
A00023	-5240	108	103	140	168	190	139	103	117	121	105
A00023	-5230	124	124	112	115	125	208	172	150	133	111
A00023	-5220	129	154	119	139	139	118	142	110	86	146
A00023	-5210	190	184	169	115	204	180	96	113	158	125
A00023	-5200	102	107	132	135	146	117	164	119	126	129
A00023	-5190	162	202	138	123	116	145	135	160	187	224
A00023	-5180	134	177	94	176	146	143	137	114	126	108
A00023	-5170	102	100	104	83	87	86	89	80	87	107
A00023	-5160	109	103	90	104	103	100	999			
A00024	-5250	240	175	194	275	170	196	156	164	194	161
A00024	-5240	205	162	251	273	220	216	302	214	215	225
A00024	-5230	278	281	233	262	228	264	237	215	212	253
A00024	-5220	213	264	220	245	306	155	266	198	168	217
A00024	-5210	293	242	236	136	275	314	182	220	244	230
A00024	-5200	237	185	220	185	174	149	173	257	260	167
A00024	-5190	255	211	166	131	186	246	215	222	256	201
A00024	-5180	190	169	199	281	228	203	200	138	187	166
A00024	-5170	164	159	180	169	145	177	150	148	198	168
A00024	-5160	135	173	153	182	153	131	216	162	200	999
A00025	-5337	124	105	159	135	111	125	92			
A00025	-5330	174	203	140	71	118	164	75	123	126	71
A00025	-5320	69	106	166	62	71	106	125	49	63	93
A00025	-5310	111	120	82	87	98	106	68	81	83	96
A00025	-5300	94	103	110	93	42	35	84	108	110	115
A00025	-5290	130	110	119	146	140	161	79	70	64	135
A00025	-5280	142	168	323	223	222	215	257	382	415	536
A00025	-5270	312	191	255	252	164	306	543	460	442	306
A00025	-5260	265	114	133	180	148	129	100	143	184	153
A00025	-5250	117	75	71	155	96	128	115	126	141	139
A00025	-5240	153	85	183	208	134	120	101	95	114	88
A00025	-5230	203	263	162	154	134	199	148	114	138	106
A00025	-5220	172	295	161	280	182	189	280	188	148	83
A00025	-5210	96	96	111	93	192	215	118	150	192	136
A00025	-5200	146	96	90	119	94	96	83	133	141	123
A00025	-5190	125	135	85	69	69	65	56	142	109	98
A00025	-5180	96	81	61	81	85	105	96	93	111	84
A00025	-5170	75	96	98	101	77	82	65	94	82	106
A00025	-5160	93	108	74	119	84	84	121	92	97	103
A00025	-5150	93	122	128	127	112	150	999			
A00026	-5255	277	295	286	351	460					
A00026	-5250	447	159	132	217	258	163	183	166	153	123
A00026	-5240	124	101	137	205	181	125	242	211	141	236
A00026	-5230	602	665	413	136	112	217	108	165	211	154
A00026	-5220	125	332	366	456	586	317	437	229	304	325
A00026	-5210	263	300	549	301	452	402	182	178	299	182
A00026	-5200	200	247	155	201	175	161	287	197	187	174
A00026	-5190	216	183	151	147	123	200	130	293	189	157
A00026	-5180	138	71	77	189	112	108	100	107	138	114
A00026	-5170	91	121	101	88	97	89	140	167	113	145
A00026	-5160	144	134	129	92	88	71	138	98	77	95
A00026	-5150	82	131	118	97	113	126	999			
A00027	-5203	96	113	130							
A00027	-5200	122	173	77	71	70	67	86	120	114	114
A00027	-5190	93	110	102	84	109	110	80	73	119	113
A00027	-5180	114	96	82	148	129	132	119	91	130	80
A00027	-5170	90	106	110	120	125	125	91	92	88	104
A00027	-5160	82	88	87	80	62	65	80	93	104	117
A00027	-5150	80	117	122	105	113	104	82	999		
A00028	-5233	188	179	129							
A00028	-5230	167	165	150	132	181	293	281	204	142	130
A00028	-5220	150	178	177	189	180	122	209	191	122	309
A00028	-5210	230	236	178	89	235	248	136	138	207	174
A00028	-5200	175	174	162	156	148	126	180	142	175	142
A00028	-5190	156	180	110	103	116	107	128	136	159	159
A00028	-5180	103	98	125	154	119	134	165	97	148	141
A00028	-5170	126	135	181	125	186	147	139	139	117	181
A00028	-5160	170	144	156	143	108	103	190	96	92	129
A00028	-5150	106	119	78	202	132	175	156	144	171	120
A00028	-5140	123	135	184	999						
A00029	-5199	203	140	151	132	181	192	221	184	157	
A00029	-5190	208	177	133	128	103	139	107	138	140	131

A00029	-5180	117	94	95	142	105	109	98	88	102	101
A00029	-5170	100	90	73	95	111	87	121	130	119	120
A00029	-5160	152	128	127	109	106	82	140	107	62	75
A00029	-5150	79	101	91	101	99	118	125	130	158	113
A00029	-5140	101	144	149	128	120	123	999			
A00030	-5208	156	120	182	157	88	200	199	132		
A00030	-5200	153	142	127	101	102	153	151	181	116	105
A00030	-5190	164	127	115	109	85	79	95	113	94	113
A00030	-5180	88	72	75	99	97	79	83	81	80	60
A00030	-5170	56	85	83	87	77	83	101	90	96	98
A00030	-5160	97	107	112	76	91	86	102	76	73	81
A00030	-5150	56	97	88	105	110	130	147	140	198	122
A00030	-5140	122	117	121	74	105	128	124	999		
A00031	-5194	123	168	141	150						
A00031	-5190	181	143	114	113	122	132	120	182	163	142
A00031	-5180	131	89	97	109	100	105	88	91	97	101
A00031	-5170	88	107	84	83	77	84	122	137	133	144
A00031	-5160	155	118	159	101	101	86	134	122	124	91
A00031	-5150	96	108	130	130	152	176	177	134	259	186
A00031	-5140	129	133	218	103	127	131	141	999		
A00032	-5220	145	153	108	178	196	147	214	158	147	52
A00032	-5210	78	97	112	84	181	163	86	119	167	141
A00032	-5200	155	82	76	70	70	68	73	98	114	118
A00032	-5190	96	91	62	71	71	44	70	84	83	93
A00032	-5180	80	75	54	64	77	106	92	88	90	79
A00032	-5170	91	91	103	101	88	81	65	80	84	91
A00032	-5160	96	96	72	87	74	83	116	112	95	116
A00032	-5150	95	125	139	122	115	116	132	103	166	76
A00032	-5140	92	124	165	236	169	163	88	85	999	
A00033	-5201	158									
A00033	-5200	157	230	145	171	146	173	168	204	144	171
A00033	-5190	196	175	146	147	151	146	128	207	201	181
A00033	-5180	149	123	110	148	119	121	108	95	143	121
A00033	-5170	109	122	110	98	89	106	120	151	170	142
A00033	-5160	162	139	167	120	96	90	159	133	109	115
A00033	-5150	110	135	148	135	147	167	198	136	233	164
A00033	-5140	123	138	185	112	117	122	112	111	105	124
A00033	-5130	999									
A00034	-5208	210	145	225	208	124	311	248	133		
A00034	-5200	265	200	152	119	116	147	175	201	162	118
A00034	-5190	217	190	129	86	83	93	87	94	91	75
A00034	-5180	72	73	84	111	78	63	70	71	76	78
A00034	-5170	65	75	68	59	78	57	93	73	86	96
A00034	-5160	97	89	83	54	60	61	92	63	52	62
A00034	-5150	64	88	72	77	92	97	89	88	117	82
A00034	-5140	85	107	118	68	107	105	104	80	83	105
A00034	-5130	999									
A00035	-5267	214	148	257	384	226	311	316			
A00035	-5260	342	122	182	255	174	133	177	175	281	223
A00035	-5250	240	120	157	162	98	124	151	144	155	120
A00035	-5240	165	110	147	189	146	104	104	77	120	102
A00035	-5230	179	176	124	124	104	149	97	116	171	135
A00035	-5220	203	230	143	149	201	168	148	135	91	85
A00035	-5210	78	76	99	71	134	159	66	114	114	117
A00035	-5200	121	85	84	95	91	79	101	110	117	113
A00035	-5190	89	81	80	62	70	62	53	55	57	75
A00035	-5180	60	63	70	92	70	78	78	69	85	59
A00035	-5170	56	85	74	89	77	84	69	80	83	84
A00035	-5160	83	105	81	94	73	68	149	111	103	107
A00035	-5150	85	91	150	107	106	77	99	86	107	88
A00035	-5140	115	132	197	132	140	78	136	128	115	122
A00035	-5130	999									
A00036	-5212	217	202								
A00036	-5210	272	299	211	134	208	210	128	206	205	194
A00036	-5200	173	191	107	121	119	141	146	153	149	117
A00036	-5190	179	173	102	109	100	139	124	175	157	154
A00036	-5180	127	102	86	154	107	98	103	97	99	91
A00036	-5170	99	99	96	80	101	94	140	135	138	142
A00036	-5160	160	120	133	100	84	98	139	102	87	87
A00036	-5150	79	128	115	117	122	150	167	112	215	139
A00036	-5140	109	152	108	88	107	119	134	88	85	94
A00036	-5130	89	83	999							
A00037	-5212	227	210								

A00037	-5210	324	258	194	137	208	191	138	196	218	216
A00037	-5200	190	193	127	154	116	141	176	151	162	138
A00037	-5190	187	194	133	145	130	170	141	170	147	157
A00037	-5180	140	138	77	191	131	86	115	102	107	98
A00037	-5170	96	116	83	87	89	84	133	161	133	167
A00037	-5160	162	128	118	120	107	99	135	102	90	83
A00037	-5150	76	105	109	101	107	130	156	110	175	122
A00037	-5140	95	110	132	118	111	128	151	103	124	120
A00037	-5130	95	93	100	82	999					
A00038	-5182	185	150								
A00038	-5180	134	121	110	139	124	88	96	147	159	108
A00038	-5170	64	74	87	97	84	82	113	116	134	128
A00038	-5160	110	101	130	93	82	80	94	88	93	108
A00038	-5150	78	94	123	122	99	113	109	88	111	90
A00038	-5140	63	95	120	82	114	112	117	86	84	113
A00038	-5130	116	78	89	98	999					
A00039	-5203	159	224	204							
A00039	-5200	200	216	135	167	151	169	214	173	162	142
A00039	-5190	212	171	134	118	121	149	125	196	167	141
A00039	-5180	127	94	95	138	113	83	108	94	102	103
A00039	-5170	93	97	91	86	94	99	127	143	140	129
A00039	-5160	132	118	139	89	80	122	123	97	118	82
A00039	-5150	70	111	127	131	90	119	145	113	140	155
A00039	-5140	98	113	147	114	148	147	300	338	291	239
A00039	-5130	214	147	133	128	999					
A00040	-5208	654	496	455	649	160	239	412	273		
A00040	-5200	271	308	153	146	129	150	154	167	158	166
A00040	-5190	159	151	105	109	113	126	103	173	134	96
A00040	-5180	104	59	75	117	79	68	84	80	118	134
A00040	-5170	96	84	94	62	76	54	118	120	120	128
A00040	-5160	117	99	137	83	75	85	140	94	88	77
A00040	-5150	94	133	98	116	118	143	162	115	185	134
A00040	-5140	103	106	114	91	104	121	111	88	106	94
A00040	-5130	95	90	104	99	999					
A00041	-5201	199									
A00041	-5200	190	154	141	142	120	157	167	187	135	126
A00041	-5190	147	160	117	130	148	151	132	185	116	117
A00041	-5180	95	81	76	131	88	78	68	61	99	82
A00041	-5170	62	119	70	85	83	63	114	135	134	117
A00041	-5160	160	125	127	99	85	77	133	87	79	70
A00041	-5150	81	104	110	101	99	110	120	110	185	161
A00041	-5140	104	109	139	80	93	106	112	98	104	86
A00041	-5130	100	78	77	85	999					
A00042	-5209	253	166	137	195	192	117	161	176	154	
A00042	-5200	175	184	135	141	128	149	177	160	146	133
A00042	-5190	185	145	123	111	112	128	129	176	142	129
A00042	-5180	119	100	82	149	93	92	87	78	117	98
A00042	-5170	98	111	86	87	99	97	145	147	145	135
A00042	-5160	140	121	139	120	85	88	126	99	88	102
A00042	-5150	80	102	116	133	111	133	140	129	188	105
A00042	-5140	96	105	126	86	108	114	124	94	100	88
A00042	-5130	95	66	87	82	999					
A00043	-5347	159	102	147	169	143	203	147			
A00043	-5340	89	133	160	154	136	184	147	105	117	84
A00043	-5330	190	208	92	95	164	129	94	138	142	93
A00043	-5320	101	139	205	128	129	109	122	77	73	87
A00043	-5310	116	91	76	111	118	108	70	67	80	80
A00043	-5300	77	71	106	88	54	39	91	97	123	171
A00043	-5290	173	143	141	156	132	197	80	78	77	122
A00043	-5280	122	123	205	143	169	140	185	297	188	208
A00043	-5270	188	229	193	295	164	306	433	374	280	275
A00043	-5260	310	102	145	209	121	123	99	126	159	152
A00043	-5250	150	94	113	143	125	105	192	151	175	159
A00043	-5240	198	132	162	166	170	108	122	111	156	89
A00043	-5230	211	228	159	141	180	247	142	131	137	143
A00043	-5220	157	240	242	240	219	175	315	116	155	45
A00043	-5210	90	116	142	94	178	342	112	127	201	132
A00043	-5200	270	101	63	62	66	63	80	112	116	141
A00043	-5190	166	141	86	57	92	95	90	119	107	118
A00043	-5180	121	97	63	128	101	84	96	86	124	96
A00043	-5170	94	110	102	93	98	87	102	101	109	146
A00043	-5160	80	131	117	127	125	94	175	152	143	161
A00043	-5150	110	182	164	180	213	62	72	68	92	78

A00043	-5140	108	134	204	135	160	91	149	149	154	129
A00043	-5130	139	115	98	92	110	999				
A00044	-5205	204	182	154	199	169					
A00044	-5200	203	212	136	156	118	153	168	152	144	127
A00044	-5190	161	161	141	117	137	150	109	220	174	146
A00044	-5180	136	83	85	112	104	90	80	81	89	84
A00044	-5170	69	99	85	68	88	72	108	144	140	131
A00044	-5160	118	119	115	81	71	63	122	84	77	67
A00044	-5150	60	83	88	83	88	99	112	116	180	169
A00044	-5140	99	97	152	103	100	104	121	100	79	102
A00044	-5130	96	70	75	61	99	999				
A00045	-5214	301	187	327	324						
A00045	-5210	327	266	290	183	236	299	165	190	214	199
A00045	-5200	196	254	144	182	121	183	189	220	175	123
A00045	-5190	218	194	138	128	150	162	109	183	157	122
A00045	-5180	156	108	86	134	118	108	112	115	133	100
A00045	-5170	104	137	116	98	104	100	139	192	209	171
A00045	-5160	186	122	154	107	120	82	137	71	69	84
A00045	-5150	99	101	108	111	126	174	152	146	194	155
A00045	-5140	133	146	200	127	152	147	131	136	128	134
A00045	-5130	105	89	95	73	146	999				
A00046	-5272	193	361								
A00046	-5270	202	377	339	310	158	269	332	225	342	261
A00046	-5260	277	93	176	178	119	191	188	162	177	171
A00046	-5250	157	133	154	134	99	125	169	128	157	128
A00046	-5240	153	114	147	231	159	107	117	141	115	94
A00046	-5230	193	219	123	122	119	193	171	166	192	144
A00046	-5220	212	231	139	239	152	145	167	130	104	101
A00046	-5210	111	109	112	85	175	143	93	144	119	145
A00046	-5200	155	83	80	115	102	61	110	95	104	112
A00046	-5190	111	106	80	69	71	45	70	62	81	83
A00046	-5180	76	61	66	63	102	103	66	65	100	96
A00046	-5170	97	107	125	92	83	88	79	68	94	110
A00046	-5160	90	88	73	81	91	71	138	105	96	115
A00046	-5150	96	119	149	109	104	122	122	89	135	105
A00046	-5140	124	171	211	155	188	126	159	110	122	115
A00046	-5130	107	117	87	85	96	999				
A00047	-5210	311	247	201	144	207	201	123	151	198	186
A00047	-5200	202	201	125	158	151	177	203	168	155	135
A00047	-5190	183	151	120	116	109	131	119	178	151	128
A00047	-5180	130	104	83	128	115	87	90	90	110	110
A00047	-5170	90	112	92	81	104	73	131	164	131	135
A00047	-5160	154	123	135	112	95	87	124	107	82	85
A00047	-5150	81	125	109	133	118	151	135	134	189	127
A00047	-5140	112	120	137	104	112	144	120	89	98	108
A00047	-5130	101	68	86	92	117	999				
A00048	-5277	197	169	210	250	348	327	580			
A00048	-5270	317	304	331	372	168	320	473	378	280	194
A00048	-5260	207	101	162	208	358	386	303	224	304	240
A00048	-5250	231	91	97	114	75	111	172	123	144	102
A00048	-5240	139	79	112	176	122	87	105	85	123	96
A00048	-5230	206	293	179	178	142	219	168	174	159	126
A00048	-5220	189	252	178	244	207	226	236	191	120	73
A00048	-5210	84	90	97	94	181	211	149	178	180	114
A00048	-5200	214	113	86	119	113	88	88	106	111	101
A00048	-5190	93	140	82	55	59	53	45	68	51	85
A00048	-5180	54	56	50	64	78	84	61	56	51	51
A00048	-5170	57	92	81	96	82	96	84	133	97	136
A00048	-5160	95	115	60	133	90	64	137	80	93	135
A00048	-5150	121	178	142	112	121	143	144	95	136	95
A00048	-5140	139	107	184	151	131	107	140	124	194	124
A00048	-5130	123	94	74	82	78	94	999			
A00049	-5320	112	133	177	115	138	102	112	58	73	94
A00049	-5310	100	107	65	112	107	115	98	59	68	73
A00049	-5300	66	89	66	83	45	43	74	74	107	145
A00049	-5290	170	142	150	162	145	138	88	76	93	148
A00049	-5280	136	110	162	175	137	107	124	272	430	367
A00049	-5270	293	273	256	221	171	286	465	287	364	340
A00049	-5260	302	120	141	201	109	113	129	127	128	124
A00049	-5250	135	108	110	155	114	122	136	155	172	132
A00049	-5240	162	100	162	211	149	121	108	89	150	102
A00049	-5230	172	257	143	125	157	217	141	141	166	120
A00049	-5220	151	199	160	210	192	189	168	177	180	96

A00049	-5210	127	145	201	135	170	248	138	129	177	152
A00049	-5200	157	139	92	71	71	86	78	85	107	107
A00049	-5190	94	136	86	76	74	86	92	126	111	136
A00049	-5180	124	93	63	122	107	115	124	100	99	78
A00049	-5170	99	100	122	129	109	126	79	100	82	130
A00049	-5160	80	110	107	100	92	97	140	137	129	132
A00049	-5150	125	153	166	157	128	54	42	53	85	62
A00049	-5140	65	104	165	113	141	75	132	107	137	106
A00049	-5130	99	93	62	54	95	110	999			
A00050	-5211	223									
A00050	-5210	374	391	247	197	218	251	147	217	232	205
A00050	-5200	181	226	137	137	123	150	177	151	156	140
A00050	-5190	215	185	112	140	112	154	123	215	158	151
A00050	-5180	126	112	95	180	129	130	105	96	119	111
A00050	-5170	101	113	92	79	105	103	130	143	138	155
A00050	-5160	130	115	143	116	86	101	144	99	99	84
A00050	-5150	77	126	111	127	111	139	150	132	162	129
A00050	-5140	90	115	136	101	139	133	166	113	109	95
A00050	-5130	98	76	88	95	169	202	999			
A00051	-5207	418	347	310	129	115	116	122			
A00051	-5200	127	132	149	197	167	154	168	165	137	126
A00051	-5190	206	189	90	96	93	109	86	138	144	102
A00051	-5180	100	86	76	155	98	96	83	83	116	76
A00051	-5170	85	85	49	68	70	51	87	87	91	93
A00051	-5160	104	89	103	70	81	64	126	99	96	85
A00051	-5150	55	154	113	110	118	129	153	94	182	112
A00051	-5140	161	139	208	149	167	163	146	97	89	95
A00051	-5130	96	64	97	71	174	133	150	999		
A00052	-5202	329	236								
A00052	-5200	234	239	217	241	196	190	237	186	186	190
A00052	-5190	188	204	186	117	150	144	163	210	192	186
A00052	-5180	137	130	115	144	113	96	101	110	110	108
A00052	-5170	92	101	75	86	129	88	113	135	132	123
A00052	-5160	128	142	132	122	90	117	122	117	103	105
A00052	-5150	96	110	113	116	102	108	113	107	57	74
A00052	-5140	52	73	94	92	78	107	196	191	198	174
A00052	-5130	178	134	103	115	165	122	151	999		
A00053	-5189	247	165	134	158	141	127	179	219	179	
A00053	-5180	145	131	114	141	130	122	107	111	124	112
A00053	-5170	78	80	110	101	98	103	135	144	140	128
A00053	-5160	131	129	133	133	97	102	115	95	87	116
A00053	-5150	117	105	122	118	107	128	122	92	118	83
A00053	-5140	84	106	143	92	119	130	138	127	110	120
A00053	-5130	110	92	108	95	149	114	148	999		
A00054	-5193	108	94	101							
A00054	-5190	103	89	100	93	75	61	51	58	39	72
A00054	-5180	63	89	76	87	131	53	44	57	66	51
A00054	-5170	45	62	74	87	66	60	69	68	90	105
A00054	-5160	73	102	65	83	78	74	111	74	75	81
A00054	-5150	108	119	143	135	143	129	116	107	109	94
A00054	-5140	91	123	218	154	163	116	157	192	168	132
A00054	-5130	137	122	103	103	94	121	160	999		
A00055	-5204	195	191	243	231						
A00055	-5200	192	208	158	188	152	184	219	254	182	207
A00055	-5190	153	223	158	131	162	168	138	224	221	188
A00055	-5180	144	116	86	157	123	108	107	104	132	101
A00055	-5170	85	97	101	90	92	79	142	132	128	118
A00055	-5160	127	109	138	96	98	102	122	87	103	76
A00055	-5150	86	103	106	103	95	100	127	91	116	90
A00055	-5140	83	87	121	63	96	103	135	102	96	96
A00055	-5130	91	91	85	78	150	91	144	999		
A00056	-5210	232	216	240	192	230	211	145	135	202	134
A00056	-5200	157	153	129	155	121	151	186	170	115	128
A00056	-5190	165	154	110	107	134	140	108	185	164	145
A00056	-5180	120	95	101	135	118	111	100	104	123	114
A00056	-5170	82	91	93	76	115	100	131	136	138	140
A00056	-5160	144	120	153	112	83	105	120	95	85	92
A00056	-5150	94	90	115	95	79	114	116	112	135	121
A00056	-5140	111	103	161	96	119	115	129	112	129	120
A00056	-5130	111	90	92	94	124	120	164	999		
A00057	-5205	108	128	136	233	189					
A00057	-5200	147	174	114	159	140	162	191	216	176	170
A00057	-5190	212	189	154	137	164	173	130	227	153	144

A00057	-5180	135	112	105	154	125	105	126	89	140	99
A00057	-5170	99	118	104	87	103	103	129	158	190	135
A00057	-5160	148	155	176	103	83	99	140	100	89	86
A00057	-5150	86	117	104	149	126	170	152	145	276	191
A00057	-5140	143	174	221	116	131	156	147	124	119	146
A00057	-5130	119	102	97	93	171	144	148	999		
A00058	-5222	232	142								
A00058	-5220	184	267	198	300	193	188	252	157	146	59
A00058	-5210	102	137	115	90	206	200	100	152	160	149
A00058	-5200	204	120	88	86	73	74	93	114	135	109
A00058	-5190	120	119	78	62	85	57	55	112	91	119
A00058	-5180	87	86	65	74	94	111	118	73	120	100
A00058	-5170	94	103	118	93	105	92	88	104	107	118
A00058	-5160	104	116	95	132	93	131	178	95	104	111
A00058	-5150	120	185	198	195	170	197	228	124	240	163
A00058	-5140	195	200	373	148	147	111	174	124	168	130
A00058	-5130	147	130	111	105	100	119	135	999		
A00059	-5195	198	199	219	181	170					
A00059	-5190	216	200	165	153	148	169	139	179	171	131
A00059	-5180	139	98	96	172	105	113	104	121	123	122
A00059	-5170	155	139	107	111	98	125	113	156	140	159
A00059	-5160	145	136	165	125	96	98	123	106	82	79
A00059	-5150	74	160	171	171	152	210	232	155	173	121
A00059	-5140	120	184	186	151	166	193	139	134	99	109
A00059	-5130	110	82	122	107	163	117	142	999		
A00060	-5209	256	302	187	278	260	160	183	196	206	
A00060	-5200	198	232	182	166	134	155	188	182	128	156
A00060	-5190	167	159	127	111	136	147	127	149	152	140
A00060	-5180	105	84	102	131	97	102	92	96	102	101
A00060	-5170	97	92	89	93	83	72	82	133	114	121
A00060	-5160	120	105	106	100	79	83	101	83	75	74
A00060	-5150	78	93	98	110	83	116	115	70	111	49
A00060	-5140	100	122	158	113	134	117	152	97	110	98
A00060	-5130	104	77	116	124	305	138	219	218	999	
A00061	-5215	157	252	192	176	227					
A00061	-5210	276	216	155	137	208	228	138	219	380	223
A00061	-5200	197	197	162	162	144	175	167	166	169	143
A00061	-5190	187	195	142	138	134	146	137	199	165	175
A00061	-5180	119	113	107	158	106	101	92	99	122	80
A00061	-5170	103	103	88	66	96	94	127	125	131	119
A00061	-5160	131	121	143	109	94	95	141	117	93	89
A00061	-5150	102	112	111	138	121	152	167	146	196	144
A00061	-5140	98	141	135	140	138	151	155	119	124	110
A00061	-5130	119	100	89	87	142	97	128	127	999	
A00062	-5208	273	138	236	208	124	190	195	141		
A00062	-5200	142	162	116	154	124	156	161	170	145	132
A00062	-5190	162	145	105	86	108	120	108	158	138	111
A00062	-5180	122	91	76	109	73	86	70	76	86	82
A00062	-5170	70	111	102	90	91	79	100	120	153	132
A00062	-5160	145	125	121	113	84	78	125	81	79	94
A00062	-5150	76	118	123	124	135	163	157	172	330	219
A00062	-5140	119	162	209	117	144	166	171	132	150	126
A00062	-5130	130	102	123	119	163	171	167	230	999	
A00063	-5210	283	180	209	182	225	191	158	207	286	188
A00063	-5200	145	156	141	148	107	199	182	193	145	151
A00063	-5190	150	173	149	80	117	158	121	217	177	146
A00063	-5180	138	126	112	173	118	132	112	109	135	100
A00063	-5170	93	93	93	104	109	79	137	130	133	115
A00063	-5160	143	104	120	92	94	96	108	103	93	72
A00063	-5150	100	98	82	97	95	109	119	64	108	95
A00063	-5140	76	122	157	82	118	130	123	108	104	113
A00063	-5130	100	85	52	54	110	132	157	190	999	
A00064	-5282	97	123								
A00064	-5280	98	133	196	189	139	142	169	228	219	348
A00064	-5270	191	261	317	413	132	288	403	297	256	234
A00064	-5260	329	109	159	168	115	101	112	107	159	154
A00064	-5250	141	95	114	185	114	140	188	182	200	149
A00064	-5240	248	113	168	274	165	120	138	105	156	110
A00064	-5230	223	221	148	143	104	237	135	154	140	85
A00064	-5220	144	220	133	224	193	168	197	140	123	75
A00064	-5210	65	114	122	94	210	205	91	115	126	147
A00064	-5200	175	113	79	73	93	77	71	111	105	122
A00064	-5190	92	115	62	68	55	74	62	98	95	109

A00064	-5180	94	67	51	97	73	70	101	63	94	73
A00064	-5170	80	93	103	89	79	76	77	83	112	95
A00064	-5160	91	102	84	106	94	75	159	94	118	116
A00064	-5150	92	151	142	168	191	164	196	123	182	128
A00064	-5140	149	217	299	183	184	121	186	145	207	144
A00064	-5130	157	147	120	110	148	136	143	166	999	
A00065	-5318	150	101	94	111	104	56	72	77		
A00065	-5310	108	109	86	104	101	96	83	68	65	77
A00065	-5300	66	95	106	92	49	45	89	104	124	142
A00065	-5290	142	141	171	122	143	156	74	79	103	129
A00065	-5280	130	139	215	187	153	132	181	306	289	420
A00065	-5270	195	296	276	294	164	245	320	338	297	246
A00065	-5260	232	116	121	165	122	147	124	100	152	133
A00065	-5250	159	97	111	144	96	113	142	119	159	100
A00065	-5240	151	74	178	237	190	125	113	99	156	113
A00065	-5230	215	262	145	138	106	219	152	167	156	125
A00065	-5220	128	219	143	211	222	170	214	174	153	113
A00065	-5210	114	131	137	132	255	276	129	158	153	183
A00065	-5200	300	146	163	151	128	79	98	112	116	120
A00065	-5190	88	110	61	61	60	66	50	75	80	93
A00065	-5180	88	67	69	83	73	97	96	75	79	74
A00065	-5170	86	96	141	99	104	123	90	118	115	138
A00065	-5160	112	134	101	94	87	79	155	106	145	131
A00065	-5150	121	205	154	202	181	91	99	69	99	67
A00065	-5140	93	120	278	197	163	123	177	161	167	125
A00065	-5130	139	140	100	85	71	75	146	168	999	
A00066	-5276	163	65	167	87	61	156				
A00066	-5270	154	339	294	264	131	227	269	109	146	115
A00066	-5260	157	96	95	122	107	67	63	57	69	57
A00066	-5250	56	56	45	52	55	63	65	66	103	54
A00066	-5240	62	40	64	94	63	47	40	34	68	45
A00066	-5230	63	85	55	63	40	66	54	67	74	60
A00066	-5220	92	172	114	112	115	84	93	81	73	46
A00066	-5210	65	89	71	58	87	94	53	74	102	89
A00066	-5200	87	59	41	44	53	50	57	67	72	80
A00066	-5190	79	76	54	53	50	56	46	84	75	79
A00066	-5180	72	53	40	66	55	57	67	66	83	57
A00066	-5170	62	78	85	78	61	59	59	65	64	72
A00066	-5160	70	78	61	65	52	65	105	65	68	80
A00066	-5150	72	97	129	94	93	143	126	92	127	86
A00066	-5140	88	116	185	103	118	83	101	81	101	72
A00066	-5130	55	63	61	68	54	40	76	82	85	999
A00067	-5223	128	125	127							
A00067	-5220	203	246	183	273	228	210	285	156	177	116
A00067	-5210	132	151	180	121	153	172	110	117	119	127
A00067	-5200	146	75	74	82	94	58	103	124	103	125
A00067	-5190	85	117	78	57	79	70	57	95	105	98
A00067	-5180	114	83	63	75	67	99	121	75	115	102
A00067	-5170	117	125	126	111	111	105	109	90	131	128
A00067	-5160	99	83	118	116	78	81	160	87	108	137
A00067	-5150	93	129	149	147	125	213	301	137	237	311
A00067	-5140	179	201	383	235	214	127	200	155	241	161
A00067	-5130	157	138	164	162	213	161	123	189	146	999
A00068	-5264	307	205	166	193						
A00068	-5260	247	104	118	175	86	105	109	113	125	104
A00068	-5250	123	92	96	107	95	110	144	133	180	107
A00068	-5240	157	108	117	185	120	103	105	96	160	140
A00068	-5230	197	270	148	143	78	150	139	172	134	92
A00068	-5220	156	190	145	211	195	157	165	122	124	107
A00068	-5210	104	146	178	129	166	183	104	110	128	142
A00068	-5200	155	75	63	98	100	67	92	115	105	89
A00068	-5190	98	91	57	73	71	42	39	74	54	69
A00068	-5180	50	52	61	51	62	54	90	44	67	69
A00068	-5170	63	77	79	94	67	59	74	95	94	96
A00068	-5160	87	94	76	84	78	77	108	66	82	98
A00068	-5150	92	117	118	96	109	107	115	81	132	98
A00068	-5140	90	141	234	161	160	114	157	133	148	113
A00068	-5130	131	86	83	73	63	55	112	108	128	999
A00069	-5217	125	172	132	165	136	153	182			
A00069	-5210	275	252	177	97	161	171	114	238	346	209
A00069	-5200	143	165	126	158	125	148	136	170	130	127
A00069	-5190	183	185	132	116	103	138	121	156	163	140
A00069	-5180	126	78	86	142	94	75	85	96	102	81

A00069	-5170	87	101	90	69	87	76	119	128	126	112
A00069	-5160	135	95	118	92	91	73	126	88	89	71
A00069	-5150	79	115	94	102	99	142	132	115	175	145
A00069	-5140	79	95	145	108	134	98	126	111	104	92
A00069	-5130	107	85	82	99	117	96	113	135	188	999
A00070	-5343	171	130	91							
A00070	-5340	69	90	121	109	132	170	198	108	168	106
A00070	-5330	278	286	226	167	195	152	81	147	124	91
A00070	-5320	105	141	165	114	133	133	113	62	68	97
A00070	-5310	114	101	79	117	82	121	68	64	75	73
A00070	-5300	73	82	93	94	43	45	73	101	102	131
A00070	-5290	148	123	98	118	114	95	69	75	84	162
A00070	-5280	147	169	213	148	181	165	222	246	240	288
A00070	-5270	258	275	214	278	159	264	277	313	257	245
A00070	-5260	326	116	161	176	101	122	98	133	130	114
A00070	-5250	117	110	128	160	105	138	199	177	181	162
A00070	-5240	230	108	158	228	165	141	126	156	230	147
A00070	-5230	210	224	135	131	122	189	150	165	168	122
A00070	-5220	210	274	176	194	192	159	159	179	134	103
A00070	-5210	132	99	105	93	183	265	108	201	139	115
A00070	-5200	234	138	106	133	146	90	118	106	147	92
A00070	-5190	134	103	83	62	78	67	60	106	87	91
A00070	-5180	96	104	59	91	78	82	71	70	70	85
A00070	-5170	85	109	127	96	92	101	84	86	121	131
A00070	-5160	133	113	108	122	72	73	189	104	119	146
A00070	-5150	139	182	223	193	186	122	127	88	98	100
A00070	-5140	164	74	127	97	128	85	98	87	134	85
A00070	-5130	110	71	71	94	96	111	108	119	67	94
A00070	-5120	108	999								
A00071	-5202	180	191								
A00071	-5200	169	185	150	174	119	159	177	224	111	133
A00071	-5190	171	175	144	135	130	163	156	250	183	132
A00071	-5180	119	102	105	147	101	91	86	97	128	92
A00071	-5170	86	95	85	78	111	84	135	167	135	132
A00071	-5160	163	159	170	105	88	108	145	115	96	85
A00071	-5150	94	118	126	112	110	139	132	103	183	144
A00071	-5140	88	142	176	111	134	136	175	122	129	135
A00071	-5130	119	96	114	94	221	209	264	296	330	213
A00071	-5120	192	999								
A00072	-5359	114	164	117	157	167	162	137	105	228	
A00072	-5350	191	145	135	129	93	139	116	160	111	87
A00072	-5340	83	95	126	105	105	127	115	91	134	105
A00072	-5330	231	164	203	97	175	119	93	140	108	100
A00072	-5320	90	122	167	118	111	101	103	45	65	111
A00072	-5310	102	74	69	106	102	81	71	57	70	78
A00072	-5300	60	61	78	68	39	29	38	62	95	114
A00072	-5290	95	76	125	115	89	82	52	52	67	120
A00072	-5280	83	146	203	164	106	99	159	202	257	316
A00072	-5270	243	359	259	309	163	294	356	367	296	214
A00072	-5260	304	89	126	191	106	119	83	92	108	105
A00072	-5250	108	104	129	158	117	132	146	197	185	188
A00072	-5240	246	129	228	254	207	155	150	140	234	147
A00072	-5230	249	282	170	127	113	190	166	146	178	137
A00072	-5220	296	431	278	352	290	186	197	127	108	95
A00072	-5210	115	74	100	86	206	192	102	172	140	142
A00072	-5200	161	119	106	101	112	95	100	94	113	99
A00072	-5190	89	82	60	43	58	52	30	56	44	86
A00072	-5180	71	70	79	116	59	58	54	48	52	40
A00072	-5170	62	68	93	72	85	72	69	96	107	114
A00072	-5160	87	98	77	77	79	60	166	105	85	99
A00072	-5150	113	139	201	145	146	127	126	89	111	99
A00072	-5140	87	73	171	106	121	83	117	89	137	97
A00072	-5130	114	92	97	100	101	95	115	127	77	96
A00072	-5120	115	999								
A00073	-5263	258	232	233							
A00073	-5260	261	126	189	226	160	209	204	193	183	179
A00073	-5250	182	191	197	197	125	148	155	179	209	147
A00073	-5240	221	140	181	266	197	126	122	125	166	123
A00073	-5230	219	290	108	124	121	191	163	205	206	136
A00073	-5220	222	246	127	195	186	129	164	117	113	86
A00073	-5210	117	98	147	87	168	148	102	154	122	120
A00073	-5200	158	110	88	98	113	98	104	106	111	96
A00073	-5190	104	110	76	64	68	66	51	54	53	82

A00073	-5180	79	77	73	59	59	71	89	52	88	83
A00073	-5170	99	110	140	111	99	80	79	92	93	123
A00073	-5160	81	92	90	85	70	92	118	73	78	87
A00073	-5150	98	94	140	110	124	133	128	90	132	86
A00073	-5140	136	168	212	190	170	115	149	129	162	123
A00073	-5130	139	92	106	87	74	96	130	137	103	82
A00073	-5120	117	999								
A00074	-5311	122									
A00074	-5310	106	108	90	94	97	128	75	59	101	102
A00074	-5300	64	75	73	106	39	38	140	126	140	172
A00074	-5290	211	152	192	220	182	129	42	59	68	133
A00074	-5280	106	133	211	213	144	128	213	281	223	361
A00074	-5270	188	236	211	300	127	300	566	252	322	202
A00074	-5260	284	99	167	205	132	144	128	123	185	186
A00074	-5250	147	94	124	249	134	176	252	175	222	212
A00074	-5240	283	157	327	357	285	202	193	167	246	148
A00074	-5230	367	326	189	214	185	333	255	299	233	214
A00074	-5220	207	376	191	222	266	201	221	206	144	87
A00074	-5210	122	140	173	153	243	330	156	204	247	186
A00074	-5200	267	141	95	119	150	94	130	154	235	128
A00074	-5190	135	152	75	79	102	88	68	100	96	108
A00074	-5180	101	77	70	79	68	65	95	70	87	78
A00074	-5170	84	83	144	92	97	104	107	111	167	129
A00074	-5160	101	122	104	113	93	102	201	128	160	162
A00074	-5150	189	300	230	264	241	122	114	76	166	107
A00074	-5140	128	219	413	253	149	111	210	263	220	138
A00074	-5130	157	138	83	76	91	116	261	226	133	101
A00074	-5120	221	147	999							
A00075	-5191	204									
A00075	-5190	148	170	170	59	77	94	91	83	147	115
A00075	-5180	254	75	61	125	94	112	102	86	94	61
A00075	-5170	101	98	153	131	119	98	92	122	125	183
A00075	-5160	123	135	110	164	144	87	254	149	167	207
A00075	-5150	186	428	264	281	345	240	257	165	271	280
A00075	-5140	240	166	337	108	168	104	226	230	272	143
A00075	-5130	194	124	81	108	164	174	135	146	122	112
A00075	-5120	241	227	999							
A00076	-5279	184	235	270	206	200	219	240	306	365	
A00076	-5270	254	295	235	310	173	273	508	388	308	235
A00076	-5260	270	135	182	251	136	111	110	130	171	204
A00076	-5250	163	103	93	131	99	103	167	121	180	140
A00076	-5240	151	114	189	209	129	80	117	112	131	113
A00076	-5230	214	238	165	117	136	168	121	113	151	113
A00076	-5220	201	225	260	265	219	145	217	272	196	160
A00076	-5210	142	200	152	100	161	155	94	115	134	135
A00076	-5200	119	69	65	46	71	60	79	92	85	94
A00076	-5190	84	93	54	69	67	50	59	109	80	88
A00076	-5180	114	82	51	109	98	122	98	116	107	85
A00076	-5170	91	117	125	136	100	72	81	80	86	107
A00076	-5160	101	96	78	87	53	53	101	100	75	101
A00076	-5150	103	104	127	95	117	117	96	94	120	119
A00076	-5140	121	144	283	135	135	108	155	123	157	112
A00076	-5130	92	92	105	105	119	110	181	174	108	91
A00076	-5120	102	52	124	999						
A00077	-5352	73	119								
A00077	-5350	106	90	139	149	110	125	126	159	117	93
A00077	-5340	105	125	127	136	114	125	118	87	127	72
A00077	-5330	290	151	156	120	196	110	65	111	91	70
A00077	-5320	80	109	173	104	86	71	78	33	96	126
A00077	-5310	110	94	96	108	101	112	90	70	102	113
A00077	-5300	98	68	94	70	56	47	108	130	135	176
A00077	-5290	175	155	167	130	160	116	67	76	109	92
A00077	-5280	105	150	179	146	149	116	202	211	229	345
A00077	-5270	213	316	304	278	148	254	410	252	326	215
A00077	-5260	241	82	137	196	134	123	143	127	201	170
A00077	-5250	201	111	116	146	105	110	135	117	164	136
A00077	-5240	144	85	117	189	133	91	104	79	132	107
A00077	-5230	197	227	123	140	110	217	155	158	204	126
A00077	-5220	198	292	155	202	206	141	182	157	138	86
A00077	-5210	97	86	111	97	197	183	103	121	159	176
A00077	-5200	177	127	91	111	118	87	134	93	117	131
A00077	-5190	105	133	65	58	61	52	49	73	73	81
A00077	-5180	69	66	49	77	55	80	87	84	98	64

A00077	-5170	62	88	91	88	109	88	88	70	76	92
A00077	-5160	64	82	55	99	75	67	118	97	87	107
A00077	-5150	99	109	133	106	95	61	85	78	114	93
A00077	-5140	91	111	209	102	138	99	137	115	173	105
A00077	-5130	95	104	111	84	88	96	128	109	95	96
A00077	-5120	113	143	177	999						
A00078	-5207	99	213	163	126	168	186	143			
A00078	-5200	158	180	136	129	128	167	165	143	123	124
A00078	-5190	155	155	125	120	114	111	105	146	154	97
A00078	-5180	103	86	92	131	94	90	83	76	108	70
A00078	-5170	76	85	88	73	104	69	119	112	112	126
A00078	-5160	114	113	101	110	63	94	117	98	82	95
A00078	-5150	86	105	89	107	115	127	156	100	126	70
A00078	-5140	85	99	120	96	112	121	120	81	84	92
A00078	-5130	115	92	110	84	117	86	111	115	183	135
A00078	-5120	128	117	123	999						
A00079	-5318	181	82	97	116	119	57	98	129		
A00079	-5310	128	109	85	98	142	121	70	79	86	131
A00079	-5300	97	102	135	116	56	51	119	119	135	137
A00079	-5290	177	199	189	156	171	159	72	90	118	181
A00079	-5280	131	136	250	183	150	117	165	285	219	411
A00079	-5270	187	419	354	285	152	319	452	416	325	274
A00079	-5260	280	106	139	214	145	164	166	145	212	203
A00079	-5250	221	116	133	182	126	130	162	143	210	140
A00079	-5240	230	133	166	300	217	132	141	112	159	145
A00079	-5230	255	291	145	134	134	214	169	188	211	129
A00079	-5220	191	241	165	222	232	198	160	154	127	85
A00079	-5210	101	96	94	80	194	225	95	117	141	129
A00079	-5200	220	121	104	151	143	91	115	119	133	123
A00079	-5190	111	134	76	59	66	73	51	84	68	110
A00079	-5180	94	77	44	84	67	111	112	105	125	83
A00079	-5170	98	90	130	102	130	106	83	99	88	118
A00079	-5160	92	98	77	126	72	94	177	126	111	132
A00079	-5150	96	164	136	152	192	113	209	105	173	114
A00079	-5140	132	154	346	145	159	102	175	153	176	137
A00079	-5130	121	102	127	114	119	128	175	188	116	96
A00079	-5120	137	172	189	153	999					
A00080	-5334	179	100	168	113						
A00080	-5330	328	239	155	134	289	166	90	133	160	99
A00080	-5320	87	193	180	104	114	81	84	69	103	105
A00080	-5310	122	98	87	108	98	111	72	72	63	76
A00080	-5300	79	68	73	87	52	52	108	100	135	154
A00080	-5290	160	149	139	141	162	125	65	69	97	130
A00080	-5280	107	144	215	167	152	107	190	208	208	352
A00080	-5270	129	279	296	269	128	258	398	165	292	302
A00080	-5260	271	120	184	176	137	111	155	121	183	198
A00080	-5250	199	74	132	188	118	135	161	185	176	140
A00080	-5240	208	133	182	254	166	109	92	97	149	115
A00080	-5230	177	234	146	143	125	274	194	208	190	119
A00080	-5220	177	209	118	195	205	198	185	170	78	60
A00080	-5210	74	82	111	87	254	219	84	115	127	108
A00080	-5200	147	85	51	80	95	101	107	107	96	94
A00080	-5190	112	111	62	73	45	66	68	89	73	117
A00080	-5180	98	86	55	95	91	111	109	78	107	88
A00080	-5170	73	88	100	92	96	98	100	87	102	115
A00080	-5160	81	102	64	116	71	70	153	94	102	116
A00080	-5150	101	129	128	130	132	106	148	125	141	110
A00080	-5140	111	136	234	95	120	85	155	120	156	124
A00080	-5130	133	93	111	102	99	100	123	150	131	84
A00080	-5120	122	170	129	122	999					
A00081	-5322	60	42								
A00081	-5320	54	105	123	63	69	60	70	47	68	108
A00081	-5310	103	76	71	83	75	108	66	58	48	83
A00081	-5300	77	80	102	126	60	62	102	83	65	72
A00081	-5290	102	104	85	114	79	149	66	51	48	77
A00081	-5280	77	85	139	113	129	143	199	168	350	529
A00081	-5270	287	277	305	398	236	296	409	427	264	221
A00081	-5260	275	128	166	169	150	137	107	114	144	135
A00081	-5250	95	77	58	115	97	97	125	117	156	108
A00081	-5240	131	100	146	193	171	114	108	114	144	123
A00081	-5230	162	267	151	121	113	212	160	171	194	155
A00081	-5220	186	243	161	262	194	163	224	172	184	120
A00081	-5210	163	172	187	151	197	220	127	152	128	124

A00081	-5200	125	68	59	45	66	76	93	108	92	107
A00081	-5190	69	81	75	68	60	62	72	124	100	114
A00081	-5180	90	108	67	124	128	194	95	139	99	74
A00081	-5170	84	112	86	124	102	110	130	132	160	151
A00081	-5160	120	121	105	135	83	86	164	112	157	192
A00081	-5150	185	156	177	193	167	397	608	251	442	536
A00081	-5140	410	262	585	278	328	199	282	236	284	194
A00081	-5130	229	169	161	219	291	212	223	195	139	149
A00081	-5120	253	281	337	121	999					
A00082	-5264	470	313	394	315						
A00082	-5260	469	114	271	296	187	100	149	171	290	264
A00082	-5250	289	122	115	185	124	146	244	156	246	224
A00082	-5240	297	133	286	286	169	137	149	124	220	162
A00082	-5230	267	335	152	225	197	370	193	264	245	187
A00082	-5220	165	338	176	197	199	218	180	153	104	60
A00082	-5210	107	115	124	97	270	218	111	198	224	178
A00082	-5200	240	117	88	149	139	79	111	159	129	113
A00082	-5190	130	208	50	74	87	70	99	121	122	115
A00082	-5180	79	111	101	131	66	68	104	74	99	74
A00082	-5170	80	104	127	113	110	105	97	98	137	129
A00082	-5160	84	104	94	109	111	83	198	119	130	114
A00082	-5150	117	214	160	171	186	170	246	150	221	181
A00082	-5140	181	240	402	160	163	102	314	252	307	205
A00082	-5130	246	157	156	141	211	208	211	207	145	129
A00082	-5120	321	175	234	174	999					
A00083	-5203	287	399	276							
A00083	-5200	199	220	99	135	124	128	179	175	150	133
A00083	-5190	155	158	105	92	93	125	125	182	151	137
A00083	-5180	114	96	89	144	97	97	91	83	112	80
A00083	-5170	90	85	74	73	78	80	94	115	103	97
A00083	-5160	115	95	112	104	66	88	112	115	103	91
A00083	-5150	87	125	124	107	109	107	107	81	111	103
A00083	-5140	90	100	107	90	95	106	120	103	117	120
A00083	-5130	100	78	89	65	111	92	96	121	115	93
A00083	-5120	91	69	87	122	999					
A00084	-5272	182	296								
A00084	-5270	203	292	319	290	170	255	307	325	332	308
A00084	-5260	298	132	153	223	108	163	227	212	199	214
A00084	-5250	168	161	153	159	103	113	157	161	159	132
A00084	-5240	200	113	179	220	188	111	123	106	154	119
A00084	-5230	211	260	134	120	90	189	160	177	197	167
A00084	-5220	211	273	129	207	186	138	118	127	76	104
A00084	-5210	105	109	106	95	142	147	111	135	119	141
A00084	-5200	160	102	107	94	102	71	106	102	126	105
A00084	-5190	94	116	69	57	67	48	52	56	57	84
A00084	-5180	68	67	64	52	45	58	61	66	77	88
A00084	-5170	83	101	103	82	74	79	72	95	82	79
A00084	-5160	111	93	84	69	62	70	125	57	64	95
A00084	-5150	86	130	173	106	143	120	119	59	126	88
A00084	-5140	145	163	203	145	170	125	127	138	112	121
A00084	-5130	110	110	108	85	64	81	125	118	102	75
A00084	-5120	99	106	96	145	999					
A00085	-5328	125	61	158	154	77	125	145	84		
A00085	-5320	93	139	197	127	121	98	109	60	89	139
A00085	-5310	141	117	115	151	138	121	74	64	115	151
A00085	-5300	119	100	124	114	59	57	97	93	128	187
A00085	-5290	150	134	104	115	143	135	56	87	79	139
A00085	-5280	86	113	183	171	153	213	611	552	477	811
A00085	-5270	256	248	212	195	87	205	333	292	249	164
A00085	-5260	184	76	107	119	88	126	117	115	148	169
A00085	-5250	144	107	125	137	77	118	150	100	149	79
A00085	-5240	145	89	118	191	136	79	97	98	112	95
A00085	-5230	144	170	118	138	113	191	167	176	182	115
A00085	-5220	199	262	138	208	196	171	173	126	117	111
A00085	-5210	92	103	104	92	150	155	110	115	124	156
A00085	-5200	135	94	95	98	125	92	108	101	133	147
A00085	-5190	124	124	79	64	72	70	43	78	66	111
A00085	-5180	76	86	59	62	89	94	114	75	103	94
A00085	-5170	102	114	123	103	85	94	81	70	92	120
A00085	-5160	95	99	90	104	80	91	152	114	121	134
A00085	-5150	113	124	153	144	126	117	128	96	144	95
A00085	-5140	151	188	250	160	182	115	158	119	155	123
A00085	-5130	128	88	91	86	101	100	129	140	105	96

A00085	-5120	108	125	125	104	999					
A00086	-5194	117	137	95	127						
A00086	-5190	104	99	79	75	81	111	101	141	85	123
A00086	-5180	98	100	86	136	101	105	99	81	125	94
A00086	-5170	86	99	150	109	119	95	94	105	130	141
A00086	-5160	123	136	126	119	118	96	201	143	131	157
A00086	-5150	146	263	189	172	220	66	96	95	116	94
A00086	-5140	112	119	207	125	160	67	148	166	240	146
A00086	-5130	117	118	105	85	129	124	136	178	118	106
A00086	-5120	153	129	133	122	999					
A00087	-5320	95	114	141	85	120	98	116	54	93	126
A00087	-5310	131	90	111	94	118	111	78	74	105	102
A00087	-5300	101	80	81	71	56	38	80	93	103	129
A00087	-5290	153	148	135	140	92	119	51	62	79	98
A00087	-5280	117	164	219	205	185	172	186	235	240	368
A00087	-5270	233	217	326	335	187	326	459	375	377	294
A00087	-5260	367	112	176	222	126	110	105	104	187	158
A00087	-5250	127	98	115	177	104	144	179	198	164	150
A00087	-5240	288	116	224	262	195	134	123	123	167	111
A00087	-5230	264	264	136	149	121	215	151	180	156	121
A00087	-5220	126	199	135	229	196	196	232	126	123	84
A00087	-5210	94	117	96	104	165	199	111	124	153	117
A00087	-5200	173	95	68	66	83	59	105	128	126	129
A00087	-5190	129	156	69	84	72	72	78	111	113	120
A00087	-5180	92	73	84	79	81	104	88	83	97	73
A00087	-5170	95	90	115	112	82	82	88	97	110	128
A00087	-5160	90	91	83	100	74	87	163	76	134	119
A00087	-5150	97	150	163	130	147	184	198	110	208	147
A00087	-5140	205	171	372	225	264	161	292	258	297	160
A00087	-5130	248	131	115	107	129	163	204	163	128	83
A00087	-5120	217	196	251	134	256	999				
A00088	-5366	108	108	144	113	122	181				
A00088	-5360	176	121	227	126	201	172	130	99	78	91
A00088	-5350	135	112	180	197	126	146	126	281	234	91
A00088	-5340	91	110	127	135	121	167	135	104	161	115
A00088	-5330	287	231	152	131	266	141	95	162	157	83
A00088	-5320	92	138	212	87	88	93	129	55	91	131
A00088	-5310	127	89	91	114	142	131	74	88	91	110
A00088	-5300	95	103	133	150	58	55	118	127	120	141
A00088	-5290	156	172	165	146	216	190	71	66	100	152
A00088	-5280	101	126	188	163	146	109	173	242	220	402
A00088	-5270	202	402	363	258	133	302	432	353	273	276
A00088	-5260	239	91	128	175	147	134	163	135	207	189
A00088	-5250	221	105	144	188	122	134	183	150	185	112
A00088	-5240	193	126	175	266	209	124	109	135	159	134
A00088	-5230	254	303	123	139	135	235	209	191	233	147
A00088	-5220	215	236	156	285	246	189	178	166	123	89
A00088	-5210	106	113	119	94	231	236	96	120	141	146
A00088	-5200	209	126	87	122	130	91	91	119	109	97
A00088	-5190	129	147	79	66	80	72	47	88	75	94
A00088	-5180	100	75	55	75	93	100	105	88	113	76
A00088	-5170	96	95	121	90	98	102	97	87	90	109
A00088	-5160	73	127	62	126	98	72	147	126	104	133
A00088	-5150	99	131	137	127	162	95	211	104	153	119
A00088	-5140	119	132	268	156	134	106	175	119	155	114
A00088	-5130	110	84	120	93	100	133	146	152	103	85
A00088	-5120	146	136	156	123	184	999				
A00089	-5310	104	83	95	135	138	106	84	71	83	113
A00089	-5300	110	111	122	109	58	55	86	104	91	114
A00089	-5290	147	101	143	113	71	89	52	81	91	88
A00089	-5280	87	107	178	111	111	107	122	161	157	366
A00089	-5270	224	214	205	218	148	231	343	265	319	239
A00089	-5260	305	95	146	179	89	95	134	90	132	114
A00089	-5250	101	94	89	136	91	115	133	167	160	101
A00089	-5240	153	83	162	197	135	99	104	101	155	114
A00089	-5230	181	223	159	145	105	239	128	131	166	87
A00089	-5220	126	200	156	159	216	155	232	188	166	64
A00089	-5210	92	119	133	113	222	206	105	119	138	159
A00089	-5200	153	104	79	78	82	91	78	100	93	120
A00089	-5190	115	103	68	77	52	59	65	91	99	105
A00089	-5180	128	74	56	74	101	137	122	78	95	93
A00089	-5170	111	74	115	98	100	92	68	101	107	109
A00089	-5160	98	98	88	101	71	96	105	96	94	95

A00089	-5150	99	104	127	118	135	105	127	107	146	105
A00089	-5140	124	167	206	162	150	119	109	112	156	123
A00089	-5130	127	88	100	104	101	64	132	149	106	80
A00089	-5120	126	148	173	183	198	999				
A00090	-5256	155	163	157	182	227	218				
A00090	-5250	217	110	176	196	116	148	169	184	212	180
A00090	-5240	167	122	186	256	190	140	153	115	147	117
A00090	-5230	188	290	117	126	102	191	158	178	156	166
A00090	-5220	196	240	130	175	197	137	111	112	98	67
A00090	-5210	84	94	101	82	107	165	80	120	115	110
A00090	-5200	115	98	91	104	97	92	107	92	101	86
A00090	-5190	83	89	70	65	57	61	47	67	61	70
A00090	-5180	61	53	53	73	75	63	79	63	90	61
A00090	-5170	70	87	93	83	68	76	70	78	80	100
A00090	-5160	89	91	79	91	67	68	166	87	92	101
A00090	-5150	79	105	130	103	123	92	107	99	155	93
A00090	-5140	124	119	196	105	140	94	129	125	115	88
A00090	-5130	96	96	103	98	83	84	112	105	79	73
A00090	-5120	84	105	121	100	158	999				
A00091	-5211	102									
A00091	-5210	104	164	125	125	134	158	102	170	222	131
A00091	-5200	142	149	111	132	91	134	136	173	130	99
A00091	-5190	160	147	99	117	102	115	91	138	111	125
A00091	-5180	85	87	85	114	91	80	74	69	87	63
A00091	-5170	69	69	60	49	87	64	83	103	85	119
A00091	-5160	107	111	97	77	85	78	99	69	72	74
A00091	-5150	73	91	90	100	96	109	130	124	199	108
A00091	-5140	78	121	115	73	76	86	103	83	57	74
A00091	-5130	59	66	82	45	67	90	70	61	83	88
A00091	-5120	80	59	68	56	47	70	999			
A00092	-5278	223	254	188	185	213	248	294	386		
A00092	-5270	271	324	271	328	181	231	475	535	456	341
A00092	-5260	401	249	174	309	174	156	158	128	159	203
A00092	-5250	144	103	81	98	78	112	152	159	172	167
A00092	-5240	136	121	192	211	172	110	117	107	126	109
A00092	-5230	175	298	146	134	118	140	130	116	92	94
A00092	-5220	133	228	229	256	190	132	262	208	187	132
A00092	-5210	137	134	163	92	125	183	117	100	155	135
A00092	-5200	134	73	42	42	52	43	63	61	75	70
A00092	-5190	79	77	59	44	61	58	40	66	83	89
A00092	-5180	78	64	50	86	71	95	79	86	73	62
A00092	-5170	70	76	90	85	76	76	73	115	78	111
A00092	-5160	92	93	71	69	62	45	100	79	88	77
A00092	-5150	80	95	106	78	84	145	139	84	105	93
A00092	-5140	101	141	251	123	103	82	130	109	121	90
A00092	-5130	83	83	80	96	109	88	141	131	106	80
A00092	-5120	106	138	139	87	152	128	99	999		
A00093	-5189	815	284	188	252	348	233	387	277	196	
A00093	-5180	181	98	101	178	74	105	90	73	98	73
A00093	-5170	82	96	68	49	85	58	107	107	123	81
A00093	-5160	128	75	114	85	69	70	126	79	95	75
A00093	-5150	79	150	110	94	88	92	98	86	118	91
A00093	-5140	73	93	99	95	65	101	99	82	80	80
A00093	-5130	82	118	175	87	83	125	122	96	112	63
A00093	-5120	83	79	83	82	124	83	100	999		
A00094	-5323	134	106	87							
A00094	-5320	79	118	116	80	96	104	137	63	103	123
A00094	-5310	126	100	115	103	134	96	67	71	102	101
A00094	-5300	101	107	112	105	44	59	114	105	117	136
A00094	-5290	198	229	201	164	165	161	81	81	112	143
A00094	-5280	142	164	246	144	145	194	228	308	266	416
A00094	-5270	182	188	246	257	147	264	345	285	220	177
A00094	-5260	167	82	102	114	86	91	86	114	120	91
A00094	-5250	93	91	90	191	105	135	157	180	192	120
A00094	-5240	178	123	178	239	170	99	120	117	172	91
A00094	-5230	220	238	143	123	120	196	135	108	112	105
A00094	-5220	115	205	176	260	130	141	191	107	117	79
A00094	-5210	107	155	113	88	114	187	87	89	125	124
A00094	-5200	128	87	43	36	54	52	69	68	79	87
A00094	-5190	83	101	43	62	59	50	48	80	86	114
A00094	-5180	96	102	71	89	89	94	83	58	78	71
A00094	-5170	81	86	95	110	86	75	87	96	101	116
A00094	-5160	102	95	70	94	68	70	112	83	87	82

A00094	-5150	69	94	109	87	97	149	121	80	93	102
A00094	-5140	108	105	201	142	141	110	176	133	147	123
A00094	-5130	159	119	128	109	162	131	157	130	132	110
A00094	-5120	162	155	239	156	255	135	130	999		
A00095	-5278	135	143	169	165	232	249	323	429		
A00095	-5270	248	205	312	244	152	265	515	417	373	265
A00095	-5260	238	125	158	177	136	115	107	131	190	151
A00095	-5250	124	57	122	163	89	106	148	145	156	153
A00095	-5240	145	127	167	206	168	115	145	81	145	89
A00095	-5230	205	251	145	150	164	247	147	116	132	110
A00095	-5220	157	328	194	313	206	227	254	197	181	141
A00095	-5210	182	98	95	85	199	256	110	154	215	167
A00095	-5200	222	111	112	135	146	90	116	133	174	124
A00095	-5190	131	134	72	67	72	70	75	122	96	101
A00095	-5180	98	75	69	100	90	77	89	76	91	66
A00095	-5170	101	82	120	105	97	91	81	112	97	121
A00095	-5160	103	117	84	129	99	77	155	114	120	109
A00095	-5150	112	165	138	133	177	122	177	145	198	239
A00095	-5140	220	157	307	213	187	107	163	168	249	167
A00095	-5130	143	110	107	149	155	141	148	175	102	97
A00095	-5120	259	211	271	154	247	163	206	209	999	
A00096	-5206	134	245	139	131	204	154				
A00096	-5200	158	135	138	154	119	166	231	229	134	131
A00096	-5190	195	207	135	119	120	121	134	207	211	218
A00096	-5180	164	111	113	147	75	107	129	187	279	278
A00096	-5170	165	180	232	160	146	148	232	217	270	273
A00096	-5160	283	155	214	144	119	133	149	129	114	112
A00096	-5150	112	143	152	124	153	181	193	162	268	201
A00096	-5140	160	145	257	183	194	220	208	129	123	126
A00096	-5130	146	109	157	111	153	141	188	127	122	127
A00096	-5120	132	123	156	191	189	137	168	145	999	
A00097	-5194	180	203	152	173						
A00097	-5190	146	183	149	112	137	146	118	184	174	161
A00097	-5180	113	100	89	128	114	92	110	110	111	107
A00097	-5170	77	80	91	94	96	86	115	116	114	127
A00097	-5160	101	119	125	97	91	85	103	105	87	109
A00097	-5150	89	86	112	91	93	96	98	85	73	73
A00097	-5140	79	97	114	87	107	108	104	93	98	119
A00097	-5130	97	74	85	97	115	104	107	153	187	147
A00097	-5120	131	105	136	157	163	164	250	189	163	999
A00098	-5199	166	142	181	124	114	204	158	156	131	
A00098	-5190	290	259	148	159	147	139	133	183	207	133
A00098	-5180	134	100	91	162	106	97	99	87	114	87
A00098	-5170	75	81	63	70	67	66	86	105	97	108
A00098	-5160	106	99	100	98	64	73	93	84	77	92
A00098	-5150	83	101	89	87	92	108	125	89	109	97
A00098	-5140	102	116	125	92	120	115	103	98	104	94
A00098	-5130	103	78	105	75	189	144	145	186	165	141
A00098	-5120	126	105	140	136	121	116	180	148	149	999
A00099	-5307	126	117	126	93	89	93	109			
A00099	-5300	70	84	100	83	48	35	101	117	128	138
A00099	-5290	150	138	116	126	130	150	84	58	46	129
A00099	-5280	122	129	205	185	202	207	280	392	441	577
A00099	-5270	321	258	352	266	154	300	533	403	348	278
A00099	-5260	295	99	175	197	138	128	134	124	167	138
A00099	-5250	137	88	98	158	113	102	164	133	222	130
A00099	-5240	228	95	204	222	151	107	116	86	133	106
A00099	-5230	195	286	139	170	148	336	152	148	143	146
A00099	-5220	194	302	170	366	280	207	289	179	148	86
A00099	-5210	116	107	120	87	231	198	138	154	188	165
A00099	-5200	191	85	79	84	76	71	90	123	102	128
A00099	-5190	88	147	53	73	82	64	70	95	89	115
A00099	-5180	82	67	60	80	88	93	92	72	68	61
A00099	-5170	77	64	75	94	75	96	80	99	112	134
A00099	-5160	86	108	110	118	69	65	178	101	123	122
A00099	-5150	112	141	133	113	104	210	295	135	206	353
A00099	-5140	183	189	365	255	165	150	218	170	169	137
A00099	-5130	137	125	150	177	214	136	150	164	118	124
A00099	-5120	285	183	204	92	251	156	194	155	130	999
A00100	-5267	186	193	197	261	280	190	182			
A00100	-5260	146	101	187	152	94	127	128	92	174	118
A00100	-5250	129	82	91	136	100	126	135	110	143	122

A00100	-5240	139	124	153	222	178	117	121	121	122	108
A00100	-5230	195	261	136	139	130	196	151	125	177	117
A00100	-5220	174	252	203	263	208	178	236	176	160	104
A00100	-5210	105	112	163	117	210	230	113	177	196	177
A00100	-5200	230	103	105	119	95	69	106	114	108	106
A00100	-5190	128	106	75	75	75	63	52	115	80	123
A00100	-5180	87	77	76	59	75	94	109	65	91	91
A00100	-5170	93	73	114	133	103	83	80	103	117	130
A00100	-5160	93	127	111	129	103	104	156	126	117	185
A00100	-5150	171	224	192	171	170	158	159	134	218	194
A00100	-5140	184	257	435	258	244	96	261	176	177	148
A00100	-5130	139	107	76	102	191	164	140	192	128	98
A00100	-5120	177	216	257	111	189	159	167	150	165	999
A00101	-5347	128	98	84	95	122	122	116			
A00101	-5340	153	85	117	103	102	166	127	101	104	108
A00101	-5330	257	143	136	103	166	148	65	109	107	101
A00101	-5320	75	94	154	99	76	63	82	42	45	87
A00101	-5310	120	119	89	95	91	110	84	73	79	100
A00101	-5300	75	86	87	92	50	31	92	96	129	125
A00101	-5290	154	100	122	126	157	147	80	54	80	117
A00101	-5280	99	137	175	176	131	169	166	275	285	407
A00101	-5270	275	246	273	259	133	195	364	328	224	206
A00101	-5260	178	91	109	147	85	111	94	101	133	120
A00101	-5250	117	59	76	114	81	109	121	119	129	142
A00101	-5240	118	94	144	176	130	113	106	113	119	113
A00101	-5230	127	159	137	145	123	202	155	144	163	121
A00101	-5220	174	232	189	301	270	198	225	202	126	88
A00101	-5210	136	166	182	127	210	213	109	146	133	206
A00101	-5200	269	137	140	164	106	75	76	128	118	108
A00101	-5190	117	107	72	61	72	58	50	98	76	99
A00101	-5180	84	62	74	58	57	61	92	77	97	94
A00101	-5170	98	116	162	137	111	111	90	118	116	159
A00101	-5160	112	117	110	134	103	88	155	105	107	135
A00101	-5150	95	211	169	192	194	211	113	102	159	99
A00101	-5140	125	186	378	240	206	140	254	238	228	144
A00101	-5130	202	125	69	98	143	140	174	178	137	98
A00101	-5120	199	187	235	149	259	144	196	84	92	999
A00102	-5327	80	131	121	79	101	96	77			
A00102	-5320	104	112	104	77	92	65	70	50	70	89
A00102	-5310	75	65	58	71	77	92	61	54	43	66
A00102	-5300	70	78	75	74	45	42	61	73	95	118
A00102	-5290	111	120	95	122	194	98	57	58	76	90
A00102	-5280	76	81	118	137	84	108	113	204	244	295
A00102	-5270	157	201	159	154	119	152	277	221	309	270
A00102	-5260	255	163	205	268	116	111	150	90	122	125
A00102	-5250	106	84	84	118	86	125	132	130	170	121
A00102	-5240	143	89	122	177	112	94	98	105	152	121
A00102	-5230	189	266	149	160	120	200	150	176	174	100
A00102	-5220	199	227	153	234	243	246	185	137	163	114
A00102	-5210	170	211	193	128	189	210	124	155	148	186
A00102	-5200	169	95	101	115	102	90	113	160	134	114
A00102	-5190	117	101	95	60	55	57	37	63	67	77
A00102	-5180	65	62	56	44	56	65	68	67	53	67
A00102	-5170	80	78	89	96	84	68	66	81	87	104
A00102	-5160	98	83	101	93	81	72	119	60	71	78
A00102	-5150	80	115	133	97	111	127	119	78	117	100
A00102	-5140	120	162	222	186	151	116	143	136	144	135
A00102	-5130	119	108	90	81	74	73	115	114	111	96
A00102	-5120	131	176	191	120	208	135	89	149	136	999
A00103	-5212	238	267								
A00103	-5210	296	232	258	190	298	221	166	239	313	179
A00103	-5200	169	172	116	157	133	182	185	188	135	144
A00103	-5190	151	161	124	117	124	138	137	211	175	167
A00103	-5180	134	119	112	166	115	113	107	99	146	105
A00103	-5170	107	122	97	92	98	94	134	128	151	138
A00103	-5160	159	144	153	125	89	110	122	96	95	87
A00103	-5150	79	90	112	97	81	104	98	93	115	106
A00103	-5140	83	105	134	92	110	125	118	104	98	98
A00103	-5130	94	81	93	92	97	99	118	146	168	137
A00103	-5120	122	104	118	121	130	147	208	175	140	160
A00103	-5110	999									
A00104	-5199	343	167	238	156	204	228	193	270	257	
A00104	-5190	278	294	108	99	143	224	184	304	233	194

A00104	-5180	141	126	89	214	123	113	110	98	114	94
A00104	-5170	91	103	57	70	82	70	114	105	109	121
A00104	-5160	104	91	108	93	76	62	116	88	105	89
A00104	-5150	93	136	124	98	84	112	130	94	108	102
A00104	-5140	96	93	119	77	101	110	115	95	95	92
A00104	-5130	83	64	73	60	113	89	98	122	113	97
A00104	-5120	99	83	87	96	92	85	130	109	137	145
A00104	-5110	999									
A00105	-5199	194	129	226	116	149	201	218	175	162	
A00105	-5190	146	206	135	134	147	160	111	171	225	171
A00105	-5180	132	101	97	129	110	98	104	112	122	90
A00105	-5170	70	70	82	77	86	79	113	104	133	105
A00105	-5160	121	99	125	92	90	81	100	77	95	98
A00105	-5150	98	91	126	88	101	92	87	79	98	84
A00105	-5140	85	90	113	76	111	125	126	99	94	106
A00105	-5130	104	98	120	89	120	116	134	136	155	151
A00105	-5120	123	113	119	129	133	126	170	154	155	151
A00105	-5110	999									
A00106	-5201	217									
A00106	-5200	209	268	187	247	126	165	201	188	155	156
A00106	-5190	176	208	154	131	119	124	119	192	174	177
A00106	-5180	127	111	108	157	130	103	91	106	122	98
A00106	-5170	73	78	66	101	98	70	116	135	151	127
A00106	-5160	110	115	108	115	87	90	107	95	98	105
A00106	-5150	100	91	114	112	105	100	121	85	105	109
A00106	-5140	95	108	119	98	107	142	145	110	101	128
A00106	-5130	187	102	118	127	138	88	169	126	124	91
A00106	-5120	86	66	72	81	95	70	83	83	76	111
A00106	-5110	999									
A00107	-5211	139									
A00107	-5210	275	228	230	185	296	238	161	211	303	187
A00107	-5200	167	194	128	155	123	188	185	196	159	152
A00107	-5190	188	162	145	128	123	127	131	208	144	172
A00107	-5180	134	108	104	157	128	97	117	114	151	124
A00107	-5170	109	125	93	103	95	93	133	128	137	134
A00107	-5160	122	106	155	119	84	99	122	114	119	100
A00107	-5150	98	97	132	132	88	136	133	98	141	86
A00107	-5140	101	95	152	88	113	125	137	80	96	114
A00107	-5130	105	90	111	94	125	108	122	162	181	136
A00107	-5120	127	105	116	123	123	145	207	173	145	90
A00107	-5110	999									
A00108	-5219	271	122	159	166	102	145	98	125	43	
A00108	-5210	67	107	98	64	104	117	69	105	92	102
A00108	-5200	118	85	47	70	73	72	91	87	100	87
A00108	-5190	113	94	62	72	68	68	52	78	81	78
A00108	-5180	105	76	66	65	75	83	79	80	106	73
A00108	-5170	97	97	103	112	69	73	76	79	77	92
A00108	-5160	66	71	85	68	86	78	126	98	68	114
A00108	-5150	100	127	126	118	120	152	140	114	133	121
A00108	-5140	112	156	230	113	146	90	118	107	106	104
A00108	-5130	80	98	89	81	77	54	120	117	107	89
A00108	-5120	80	104	118	85	120	97	76	62	77	99
A00108	-5110	999									
A00109	-5170	42	78	127	102	91	78	77	80	105	106
A00109	-5160	93	96	96	112	73	95	140	104	129	126
A00109	-5150	106	158	178	151	159	122	146	91	176	116
A00109	-5140	167	179	288	215	185	134	225	206	217	130
A00109	-5130	149	156	117	119	107	133	185	150	131	104
A00109	-5120	163	180	219	141	240	154	158	197	128	122
A00109	-5110	211	185	123	999						
A00110	-5173	432	432	298							
A00110	-5170	304	198	188	169	197	162	233	232	258	206
A00110	-5160	275	144	165	189	175	177	320	332	188	260
A00110	-5150	297	334	357	456	229	139	125	67	155	216
A00110	-5140	196	314	365	206	206	229	286	274	235	221
A00110	-5130	240	164	185	152	271	240	127	80	91	88
A00110	-5120	130	129	232	213	290	253	293	221	250	333
A00110	-5110	356	433	315	312	999					
A00111	-5201	62									
A00111	-5200	83	102	84	51	77	114	64	91	99	91
A00111	-5190	98	81	62	64	56	51	36	60	71	80
A00111	-5180	59	64	39	68	128	96	97	58	65	44
A00111	-5170	47	58	61	69	52	72	90	99	91	106

A00111	-5160	126	88	88	98	82	65	130	89	82	131
A00111	-5150	100	148	177	142	184	173	142	107	153	88
A00111	-5140	130	167	289	170	194	115	179	149	166	122
A00111	-5130	134	97	97	97	79	96	110	154	84	77
A00111	-5120	127	134	175	103	156	109	116	115	85	88
A00111	-5110	104	182	166	102	999					
A00112	-5153	900	731	641							
A00112	-5150	948	876	864	893	661	290	265	122	250	274
A00112	-5140	139	168	309	160	134	178	294	225	192	178
A00112	-5130	178	157	148	134	251	127	64	82	103	102
A00112	-5120	200	127	263	194	276	229	319	256	239	319
A00112	-5110	421	546	335	382	186	231	999			
A00113	-5227	162	104	184	124	148	167	98			
A00113	-5220	185	187	152	189	171	143	148	130	110	108
A00113	-5210	128	152	149	143	193	219	128	152	145	126
A00113	-5200	168	99	79	107	113	85	111	107	129	103
A00113	-5190	121	108	70	79	66	60	38	45	61	83
A00113	-5180	74	43	66	45	57	58	72	47	69	74
A00113	-5170	56	86	81	93	76	68	71	91	116	119
A00113	-5160	99	111	90	95	95	98	99	92	86	110
A00113	-5150	117	228	125	115	109	135	129	115	130	93
A00113	-5140	110	123	207	167	142	105	114	95	106	90
A00113	-5130	95	88	70	72	51	58	84	81	112	97
A00113	-5120	177	179	210	107	211	101	113	147	126	128
A00113	-5110	156	133	124	176	87	106	999			
A00114	-5174	101	84	115	117						
A00114	-5170	104	120	164	153	113	135	131	146	125	180
A00114	-5160	120	145	128	148	108	113	154	107	127	161
A00114	-5150	135	194	172	182	234	218	162	101	141	77
A00114	-5140	120	174	298	172	171	120	157	176	163	169
A00114	-5130	163	115	124	96	79	78	205	174	153	101
A00114	-5120	162	254	209	107	256	148	119	163	137	160
A00114	-5110	195	167	173	168	99	106	999			
A00115	-5211	138									
A00115	-5210	111	135	124	147	179	162	121	134	133	155
A00115	-5200	154	103	97	102	118	82	101	104	98	104
A00115	-5190	98	86	84	73	57	55	38	55	57	83
A00115	-5180	70	59	44	46	46	57	57	46	62	62
A00115	-5170	62	73	88	80	68	64	69	85	88	101
A00115	-5160	140	85	83	66	74	119	110	60	76	83
A00115	-5150	106	242	163	115	99	133	125	96	133	88
A00115	-5140	99	122	203	160	129	88	136	99	118	92
A00115	-5130	97	88	103	72	56	53	66	85	132	95
A00115	-5120	294	226	234	114	196	144	137	172	178	137
A00115	-5110	129	115	101	158	87	82	999			
A00116	-5354	119	148	148	160						
A00116	-5350	164	106	183	129	73	124	92	102	125	63
A00116	-5340	57	91	98	114	121	135	153	145	144	142
A00116	-5330	324	250	489	389	251	144	99	102	102	74
A00116	-5320	108	104	93	79	68	52	39	37	47	43
A00116	-5310	71	59	77	58	54	81	64	58	67	97
A00116	-5300	75	96	94	80	62	36	73	70	101	103
A00116	-5290	110	96	96	110	82	100	77	67	99	127
A00116	-5280	122	128	264	245	169	140	185	230	236	287
A00116	-5270	190	250	349	347	167	306	484	406	349	295
A00116	-5260	387	115	134	174	120	134	99	90	156	152
A00116	-5250	143	93	64	94	85	97	165	153	143	139
A00116	-5240	193	105	138	222	137	90	119	92	140	84
A00116	-5230	165	208	114	130	123	205	146	176	191	113
A00116	-5220	174	198	146	216	188	135	208	149	129	80
A00116	-5210	107	115	146	99	248	197	119	145	150	125
A00116	-5200	160	111	69	75	76	77	77	109	103	104
A00116	-5190	124	108	78	68	58	69	61	110	106	116
A00116	-5180	89	87	67	92	69	131	133	104	123	101
A00116	-5170	91	108	128	95	94	90	95	93	122	135
A00116	-5160	107	128	77	121	88	123	134	87	109	115
A00116	-5150	97	149	178	168	176	185	201	112	170	171
A00116	-5140	149	205	363	146	232	143	232	178	179	183
A00116	-5130	152	128	129	126	121	120	176	156	112	104
A00116	-5120	163	207	188	176	328	160	218	213	164	161
A00116	-5110	226	141	135	109	71	50	80	93	999	
A00117	-5154	166	143	136	149						
A00117	-5150	154	222	233	198	204	180	138	104	136	110

A00117	-5140	146	253	370	253	203	153	236	166	185	134
A00117	-5130	161	138	119	85	75	71	158	147	150	110
A00117	-5120	169	204	231	167	324	193	191	185	145	155
A00117	-5110	180	188	144	166	92	74	40	31	47	999
A00118	-5168	151	183	185	153	130	162	90	131		
A00118	-5160	94	106	98	89	59	81	101	81	78	92
A00118	-5150	97	113	117	120	84	139	102	97	135	84
A00118	-5140	104	125	114	113	115	74	86	82	101	81
A00118	-5130	84	73	68	60	48	50	87	106	90	68
A00118	-5120	80	102	142	76	137	108	107	113	102	109
A00118	-5110	107	97	78	96	50	68	33	24	28	999
A00119	-5161	81									
A00119	-5160	58	68	56	74	59	67	125	102	92	100
A00119	-5150	92	136	126	129	110	120	124	117	143	155
A00119	-5140	159	183	281	171	191	136	216	121	146	117
A00119	-5130	138	100	113	114	119	118	141	131	110	96
A00119	-5120	132	175	190	153	224	138	151	180	101	159
A00119	-5110	179	147	114	114	64	52	49	59	60	999
A00120	-5357	97	160	208	213	189	139	267			
A00120	-5350	216	131	175	98	127	112	65	151	152	89
A00120	-5340	101	167	195	137	124	142	159	127	189	104
A00120	-5330	302	233	443	201	177	162	86	194	140	97
A00120	-5320	107	151	229	132	125	123	116	67	64	69
A00120	-5310	95	88	86	125	101	78	60	72	77	121
A00120	-5300	79	76	81	64	49	43	65	59	98	139
A00120	-5290	110	134	114	141	116	101	50	76	71	95
A00120	-5280	81	92	261	216	212	219	297	356	345	453
A00120	-5270	305	277	313	347	190	239	369	360	288	189
A00120	-5260	194	101	116	157	107	95	76	124	102	107
A00120	-5250	130	110	110	145	100	100	130	163	155	130
A00120	-5240	165	116	154	196	180	136	120	105	195	113
A00120	-5230	234	245	155	111	120	146	152	148	221	119
A00120	-5220	191	251	131	211	211	153	179	119	106	62
A00120	-5210	71	90	116	86	125	159	91	181	127	105
A00120	-5200	173	123	74	106	131	78	117	101	93	97
A00120	-5190	96	98	56	49	58	52	42	79	67	64
A00120	-5180	74	62	54	66	56	52	66	48	46	44
A00120	-5170	47	57	79	78	62	68	72	84	98	99
A00120	-5160	87	82	89	92	78	94	159	104	88	140
A00120	-5150	111	145	170	127	159	186	172	119	173	90
A00120	-5140	125	172	242	209	181	150	182	181	170	139
A00120	-5130	156	159	105	84	90	97	140	181	144	98
A00120	-5120	158	165	165	96	169	106	71	115	108	91
A00120	-5110	86	134	100	112	70	62	46	45	52	999
A00121	-5188	70	71	66	112	82	120	105	92		
A00121	-5180	93	98	73	107	99	93	101	80	96	73
A00121	-5170	93	106	108	94	102	89	88	84	137	139
A00121	-5160	94	125	109	109	104	82	175	148	130	150
A00121	-5150	108	230	178	135	183	68	104	79	145	101
A00121	-5140	110	126	236	165	148	86	110	130	166	114
A00121	-5130	94	123	105	82	130	83	123	154	102	90
A00121	-5120	99	121	110	111	194	124	123	133	100	130
A00121	-5110	123	139	103	95	99	153	90	105	112	999
A00122	-5214	163	136	127	105						
A00122	-5210	106	117	112	81	130	137	85	140	152	133
A00122	-5200	171	90	78	112	104	93	111	130	126	109
A00122	-5190	154	119	84	70	71	68	63	101	108	117
A00122	-5180	84	87	63	77	81	84	110	67	105	98
A00122	-5170	96	91	115	124	146	104	115	129	140	195
A00122	-5160	262	230	212	266	174	180	312	244	236	295
A00122	-5150	285	472	371	340	377	362	388	296	286	342
A00122	-5140	237	336	522	361	358	231	332	324	383	243
A00122	-5130	208	120	76	88	134	121	136	148	126	94
A00122	-5120	155	203	198	118	169	133	61	61	77	87
A00122	-5110	102	142	127	145	101	63	54	36	40	36
A00122	-5100	999									
A00123	-5200	150	107	162	177	123	96	104	121	119	117
A00123	-5190	167	85	65	72	68	53	79	63	94	91
A00123	-5180	81	71	83	94	117	106	91	53	51	51
A00123	-5170	70	90	95	85	103	90	103	150	116	154
A00123	-5160	121	130	99	110	97	83	174	94	98	155
A00123	-5150	167	211	142	135	162	151	211	83	153	125
A00123	-5140	137	151	237	149	132	98	154	163	221	133

A00123	-5130	125	120	95	80	96	117	100	128	77	92
A00123	-5120	118	121	102	92	226	118	110	137	134	116
A00123	-5110	193	135	116	116	101	165	76	66	75	82
A00123	-5100	999									
A00124	-5151	108									
A00124	-5150	101	158	194	157	163	138	149	91	151	113
A00124	-5140	177	132	296	233	215	149	192	202	288	186
A00124	-5130	231	186	196	143	134	127	192	167	132	113
A00124	-5120	226	253	241	131	275	142	133	138	129	115
A00124	-5110	142	130	131	112	82	65	72	90	62	29
A00124	-5100	40	999								
A00125	-5186	101	88	65	129	111	128				
A00125	-5180	118	70	72	81	84	123	117	92	144	102
A00125	-5170	123	109	144	124	111	123	79	118	118	130
A00125	-5160	98	107	85	126	86	92	179	106	117	137
A00125	-5150	138	227	181	180	223	179	217	127	266	173
A00125	-5140	169	230	441	160	196	118	213	186	292	154
A00125	-5130	186	123	113	111	143	130	129	147	91	94
A00125	-5120	210	208	214	135	231	141	238	177	117	99
A00125	-5110	151	111	116	104	72	55	66	73	106	82
A00125	-5100	57	93	999							
B_0001	-5365	116	125	113	116	98					
B_0001	-5360	86	83	73	91	148	128	126	90	84	130
B_0001	-5350	172	141	126	80	146	134	97	109	122	89
B_0001	-5340	73	68	93	79	81	105	95	73	75	85
B_0001	-5330	93	100	54	39	61	59	55	55	67	61
B_0001	-5320	80	104	118	136	84	57	65	86	72	62
B_0001	-5310	65	75	92	103	99	129	98	75	78	75
B_0001	-5300	74	72	76	87	78	80	109	104	117	100
B_0001	-5290	112	96	107	95	101	86	75	70	77	94
B_0001	-5280	109	133	132	113	126	93	93	84	78	110
B_0001	-5270	100	84	126	92	132	126	145	152	121	135
B_0001	-5260	94	70	73	110	121	120	97	71	75	102
B_0001	-5250	113	113	100	131	83	72	72	81	87	84
B_0001	-5240	101	102	999							
B_0002	-5341	75									
B_0002	-5340	71	64	84	77	74	99	84	66	76	70
B_0002	-5330	77	93	59	57	75	57	62	64	72	65
B_0002	-5320	91	102	95	119	97	95	113	92	98	68
B_0002	-5310	89	86	102	120	105	144	149	78	109	80
B_0002	-5300	82	96	96	89	95	88	93	118	143	101
B_0002	-5290	125	114	127	101	105	87	82	89	118	94
B_0002	-5280	102	122	132	125	128	67	86	79	93	98
B_0002	-5270	96	89	102	98	91	112	119	94	136	115
B_0002	-5260	101	75	91	104	106	98	76	58	68	80
B_0002	-5250	71	55	66	84	999					
B_0003	-5468	40	114	136	93	112	93	94	137		
B_0003	-5460	178	81	42	25	33	27	31	50	74	80
B_0003	-5450	94	78	76	127	147	108	58	58	64	56
B_0003	-5440	48	39	51	75	90	112	112	103	81	87
B_0003	-5430	68	64	56	53	37	34	26	40	80	169
B_0003	-5420	123	123	91	54	48	64	79	182	150	109
B_0003	-5410	109	97	83	75	139	86	79	95	128	264
B_0003	-5400	143	135	104	111	179	186	191	156	91	69
B_0003	-5390	67	57	108	74	65	69	100	74	124	67
B_0003	-5380	61	58	57	64	82	64	63	77	93	80
B_0003	-5370	110	81	89	71	90	90	80	97	150	120
B_0003	-5360	111	83	103	99	135	125	102	70	87	117
B_0003	-5350	126	104	93	62	96	105	76	93	115	80
B_0003	-5340	75	74	99	92	102	111	102	78	86	76
B_0003	-5330	86	95	57	56	76	63	57	63	69	64
B_0003	-5320	85	110	99	137	94	76	90	94	80	61
B_0003	-5310	62	63	66	63	69	82	80	68	70	62
B_0003	-5300	59	59	66	67	75	68	82	89	105	95
B_0003	-5290	110	87	107	84	94	75	62	55	77	82
B_0003	-5280	87	104	98	98	103	78	70	73	75	90
B_0003	-5270	87	75	85	89	98	102	120	116	118	102
B_0003	-5260	82	71	72	91	90	83	82	66	64	74
B_0003	-5250	78	84	75	74	71	81	98	77	71	65
B_0003	-5240	71	127	83	125	62	84	999			
B_0004	-5447	160	163	140	91	184	278	210			
B_0004	-5440	125	120	150	161	165	93	115	147	104	95
B_0004	-5430	93	86	75	63	48	38	55	50	66	82

B_0004	-5420	86	85	79	58	47	63	80	149	122	107
B_0004	-5410	140	116	114	97	161	99	148	140	169	127
B_0004	-5400	84	109	105	138	167	138	158	113	106	74
B_0004	-5390	63	101	108	99	107	85	87	72	78	67
B_0004	-5380	64	68	71	68	87	58	62	51	63	49
B_0004	-5370	89	49	55	50	57	55	60	68	67	54
B_0004	-5360	59	50	72	80	103	117	91	70	58	73
B_0004	-5350	97	89	99	82	76	75	53	63	68	73
B_0004	-5340	48	49	64	57	72	74	71	59	59	59
B_0004	-5330	90	89	63	65	78	67	62	74	71	58
B_0004	-5320	70	63	49	51	52	50	57	59	63	57
B_0004	-5310	64	60	70	99	90	96	72	64	53	49
B_0004	-5300	56	65	76	75	68	63	53	69	75	74
B_0004	-5290	76	69	87	56	71	63	53	49	55	65
B_0004	-5280	59	68	63	67	78	47	52	109	103	102
B_0004	-5270	66	63	59	60	55	60	58	60	58	58
B_0004	-5260	59	48	51	59	56	47	50	45	47	64
B_0004	-5250	54	51	50	52	43	46	47	52	44	42
B_0004	-5240	40	47	53	46	45	38	41	33	31	42
B_0004	-5230	33	42	34	37	38	37	38	34	38	34
B_0004	-5220	35	47	999							
B_0005	-5351	34									
B_0005	-5350	64	80	94	110	88	77	60	78	70	70
B_0005	-5340	43	47	64	72	81	88	70	53	59	53
B_0005	-5330	98	91	84	78	101	52	54	62	62	55
B_0005	-5320	63	56	67	72	65	58	75	91	64	64
B_0005	-5310	82	91	91	84	90	88	75	70	55	65
B_0005	-5300	61	52	61	67	52	47	57	55	71	67
B_0005	-5290	58	63	59	43	59	51	50	49	40	54
B_0005	-5280	62	47	55	52	62	53	42	45	52	76
B_0005	-5270	57	75	75	70	74	67	90	76	95	98
B_0005	-5260	83	76	69	56	49	52	47	45	53	63
B_0005	-5250	70	59	58	54	76	63	44	59	44	55
B_0005	-5240	52	47	59	61	64	50	50	66	56	64
B_0005	-5230	61	55	76	82	76	69	62	57	56	61
B_0005	-5220	65	77	67	44	55	37	31	27	36	50
B_0005	-5210	67	999								
B_0006	-5346	167	94	69	88	113	80				
B_0006	-5340	58	82	97	89	100	130	92	67	73	70
B_0006	-5330	100	113	93	116	153	91	78	84	84	71
B_0006	-5320	80	80	84	85	79	71	85	100	83	73
B_0006	-5310	105	93	92	110	110	100	90	76	75	64
B_0006	-5300	67	61	81	77	57	60	64	78	96	79
B_0006	-5290	69	73	79	56	64	59	58	46	50	66
B_0006	-5280	69	75	67	78	87	77	80	59	61	65
B_0006	-5270	75	82	86	89	82	91	88	91	103	126
B_0006	-5260	119	102	83	79	69	66	65	52	65	74
B_0006	-5250	82	75	70	77	90	75	76	83	63	65
B_0006	-5240	72	55	68	73	88	999				
E1_001	-5189	486	384	274	632	391	169	304	276	223	
E1_001	-5180	212	224	172	383	267	281	247	208	177	132
E1_001	-5170	129	101	117	121	116	148	112	140	277	531
E1_001	-5160	549	352	501	445	407	173	126	105	94	96
E1_001	-5150	169	245	226	293	647	698	677	206	195	240
E1_001	-5140	158	128	171	115	94	106	201	228	242	159
E1_001	-5130	262	195	219	208	231	177	281	257	329	168
E1_001	-5120	90	99	86	86	167	157	197	133	129	100
E1_001	-5110	186	264	200	165	123	215	158	200	363	201
E1_001	-5100	212	166	151	999						
E1_002	-5185	294	272	291	347	410					
E1_002	-5180	453	370	174	414	271	347	281	383	311	295
E1_002	-5170	227	216	355	311	163	234	129	161	163	244
E1_002	-5160	220	126	173	179	192	107	95	96	121	177
E1_002	-5150	175	205	213	159	248	331	266	111	151	177
E1_002	-5140	153	131	153	180	144	145	260	240	238	151
E1_002	-5130	229	179	191	203	166	197	228	193	236	134
E1_002	-5120	92	67	77	91	108	143	93	95	106	141
E1_002	-5110	112	94	104	116	89	128	146	135	220	162
E1_002	-5100	136	135	160	999						
E1_003	-5196	540	193	282	350	302	278				
E1_003	-5190	295	418	269	203	374	166	131	275	190	239
E1_003	-5180	179	186	136	296	233	226	215	180	168	141
E1_003	-5170	135	131	194	184	135	201	107	153	203	298

E1_003	-5160	318	125	140	174	125	95	106	79	82	97
E1_003	-5150	174	299	372	213	395	539	415	133	246	251
E1_003	-5140	240	88	106	89	98	111	275	336	287	198
E1_003	-5130	331	232	245	232	233	318	351	292	423	187
E1_003	-5120	66	62	82	110	153	116	227	147	124	110
E1_003	-5110	116	173	162	157	108	180	174	178	432	249
E1_003	-5100	263	200	200	999						
E1_004	-5186	587	292	170	321	231	158				
E1_004	-5180	217	311	154	283	211	365	211	265	223	223
E1_004	-5170	206	154	244	200	163	232	133	166	201	319
E1_004	-5160	431	324	483	309	262	132	138	102	101	133
E1_004	-5150	158	289	282	342	631	685	361	169	214	395
E1_004	-5140	233	128	193	126	106	127	248	276	210	182
E1_004	-5130	322	221	264	253	239	313	361	364	470	191
E1_004	-5120	87	62	79	85	172	136	158	144	96	100
E1_004	-5110	243	381	194	164	129	167	116	155	322	203
E1_004	-5100	182	171	218	999						
E1_005	-5212	65	128								
E1_005	-5210	161	195	263	309	477	681	597	526	744	791
E1_005	-5200	665	433	527	521	575	198	294	286	390	294
E1_005	-5190	398	480	359	264	491	278	223	322	205	226
E1_005	-5180	164	191	143	384	246	188	208	153	174	119
E1_005	-5170	119	120	167	189	160	144	112	136	160	290
E1_005	-5160	306	232	177	206	203	146	101	64	92	124
E1_005	-5150	202	323	313	326	428	387	414	155	186	294
E1_005	-5140	174	154	139	107	99	93	127	173	254	192
E1_005	-5130	316	245	258	233	222	219	260	251	360	196
E1_005	-5120	79	63	66	84	122	123	152	148	105	129
E1_005	-5110	171	211	154	167	121	121	149	160	364	239
E1_005	-5100	274	178	185	999						
E1_006	-5188	409	222	407	303	323	451	529	688		
E1_006	-5180	665	654	296	574	411	525	398	629	458	348
E1_006	-5170	328	324	402	261	194	212	138	165	196	250
E1_006	-5160	285	164	217	205	191	105	104	121	122	132
E1_006	-5150	168	217	213	165	277	222	325	123	119	178
E1_006	-5140	117	119	173	142	118	91	200	244	194	135
E1_006	-5130	157	126	139	123	151	188	269	226	262	180
E1_006	-5120	72	45	73	113	97	127	143	999		
E1_007	-5192	175	262								
E1_007	-5190	492	612	420	298	404	182	149	174	245	260
E1_007	-5180	312	307	179	246	214	241	253	376	249	278
E1_007	-5170	242	214	307	246	186	193	138	188	211	260
E1_007	-5160	217	177	205	146	139	81	118	99	78	120
E1_007	-5150	141	167	198	194	343	293	229	97	143	177
E1_007	-5140	154	148	267	183	143	113	230	184	199	150
E1_007	-5130	275	222	216	194	198	242	292	224	269	166
E1_007	-5120	92	50	55	71	118	87	110	999		
E1_008	-5186	461	253	237	354	316	368				
E1_008	-5180	289	298	153	346	244	284	314	233	249	200
E1_008	-5170	215	224	293	195	118	144	111	131	145	172
E1_008	-5160	187	101	210	178	150	81	91	81	92	139
E1_008	-5150	156	238	227	206	352	351	293	112	191	145
E1_008	-5140	153	72	123	110	107	109	242	234	206	160
E1_008	-5130	261	204	229	235	219	217	258	245	294	143
E1_008	-5120	86	69	81	92	132	117	143	123	88	94
E1_008	-5110	87	115	93	122	92	142	126	127	166	145
E1_008	-5100	114	161	152	999						
E1_009	-5167	151	168	137	110	134	176	202			
E1_009	-5160	221	118	161	169	161	83	73	80	93	188
E1_009	-5150	134	215	185	146	384	306	336	146	220	174
E1_009	-5140	151	110	133	106	72	109	173	177	159	176
E1_009	-5130	212	248	183	190	214	223	312	239	307	188
E1_009	-5120	93	107	73	999						
E1_010	-5196	406	163	327	348	354	304				
E1_010	-5190	223	393	272	201	440	161	110	168	90	111
E1_010	-5180	61	115	73	280	163	185	113	180	141	102
E1_010	-5170	98	120	201	202	153	188	85	150	220	210
E1_010	-5160	317	149	211	164	212	124	117	83	75	123
E1_010	-5150	157	340	310	334	549	535	302	108	198	204
E1_010	-5140	137	138	112	100	77	105	212	261	266	162
E1_010	-5130	319	279	327	285	322	361	465	316	438	154
E1_010	-5120	79	61	60	64	86	90	165	119	83	117
E1_010	-5110	197	222	184	162	140	178	171	167	322	234

E1_010	-5100	289	239	204	999						
E1_011	-5186	469	290	221	370	373	384				
E1_011	-5180	271	291	197	353	275	389	265	245	230	232
E1_011	-5170	241	164	214	216	108	128	106	132	179	241
E1_011	-5160	210	153	220	222	331	116	157	148	163	269
E1_011	-5150	269	344	394	300	387	465	369	150	266	202
E1_011	-5140	192	143	261	164	126	109	237	216	234	201
E1_011	-5130	241	184	229	205	203	213	330	220	300	179
E1_011	-5120	61	60	73	69	141	110	189	136	112	114
E1_011	-5110	128	202	195	209	164	180	156	211	240	194
E1_011	-5100	179	161	999							
E1_012	-5183	435	260	380							
E1_012	-5180	298	252	196	325	204	375	243	227	220	210
E1_012	-5170	222	214	329	316	223	183	148	233	248	327
E1_012	-5160	339	145	198	230	176	68	110	124	109	124
E1_012	-5150	121	169	281	194	271	315	303	140	149	199
E1_012	-5140	180	94	108	134	89	118	178	249	196	166
E1_012	-5130	242	227	234	197	175	227	288	255	290	182
E1_012	-5120	60	69	69	88	160	135	215	144	134	112
E1_012	-5110	94	98	113	131	85	124	135	199	188	178
E1_012	-5100	177	120	187	999						
E1_013	-5174	455	316	301	261						
E1_013	-5170	199	181	315	274	216	239	125	189	217	261
E1_013	-5160	252	126	206	207	99	70	71	79	77	121
E1_013	-5150	108	152	233	127	236	297	404	112	116	125
E1_013	-5140	141	80	124	104	116	78	157	246	152	136
E1_013	-5130	216	229	166	145	151	169	271	194	221	159
E1_013	-5120	79	64	106	144	167	133	148	108	101	104
E1_013	-5110	169	144	112	104	81	117	98	117	165	135
E1_013	-5100	183	187	187	999						
E1_014	-5173	221	234	166							
E1_014	-5170	171	186	313	261	115	155	122	135	158	229
E1_014	-5160	261	147	205	197	149	110	111	104	119	151
E1_014	-5150	102	193	192	132	217	204	260	126	180	104
E1_014	-5140	117	87	100	100	74	95	230	190	188	150
E1_014	-5130	184	115	157	153	133	154	233	181	259	150
E1_014	-5120	107	68	102	120	132	152	151	108	85	123
E1_014	-5110	118	132	138	91	127	118	184	210	195	999
E1_015	-5189	383	287	199	343	127	107	192	120	171	
E1_015	-5180	106	149	116	301	134	154	126	204	212	150
E1_015	-5170	210	174	229	173	133	193	71	138	180	272
E1_015	-5160	340	209	263	266	223	151	136	104	117	199
E1_015	-5150	186	444	345	344	564	570	379	158	206	298
E1_015	-5140	199	139	150	109	101	88	197	206	263	166
E1_015	-5130	299	243	252	221	212	337	408	315	319	181
E1_015	-5120	59	54	57	67	84	92	190	112	95	127
E1_015	-5110	235	276	226	205	157	235	145	194	367	284
E1_015	-5100	273	999								
E1_016	-5179	270	187	391	310	278	272	240	248	252	
E1_016	-5170	233	288	344	301	233	244	142	190	221	302
E1_016	-5160	266	156	199	213	173	92	96	97	131	193
E1_016	-5150	164	306	300	280	374	427	364	139	204	166
E1_016	-5140	189	84	175	125	95	110	251	276	276	248
E1_016	-5130	379	219	170	174	170	254	278	264	311	243
E1_016	-5120	81	65	98	106	135	122	247	181	97	153
E1_016	-5110	103	164	999							
E1_017	-5187	88	169	103	133	155	151	171			
E1_017	-5180	195	253	127	364	194	266	210	215	177	193
E1_017	-5170	203	205	274	270	141	209	109	142	227	221
E1_017	-5160	243	114	162	170	103	70	110	113	124	136
E1_017	-5150	149	198	319	156	323	315	216	82	139	131
E1_017	-5140	115	89	122	103	80	72	139	128	119	113
E1_017	-5130	211	216	132	181	132	129	197	147	237	142
E1_017	-5120	59	66	105	82	124	131	182	999		
E1_018	-5188	444	359	397	217	170	214	273	349		
E1_018	-5180	317	296	144	367	231	261	301	159	201	149
E1_018	-5170	137	169	177	161	125	131	103	137	159	120
E1_018	-5160	164	109	183	164	144	89	126	89	81	96
E1_018	-5150	85	130	157	147	249	321	216	83	184	181
E1_018	-5140	144	109	152	160	126	122	201	233	184	154
E1_018	-5130	278	213	222	211	163	184	251	208	310	194
E1_018	-5120	69	62	65	88	135	162	163	999		
E1_019	-5172	244	321								

E1_019	-5170	297	256	466	310	215	237	129	180	229	233
E1_019	-5160	222	136	198	204	122	85	117	109	118	141
E1_019	-5150	161	195	350	202	311	375	358	166	200	156
E1_019	-5140	154	94	179	136	115	121	180	217	170	155
E1_019	-5130	240	199	179	191	186	211	315	247	219	188
E1_019	-5120	75	74	102	190	165	158	173	128	143	118
E1_019	-5110	185	999								
E2_001	-5375	216	179	153	195	211					
E2_001	-5370	266	231	208	353	314	228	267	201	169	182
E2_001	-5360	111	185	244	232	229	269	208	205	140	250
E2_001	-5350	316	279	403	222	273	275	229	195	269	196
E2_001	-5340	180	178	189	200	129	212	140	119	136	116
E2_001	-5330	168	140	150	130	166	113	89	138	169	119
E2_001	-5320	128	173	147	225	174	116	184	162	148	152
E2_001	-5310	162	189	179	206	275	207	187	134	198	130
E2_001	-5300	207	180	200	275	145	192	180	191	216	271
E2_001	-5290	205	202	194	213	206	173	153	149	137	202
E2_001	-5280	210	242	284	207	179	196	166	197	170	178
E2_001	-5270	220	166	167	237	164	134	199	175	181	157
E2_001	-5260	162	139	134	109	125	116	110	129	133	166
E2_001	-5250	130	124	87	116	103	106	110	120	104	110
E2_001	-5240	114	103	129	108	146	103	174	129	119	112
E2_001	-5230	156	162	108	113	133	999				
E2_002	-5337	358	242	380	224	196	215	198			
E2_002	-5330	313	309	366	258	277	193	160	252	339	205
E2_002	-5320	210	250	221	306	202	191	267	225	180	221
E2_002	-5310	177	204	240	301	301	262	201	149	179	190
E2_002	-5300	242	226	244	250	160	179	207	199	239	268
E2_002	-5290	211	206	223	204	230	191	169	207	188	212
E2_002	-5280	157	233	196	216	232	179	146	163	149	163
E2_002	-5270	129	73	107	125	96	102	128	109	125	121
E2_002	-5260	141	101	89	106	105	108	114	123	127	137
E2_002	-5250	113	117	105	91	133	115	125	167	150	145
E2_002	-5240	168	131	147	150	164	130	233	166	147	145
E2_002	-5230	197	192	148	150	172	169	163	184	175	164
E2_002	-5220	115	157	146	191	227	999				
